# 13th International Conference on Conservative Management of Spinal Deformities and First Joint Meeting of the International Research Society on Spinal Deformities and the Society on Scoliosis Orthopaedic and Rehabilitation Treatment – SOSORT-IRSSD 2016 meeting: Banff, Canada. 25-28 May 2016

**DOI:** 10.1186/s13013-017-0124-0

**Published:** 2017-05-30

**Authors:** Aria Bagheri, Xue-Cheng Liu, Channing Tassone, John Thometz, Amie Chaloupka, Sergey Tarima, Larry Cohen, Milena Simic, Sarah Dennis, Kathryn Refshauge, Evangelos Pappas, Eric C. Parent, Matthew Pietrosanu, Emily Redford, Sheri Schmidt, Douglas Hill, Marc Moreau, Douglas Hedden, Samer Adeeb, Edmond Lou, Rob C. Brink, Tom P. C. Schlösser, Dino Colo, Koen L. Vincken, Marijn van Stralen, Steve C. N. Hui, Winnie C. W. Chu, Jack C. Y. Cheng, René M. Castelein, Vasileios Kechagias, Theodoros B. Grivas, Konstantinos Vlasis, Konstantinos Michas, Theodoros B. Grivas, Vasileios Kechagias, Konstantinos Vlasis, Konstantinos Michas, Elisa M. S. Tam, Fiona W. P. Yu, Vivian W. Y. Hung, Lin Shi, Ling Qin, Bobby K. W. Ng, Winnie C. W. Chu, James Griffith, Jack C. Y. Cheng, Tsz Ping Lam, Cindy Xue, Lin Shi, Steve C. N. Hui, Tsz Ping Lam, Bobby K. W. Ng, Jack C. Y. Cheng, Winnie C. W. Chu, Steve C. N. Hui, Jean-Philippe Pialasse, Judy Y. H. Wong, Tsz Ping Lam, Bobby K. W. Ng, Jack C. Y. Cheng, Winnie C. W. Chu, Quang N. Vo, Lawrence H. Le, Edmond H. M. Lou, Rui Zheng, Douglas L. Hill, Marc J. Moreau, Douglas M. Hedden, James K. Mahood, Sarah Southon, Edmond Lou, Arnaud Brignol, Farida Cheriet, Marie-Claude Miron, Catherine Laporte, Yong Qiu, Hao Liu, Zhen Liu, Ze-zhang Zhu, Bang-ping Qian, XueCheng Liu, Robert Rizza, John Thometz, Derek Rosol, Channing Tassone, Sergey Tarima, Paula North, Fabio Zaina, Francesca Pesenti, Stefano Negrini, Luca Persani, Paolo Capodaglio, Nicoletta Polli, Benjamin Hon Kei Yip, Fiona Wai Ping Yu, Vivian Wing Yin Hung, Tsz Ping Lam, Ling Qin, Bobby Kin Wah Ng, Jack Chun Yiu Cheng, Jiajun Zhang, Wayne Yuk Wai Lee, Huanxiong Chen, Elisa Man Shan Tam, Gene Chiwai Man, Tsz Ping Lam, Bobby Kin Wah Ng, Yong Qiu, Jack Chun Yiu Cheng, Hao Liu, Zhen Liu, Zezhang Zhu, Bang Ping Qian, Yong Qiu, P. Harasymczuk, M. Andrusiewicz, P. Janusz, P. Biecek, T. Kotwicki, M. Kotwicka, Jung Sub Lee, Jong Ki Shin, Tae Sik Goh, Seung Min Son, Huanxiong Chen, Wayne Yuk Wai Lee, Jiajun Zhang, Elisa Man Shan Tam, Gene Chi Wai Man, Tsz Ping Lam, Bobby Kin Wah Ng, Yong Qiu, Jack Chun Yiu Cheng, Mark Schwartz, Sarah Gilday, Donita I. Bylski-Austrow, David L. Glos, Lindsay Schultz, Sara O’Hara, Viral V. Jain, Peter F. Sturm, Xiaoyu Wang, Dennis G. Crandall, Stefan Parent, Noelle Larson, Hubert Labelle, Carl-Eric Aubin, Negar Behzadi Fard, Sarah Southon, Marc Moreau, Douglas Hedden, Kajsa Duke, Sarah Southon, Leeann Lukenchuk, Matthew Kerslake, Geraldine Huynh, Jill Chorney, Ban Tsui, Daniel Tobert, Prachi Bakarania, Hagit Berdishevsky, Kelly Grimes, Hiroko Matsumoto, Joshua Hyman, Benjamin Roye, David Roye, Michael Vitale, Jason Black, Michael Bradley, Shawn Drake, David Glynn, Erika Maude, Hagit Berdishevsky, Amelia Lindgren, Prachi Bakarania, Kelly Grimes, Hiroko Matsumoto, Nicholas Feinberg, Zachary Bloom, David Roye, Michael Vitale, Sarah Dupuis, Carole Fortin, Christiane Caouette, Carl-Éric Aubin, Gozde Gur, Yavuz Yakut, Nikola Jevtić, Sanja Schreiber, Axel Hennes, Milan Pantović, Jean-Claude de Mauroy, Frédéric Barral, Sophie Pourret, Jean-Claude de Mauroy, Frédéric Barral, Sophie Pourret, Angelo Gabriele Aulisa, Vincenzo Guzzanti, Marco Galli, Francesco Falciglia, Lorenzo Aulisa, Jean-Claude Bernard, Julie Deceuninck, Eric Berthonnaud, Adrien Rougelot, Marie-Eva Pickering, Emmanuelle Chaleat-Valayer, Richard Webb, Josette Bettany-Saltikov, Barbara Neil, Fabio Zaina, Martina Poggio, Sabrina Donzelli, Monia Lusini, Salvatore Minnella, Stefano Negrini, Jean-Claude de Mauroy, Frédéric Barral, Alith Hoang, Saihu Mao, Benlong Shi, Bangping Qian, Zezhang Zhu, Xu Sun, Yong Qiu, Nikita Cobetto, Carl-Éric Aubin, Stefan Parent, Soraya Barch, Isabelle Turgeon, Hubert Labelle, Hasan Md Arif Raihan, Datta Tarit Kumar, Chapal Khasnabis, Ameed Equbal, Ashis Kumar Chakraborty, Abhishek Biswas, Gozde Gur, Burcu Dilek, Cigdem Ayhan, Engin Simsek, Ozgen Aras, Songul Aksoy, Yavuz Yakut, Edmond Lou, Doug Hill, Rui Zheng, Andreas Donauer, Melissa Tilburn, Jim Raso, Marc Morau, Douglas Hedden, He Chen, Wong Man-Sang, Larry Cohen, Sarah Kobayashi, Milena Simic, Sarah Dennis, Kathryn Refshauge, Evangelos Pappas, Fatemeh Aslanzadeh, Eric C. Parent, Brian MacIntosh, Emmanouil G. Maragkoudakis, Theodoros B. Grivas, Ioannis D. Gelalis, Christina Mazioti, Gerasimos Tsilimidos, R. Geoffrey Burwell, Yu Zheng, Xiao-Jun Wu, Yi-Ni Dang, Ning Sun, Yan Yang, Tao Wang, Cheng-Qi He, Man-Sang Wong, Sabrina Donzelli, Gregorio Martinez, Alberto Negrini, Fabio Zaina, Stefano Negrini, Hiroko Matsumoto, Nicholas Feinberg, Matthew Shirley, Hasani Swindell, Zachary Bloom, David P. Roye, Behrooz A. Akbarnia, Sumeet Garg, James O. Sanders, David L. Skaggs, John T. Smith, Michael G. Vitale, Children’s Spine Study Group, Growing Spine Study Group, Robert Rizza, XueCheng Liu, John Thometz, Edmond Lou, Doug Hill, Andreas Donauer, Melissa Tilburn, Douglas Hedden, Marc Moreau, Aoife Healy, Sybil Farmer, Nachiappan Chockalingam, Angelo Gabriele Aulisa, Vincenzo Guzzanti, Marco Galli, Paolo Pizzetti, Lorenzo Aulisa, Toru Maruyama, Yosuke Kobayashi, Yusuke Nakao, Hao Liu, Bang Ping Qian, Yong Qiu, Sai-hu Mao, Bin Wang, Yang Yu, Zezhang Zhu, Hagit Berdishevsky, Amelia M. Lindgren, Prachi Bakarania, Kelly Grimes, Melvin C. Makhni, Jamal Shillingford, Michael G. Vitale, Jason Black, Erika Maude, Abbie Turland, David Glynn, Antonio Caronni, Luciana Sciumè, Sabrina Donzelli, Fabio Zaina, Stefano Negrini, Sanja Schreiber, Eric C. Parent, Elham Khodayari Moez, Douglas M. Hedden, Douglas L. Hill, Marc Moreau, Edmond Lou, Elise M. Watkins, Sarah C. Southon, Eric C. Parent, Sanja Schreiber, Elham Khodayari Moez, Preston Sloan, Douglass Hedden, Marc Moreau, Douglas Hill, Sarah Southon, Elise Watkins, Eric C. Parent, Maliheh Ghaneei, Samer Adeeb, Sanja Schreiber, Marc Moreau, Douglas Hedden, Douglas Hill, Sarah Southon, Nikos Karavidas, Despoina Dritsa, Josette Bettany-Saltikov, Nigel Hanchard, Donghyun Kim, Junlae Kim, Amy Sbihli, Eric Parent, Lauren Levey, Mark Holowka, Leigh Davis, Lori A Dolan, Stuart L. Weinstein, BrAIST Study Group, Jill E. Larson, Maximilian A. Meyer, Barrett Boody, John F. Sarwark, Sanja Schreiber, Eric C. Parent, Douglas M. Hedden, Douglas L. Hill, John Thometz, XueCheng Liu, Robert Rizza, Channing Tassone, XueCheng Liu, Benjamin Gundlach, Sergey Tarima, Alison Grant, Raman Kalyan, Waleed Hekal, Cheryl Honeyman, Tim Cook, Scott Murray, Morena Pitruzzella, Sabrina Donzelli, Fabio Zaina, Stefano Negrini, Jean-Claude de Mauroy, Frédéric Barral, Sophie Pourret, Jean-Claude de Mauroy, Frédéric Barral, Sophie Pourret, Kelly Grimes, Nicholas Feinberg, Jennifer Hope, Hagit Berdishevsky, Prachi Bakarania, Hiroko Matsumoto, Hasani Swindell, Julie Yoshimachi, David Roye, Michael Vitale, Julie Touchette, Anissa St-Jean, Danica Brousseau, Louise Marcotte, Jean Théroux, Chantal Doucet, Yangmin Lin, Man Sang Wong, John MacMahon, Edward MacMahon, Jeremy Boyette, Luke Stikeleather, Andrea Lebel, Victoria Ashley Lebel, Chintan A. Pancholi-Parekh, Lise Stolze, Marissa Selthafner, Kaitlin Hong, XueCheng Liu, John Thometz, Channing Tassone, Pamela R. Morrison, Timothy A. Hanke, Patrick Knott, Nathaniel D. Krumdick, Nachiappan Chockalingam, Thomas Shannon, Ryan Davenhill, Robert Needham, Vinay Jasani, El-Nasri Ahmed, Anissa St-Jean, Julie Touchette, Shawn Drake, Danica Brousseau, Louise Marcotte, Jean Théroux, Chantal Doucet, Angelo Gabriele Aulisa, Vincenzo Guzzanti, Marco Gordano, Giuseppe Mastantuoni, Lorenzo Aulisa, Michail Chandrinos, Theodoros B. Grivas, Vasileios Kechagias, Paweł Głowka, Dominik Gaweł, Bartosz Kasprzak, Michał Nowak, Marek Morzyński, Tomasz Kotwicki, Julie Deceuninck, Jean-Claude Bernard, Cyril Lecante, Eric Berthonnaud, Carole Fortin, Jean-François Aubin-Fournier, Josette Bettany-Saltikov, Eric C. Parent, Debbie Ehrmann Feldman, Jean-Claude Bernard, Zhen Liu, Wen Zhang, Zongshan Hu, Weiguo Zhu, Mengran Jin, Xiao Han, Yong Qiu, Jack C. Y. Cheng, Zezhang Zhu, Zhen Liu, Jing Guo, Tao Wu, Bangping Qian, Zezhang Zhu, Feng Zhu, Jian Jiang, Yong Qiu, Xiao Han, Zhen Liu, Hao Liu, Yong Qiu, Jing Guo, Huang Yan, Xu Sun, Jack C. Y. Cheng, Zezhang Zhu, Francesca Di Felice, Fabio Zaina, Morena Pitruzzella, Sabrina Donzelli, Stefano Negrini, Robert A Needham, Panagiotis Chatzistergos, Nachiappan Chockalingam, Rob C. Brink, Tom P. C. Schlösser, Dino Colo, Koen L. Vincken, Marijn van Stralen, Steve C. N. Hui, Winnie C. W. Chu, Jack C. Y. Cheng, René M. Castelein, Donita I. Bylski-Austrow, David L. Glos, Viral V. Jain, Joseph E. Reynolds, Peter F. Sturm, Eric J. Wall, Vasilios G. Igoumenou, Panayiotis D. Megaloikonomos, Konstantinos Tsiavos, Georgios N. Panagopoulos, Andreas F. Mavrogenis, Theodoros B. Grivas, Konstantinos Soultanis, Panayiotis J. Papagelopoulos, Negar Behzadi Fard, Kajsa Duke, Andrew Chan, Eric C. Parent, Edmond Lou, Jung Sub Lee, Jong Ki Shin, Tae Sik Goh, Seung Min Son, Sho Kobayashi, Daisuke Togawa, Tomohiko Hasegawa, Yu Yamato, Shin Oe, Tomohiro Banno, Yuuki Mihara, Yukihiro Matsuyama

**Affiliations:** 1 Division of Biostatistics, Medical College of Wisconsin, Milwaukee, WI USA; 2 Department of Orthopaedic Surgery, Milwaukee, WI USA; 3 Musculoskeletal Functional Assessment Center, Milwaukee, WI USA; 4 Children’s Hospital of Wisconsin, Milwaukee, WI USA; 5 The University of Sydney, Sydney, Australia; 6 University of Alberta, Edmonton, Canada; 7 Alberta Health Services, Edmonton, Canada; 8 Department of Orthopaedic Surgery, University Medical Center Utrecht, Utrcht, The Netherlands; 9 Image Sciences Institute, University Medical Center Utrecht, Utrecht, The Netherlands; 10 Department of Imaging & Interventional Radiology, Prince of Wales Hospital, The Chinese University of Hong Kong, Shatin, Hong Kong SAR; 11 Department of Orthopaedics and Traumatology, Prince of Wales Hospital, The Chinese University of Hong Kong, Shatin, Hong Kong SAR; 12 Private practice, Athens, Greece; 13 Department of Orthopaedics and Traumatology, Tzaneio General Hospital of Piraeus, Piraeus, Greece; 14 Department of Anatomy, Medical School, University of Athens, Athens, Greece; 15 Kimi Health Center, Kimi, Greece; 16 Department of Orthopaedics and Traumatology, Tzaneio General Hospital of Piraeus, Piraeus, Greece; 17 Private Practice, Athens, Greece; 18 Department of Anatomy, Medical School, University of Athens, Athens, Greece; 19 Kimi Health Center, Kimi, Greece; 20 Department of Orthopaedics & Traumatology, The Chinese University of Hong Kong, Shatin, Hong Kong SAR; 21 Bone Quality and Health Centre, Department of Orthopaedics & Traumatology, The Chinese University of Hong Kong, Shatin, Hong Kong SAR; 22 Department of Medicine and Therapeutics, The Chinese University of Hong Kong, Shatin,, Hong Kong SAR; 23 Department of Imaging & Interventional Radiology, Prince of Wales Hospital, The Chinese University of Hong Kong, Shatin, Hong Kong SAR; 24 Department of Imaging and Interventional Radiology, The Chinese University of Hong Kong, Shatin, Hong Kong SAR; 25 Department of Medicine & Therapeutics, The Chinese University of Hong Kong, Shatin, Hong Kong SAR; 26 Department of Orthopedics & Traumatology, The Chinese University of Hong Kong, Shatin, Hong Kong SAR; 27 Department of Imaging and Interventional Radiology, The Chinese University of Hong Kong, Shatin, Hong Kong SAR; 28 Department of Orthopedics & Traumatology, The Chinese University of Hong Kong, Shatin, Hong Kong SAR; 29 Department of Chiropractic, University of Quebec at Trois-Rivieres, Trois-Rivieres, Quebec Canada; 30 University of Alberta, Edmonton, Canada; 31 University of Alberta, Edmonton, Canada; 32 Alberta Health Services, Edmonton, Canada; 33 École de technologie supérieure, Montréal, Canada; 34 Polytechnique Montréal, Montréal, Canada; 35 CHU Sainte-Justine, Montréal, Québec Canada; 36 The Department of Spine Surgery, the Affiliated Drum Tower Hospital of Nanjing University Medical School, Nanjing, China; 37 Department of Orthopaedic Surgery, Medical College of Wisconsin, Milwaukee, WI USA; 38 Department of Mechanical Engineering, Milwaukee School of Engineering, Milwaukee, WI USA; 39 Division of Biostatistics, Medical College of Wisconsin, Milwaukee, WI USA; 40 Department of Pathology, Children’s Hospital of Wisconsin, Medical College of Wisconsin, Milwaukee, WI USA; 41 ISICO, Milan, Italy; 42 Division of Endocrine & Metabolic Disease, Istituto Auxologico Italiano, Milan, Italy; 43 Don Gnocchi Foundation, Brescia, Italy; 44 Brescia University, Brescia, Italy; 45 Rehabilitation Department, Istituto Auxologico Italiano, Verbania, Italy; 46 Department of Orthopaedics and Traumatology, Faculty of Medicine, Prince of Wales Hospital, The Chinese University of Hong Kong, Shatin, Hong Kong SAR; 47 School of Public Health and Primary Care, Faculty of Medicine, The Chinese University of Hong Kong, Shatin, Hong Kong SAR; 48 Joint Scoliosis Research Center of the Chinese University of Hong Kong and Nanjing University, Nanjing and Hong Kong, People’s Republic of China; 49 Department of Orthopaedics and Traumatology, The Chinese University of Hong Kong, Shatin, NT Hong Kong; 50 Joint Scoliosis Research Center of the Chinese University of Hong Kong and Nanjing University, Nanjing and Hong Kong, People’s Republic of China; 51 SH Ho Scoliosis Research Laboratory, The Chinese University of Hong Kong, Shatin, NT Hong Kong; 52 Spine Surgery, The Affiliated Drum Tower Hospital of Nanjing University Medical School, Nanjing, China; 53 Spine Surgery, the Affiliated Drum Tower Hospital of Nanjing University Medical School, Nanjing, China; 54 Department of Pediatric Orthopedics and Traumatology, University of Medical Sciences Poznan, Poznan, Poland; 55 Department of Cell Biology, University of Medical Sciences Poznan, Poznan, Poland; 56 Department of Medical Statistics, University of Warsaw, Warsaw, Poland; 57 Pusan National University School of Medicine, Busan, South Korea; 58 Department of Orthopaedics and Traumatology, The Chinese University of Hong Kong, Shatin, NT Hong Kong; 59 Joint Scoliosis Research Center of the Chinese University of Hong Kong and Nanjing University, Nanjing and Hong Kong, People’s Republic of China; 60 SH Ho Scoliosis Research Laboratory, The Chinese University of Hong Kong, Shatin, NT Hong Kong; 61 Spine Surgery, The Affiliated Drum Tower Hospital of Nanjing University Medical School, Nanjing, China; 62 Cincinnati Children’s Hospital Medical Center, Cincinnati, OH USA; 63 Polytechnique Montréal, Montréal, Canada; 64 Sainte-Justine University Hospital Center, Montréal, Canada; 65 Sonoran Spine Center and Research Foundation, Phoenix, AZ USA; 66 Mayo Clinic, Rochester, MN USA; 67 Department of Mechanical Engineering, University of Alberta, Edmonton, Canada; 68 Alberta Health Services, University of Alberta Hospital, Edmonton, Canada; 69 Division of Orthopaedic Surgery, University of Alberta, Edmonton, Canada; 70 Stollery Children’s Hospital, Edmonton, Alberta Canada; 71 IWK Health Centre, Halifax, Nova Scotia Canada; 72 Columbia University Medical Center/Morgan Stanley Children’s Hospital, New York, USA; 73 Harvard Medical School, Boston, USA; 74 Scoliosis SOS, London, UK; 75 Arkansas State University, Jonesboro, AR USA; 76 Independant, York, UK; 77 Columbia University Medical Center/Morgan Stanley Children’s Hospital, New York, NY USA; 78 Polytechnique Montréal, Montréal, Canada; 79 CHU Sainte-Justine, Montréal, Canada; 80 Université de Montréal, Montréal, Canada; 81 Hacettepe University, Ankara, Turkey; 82 University of Novi Sad, Faculty of Sport and Physical Education, Novi Sad, Serbia; 83 Scolio Centar, Novi Sad, Serbia; 84 Department of Pediatrics, University of Alberta, Edmonton, Canada; 85 MVZ Bad Sobernheim GmbH, Bad Sobernheim, Germany; 86 Clinique du parc, Lyon, France; 87 Lecante Group, Lyon, France; 88 Clinique du Parc, Lyon, France; 89 Lecante Group, Lyon, France; 90 U.O.C. of Orthopedics and Traumatology, Children’s Hospital Bambino Gesù, Institute of Scientific Research, P.zza S. Onofrio 4, Rome, 00165 Italy; 91 Department of Orthopedics, University Hospital “Agostino Gemelli”, Catholic University of the Sacred Heart School of Medicine, Rome, 00168 Italy; 92 Croix Rouge française-CMCR des Massues, Lyon, France; 93 Laboratoire de physiologie de l’exercice, Université de Lyon, Saint Étienne, France; 94 Hôpital Nord-Ouest, Villefranche sur Saône, France; 95 Teesside University/Peacocks Medical Group, Newcastle, UK; 96 Teesside University, Middlesbrough, UK; 97 ISICO, Milan, Italy; 98 Don Gnocchi Foundation, Brescia, Italy; 99 Brescia University, Brescia, Italy; 100 Clinique du parc, Lyon, France; 101 Lecante group, Lyon, France; 102 Spine Surgery, the Affiliated Drum Tower Hospital of Nanjing University Medical School, Nanjing, China; 103 Polytechnique Montréal, Montréal, Canada; 104 CHU Sainte-Justine, Montréal, Canada; 105 University of Montréal, Montréal, Canada; 106 The West Bengal University of Health Sciences & National Institute for the Orthopaedically Handicapped, Kolkata, India; 107 World Health Organization, Geneva, Switzerland; 108 Indian Statistical Institute, Kolkata, India; 109 Department of Physiotherapy and Rehabilitation, Faculty of Health Sciences, Hacettepe University, Ankara, Turkey; 110 School of Physical Therapy and Rehabilitation, Dokuzeylul University, Izmir, Turkey; 111 Department of Physiotherapy and Rehabilitation, School of Health Sciences, Dumlupinar University, Kutahya, Turkey; 112 Department of Audiology, Voice and Speech Disorders, Faculty of Health Sciences, Hacettepe University, Ankara, Turkey; 113 University of Alberta, Edmonton, Canada; 114 Alberta Health Services, Edmonton, Canada; 115 Interdisciplinary Division of Biomedical Engineering, The Hong Kong Polytechnic University, Hong Kong, China; 116 The University of Sydney, Sydney, Australia; 117 University of Alberta, Edmonton, Canada; 118 University of Calgary, Calgary, Canada; 119 Private Practice, Athens, Greece; 120 “Tzaneio” General Hospital of Piraeus, Piraeus, Greece; 121 University Hospital of Ioannina, Ioannina, Greece; 122 Nottingham University Hospitals Trust, Nottingham, UK; 123 Interdisciplinary Division of Biomedical Engineering, The Hong Kong Polytechnic University, Hong Kong, China; 124 Department of Rehabilitation Medicine, West China Hospital, Sichuan University, Chengdu, Sichuan China; 125 Institute for Disaster Management and Reconstruction, Sichuan University-Hong Kong Polytechnic University, Chengdu, Sichuan China; 126 Department of Rehabilitation Medicine, Wuxi Rehabilitation Hospital, Wuxi, Jiangsu China; 127 Department of Gastroenterology, The First Affiliated Hospital of Nanjing Medical University, Nanjing, Jiangsu China; 128 ISICO Italian Scientific Spine Institute, Milan, Italy; 129 University of Brescia, Brescia, Italy; 130 Don Gnocchi Foundation, Milan, Italy; 131 Columbia University Medical Center/Morgan Stanley Children’s Hospital, New York, NY USA; 132 Rady Children’s Hospital, San Diego, CA USA; 133 Children’s Hospital Colorado, Aurora, CO USA; 134 University of Rochester, Rochester, NY USA; 135 Children’s Hospital of Los Angeles, Los Angeles, CA USA; 136 University of Utah, Salt Lake City, UT USA; 137 Growing Spine Foundation, San Diego, CA USA; 138 Children’s Spine Foundation, Valley Forge, PA USA; 139 Department of Mechanical Engineering, Milwaukee School of Engineering, Milwaukee, WI USA; 140 Department of Orthopaedic Surgery, Medical College of Wisconsin, Milwaukee, WI USA; 141 University of Alberta, Edmonton, Canada; 142 Alberta Health Services, Edmonton, Canada; 143 Faculty of Health Sciences, Staffordshire University, Stoke-on-Trent, UK; 144 U.O.C. of Orthopedics and Traumatology, Children’s Hospital Bambino Gesù, Institute of Scientific Research, P.zza S. Onofrio 4, Rome, 00165 Italy; 145 Department of Orthopedics, University Hospital “Agostino Gemelli”, Catholic University of the Sacred Heart School of Medicine, Rome, 00168 Italy; 146 Independent practitioner, Rome, Italy; 147 Saitama Prefectural Rehabilitation Center, Ageo, Japan; 148 Saitama Medical Center, Saitama Medical University, Moroyama, Japan; 149 Spine Surgery, the Affiliated Drum Tower Hospital of Nanjing University Medical School, Nanjing, China; 150 Columbia University Medical Center/Morgan Stanley Children’s Hospital, New York, NY USA; 151 Scoliosis SOS, London, UK; 152 Independant, York, UK; 153 Department of Neurorehabilitation Sciences, Casa di Cura del Policlinico, Milan, Italy; 154 University of Milan, Milan, Italy; 155 ISICO Italian Scientific Spine Institute, Milan, Italy; 156 University of Bresciam, Brescia, Italy; 157 Don Gnocchi Foundation, Milan, Italy; 158 University of Alberta, Edmonton, Canada; 159 Alberta Health Services, Edmonton, Canada; 160 University of Alberta, Edmonton, Canada; 161 Alberta Health Services, Edmonton, Canada; 162 University of Alberta, Edmonton, Canada; 163 Alberta Health Services, Edmonton, Canada; 164 Scoliosis Spine Laser Centre, Athens, Greece; 165 Teesside University, Middlesbrough, UK; 166 Seoul Hyun Rehabilitation Hospital, Seoul, Korea; 167 Spine Academy Physical Therapy, PLLC, Lexington, MA USA; 168 University of Alberta, Edmonton, Canada; 169 Children’s Healthcare of Atlanta, Atlanta, GA USA; 170 University of Iowa, Iowa City, IA USA; 171 Northwestern University, Chicago, IL USA; 172 Ann & Robert H. Lurie Children’s Hospital of Chicago, Chicago, IL USA; 173 University of Alberta, Edmonton, Canada; 174 Alberta Health Services, Edmonton, Canada; 175 Children’s Hospital of Wisconsin, Medical College of Wisconsin, Milwaukee, WI USA; 176 Milwaukee School of Engineering, Milwaukee, WI USA; 177 Deptartment of Orthopaedic Surgery, Children’s Hospital of Wisconsin, Milwaukee, WI USA; 178 Medical College of Wisconsin, Milwaukee, WI USA; 179 Division of Biostatistics, Institute for Health and Society Education, University of Kentucky, Lexignton, KY USA; 180 The Spine Corporation, Millennium House, Foxwood Road, Chesterfield, S41 9RF UK; 181 James Cook University Hospitals, Marton Road, Middlesbrough, TS4 3BW UK; 182 ISICO Italian Scientific Spine Institute, Milan, Italy; 183 University of Brescia, Brescia, Italy; 184 Don Gnocchi Foundation, Milan, Italy; 185 Clinique du Parc, Lyon, France; 186 Lecante Group, Lyon, France; 187 Clinique du Parc, Lyon, France; 188 Lecante Group, Lyon, France; 189 Columbia University Medical Center/Morgan Stanley Children’s Hospital, New York, NY USA; 190 Université du Québec à Trois-Rivières, Trois-Rivières, Québec Canada; 191 Private Practice, Montréal, Canada; 192 Faculty of Medecine, University of Montreal, Montreal, Québec Canada; 193 Research Center, Sainte-Justine University Hospital Center, Montreal, Quebec Canada; 194 Murdoch University, School of Health Profession, Murdoch, WA Australia; 195 Interdisciplinary Division of Biomedical Engineering, The Hong Kong Polytechnic University, Hong Kong, China; 196 Moyarta 2, LLC, The Plains, VA USA; 197 National Scoliosis Center, Fairfax, VA USA; 198 Ottawa & District Physiotherapy Clinic and Scoliosis Physiotherapy and Posture Centre, Ottawa, Canada; 199 Saba University School of Medicine, Devens, MA USA; 200 Pediatric Rehabilitation New York (PRNY), Totowa, NJ USA; 201 Stolze Therapies, Denver, CO USA; 202 Department of Physical Therapy, Children’s Hospital of Wisconsin, Milwaukee, USA; 203 Department of Orthopaedic Surgery, Children’s Hospital of Wisconsin, Medical College of Wisconsin, Milwaukee, USA; 204 Morrison Physical Therapy, Lake Forest, Illinois USA; 205 Midwestern University, Downers Grove, Illinois USA; 206 Rosalind Franklin University, North Chicago, Illinois USA; 207 Faculty of Health Sciences, Staffordshire University, Stoke-on-Trent, ST4 2DF UK; 208 Université du Québec à Trois-Rivières, Trois-Rivières, Québec Canada; 209 Arkansas State University, Jonesboro, AR USA; 210 Private Practice, Montréal, Canada; 211 Faculty of Medecine, University of Montreal, Montreal, Québec Canada; 212 Research Center, Sainte-Justine University Hospital Center, Montreal, Quebec Canada; 213 Murdoch University, School of Health Profession, Murdoch, WA Australia; 214 U.O.C. of Orthopedics and Traumatology, Children’s Hospital Bambino Gesù, Institute of Scientific Research, P.zza S. Onofrio 4, Rome, 00165 Italy; 215 Department of Orthopedics, University Hospital “Agostino Gemelli”, Catholic University of the Sacred Heart School of Medicine, Rome, 00168 Italy; 216 Department of Orthopaedics and Traumatology, “Tzaneio” General Hospital of Piraeus, Piraeus, Greece; 217 Orthopaedic Surgeon, Athens, Greece; 218 Spine Disorders and Pediatric Orthopedics Department, University of Medical Sciences, Poznan, Poland; 219 Division of Virtual Engineering, University of Technology, Poznan, Poland; 220 Department of Biophysics, University of Medical Sciences, Poznan, Poland; 221 Laboratoire de Physiologie de l’Exercice (EA4338), Université Jean Monnet, Saint-Etienne, France; 222 Centre des Massues-Croix Rouge Française, 92 rue Edmond Locard, 69005 Lyon, France; 223 Ets Lecante, 125 rue Bataille, 69008 Lyon, France; 224 L’Hôpital Nord Ouest Villefranche/Saône, BP 436, 69655 Villefranche/Saône, France; 225 Group of Applied Research in Orthopedic (GARO), Villefranche/Saône, France; 226 Université de Montréal, Montréal, Canada; 227 CHU Sainte-Justine, Montréal, Canada; 228 Teesside University, Middlesbrough, UK; 229 University of Alberta, Edmonton, Canada; 230 Centre Médico-Chirurgical des Massues, Lyon, France; 231 Department of Spine Surgery, Drum Tower Hospital of Nanjing University Medical School, Nanjing, China; 232 Joint Scoliosis Research Center of the Chinese University of Hong Kong and Nanjing University, Hong Kong, China; 233 Department of Orthopedics and Traumatology, Chinese University of Hong Kong, Hong Kong, China; 234 Spine Surgery, the Affiliated Drum Tower Hospital of Nanjing University Medical School, Nanjing, China; 235 Spine Surgery, Drum Tower Hospital of Nanjing University Medical School, Nanjing, China; 236 The Joint Scoliosis Research Center of Nanjing University and the Chinese University of Hong Kong, Nanjing, China; 237 Department of Orthopaedics and Traumatology, The Chinese University of Hong Kong, Hong Kong, China; 238 ISICO (Italian Scientific Spine Institute), Milan, Italy; 239 Department of Clinical and Experimental Sciences, University of Brescia, Brescia, Italy; 240 IRCCS Fondazione Don Gnocchi, Milan, Italy; 241 Staffordshire University, Stoke-on-Trent, UK; 242 Department of Orthopaedic Surgery, University Medical Center Utrecht, Utrecht, The Netherlands; 243 Image Sciences Institute, University Medical Center Utrecht, Utrecht, The Netherlands; 244 Department of Imaging & Interventional Radiology, Prince of Wales Hospital, The Chinese University of Hong Kong, Hong Kong, China; 245 Department of Orthopaedics and Traumatology, Prince of Wales Hospital, The Chinese University of Hong Kong, Hong Kong, China; 246 Cincinnati Children’s Hospital Medical Center, Cincinnati, OH USA; 247 University of Cincinnati, Cincinnati, OH USA; 248 SpineForm LLC, Cincinnati, OH USA; 249 First Department of Orthopaedics, Athens University Medical School, ATTIKON University Hospital, Athens, Greece; 250 Department of Trauma and Orthopaedics “Tzaneion” General Hospital of Piraeus, Piraeus, Greece; 251 Department of Mechanical Engineering, University of Alberta, Edmonton, Canada; 252 Division of Orthopaedic Surgery, University of Alberta, Edmonton, Canada; 253 University of Alberta, Edmonton, Canada; 254 Pusan National University School of Medicine, Busan, South Korea; 255 Hamamatsu University School of Medicine, Hamamatsu, Japan

## O1 The reliability of the three dimensional spinal modeling of patients with adolescent idiopathic scoliosis using EOS system

### Aria Bagheri^1^, Xue-Cheng Liu^2,3^, Channing Tassone^2,3^, John Thometz^2,3^, Amie Chaloupka^2,3^, Sergey Tarima^1,4^

#### ^1^Division of Biostatistics, Medical College of Wisconsin Milwaukee, WI, USA; ^2^Department of Orthopaedic Surgery Milwaukee, WI, USA; ^3^Musculoskeletal Functional Assessment Center Milwaukee, WI, USA; ^4^Children's Hospital of Wisconsin Milwaukee, WI, USA

##### **Correspondence:** Xue-Cheng Liu

**Introduction**

Recently the EOS imaging system (EOS Imaging, Paris, France) has provided advancements in 3D spinal modeling. Advancements include low radiation as well as fast and accurate reconstructed measurements of spinal parameters. There is a paucity of studies analyzing the reproducibility of the EOS Imaging System and the sterEOS software in the production of 3D spinal models for children with adolescent idiopathic scoliosis (AIS).

**Objectives**

The purposes of the study were 1) to determine the intraclass correlation (ICC) for both the inter-observer and intra-observer in the measurements of Cobb angles in AP view as well as the Cobb angles in the lateral view; 2) to assess the ICC for inter- and intra-observer in the axial vertebral rotation (AVR) of the apex vertebra; 3) to compare differences of spinal parameters between two examiners and two trials; 4) to determine how long a 3D reconstruction of the spine takes.

**Methods**

Bilateral x-ray images of fifteen patients (age: 6 -15 years old, 5 males, 10 females) were retrospectively selected. These EOS images were uploaded into the sterEOS computer program. Within the software, spinal and pelvic parameters were identified manually to construct a 3D model of the spine. The sterEOS software calculates the Cobb angles, angles of lordosis, angles of kyphosis, and the AVRs of the apex vertebra. The 3D modeling was performed independently by two examiners. Each examiner modeled each patient's spine in two spaced out trials. The ICC between inter- and intra-observers were calculated and compared statistically.

**Results and discussion**

Both the inter- and intra-observers showed excellent reproducibility for the Cobb angles in the proximal segment (ICC: 0.72 - 0.91), kyphosis (ICC: 0.85- 0.92), and lordosis (ICC: 0.82 - 0.95). No significant differences were found between angle differences (0.35° to 2.4°). In contrast to the traditional radiography, the sterEOS provides a better high quality view within the sagittal plane. A moderate inter-observer ICC for the Cobb angle in the distal segment (ICC = 0.67) indicates the examiners have to carefully adjust the alignment and vertebrae in 3D rather than in 2D following the automatic computation from the EOS software. The inter-observer ICC for the AVR in the lumbar region (0.80) is higher than the thoracic or thoracolumbar region (0.65), but with high differences of AVR (4.0° - 6.3°). The average time that two examiners spent per subject ranged from 34.6 to 37.4 minutes.

**Conclusion and significance**

EOS provides significantly reliable and accurate spinal modeling in the measurement of children with AIS. Exposure to less radiation as compared to other radiographic modality allows EOS to offer acceptable quality view of the spine in the sagittal and transversal plane.

## O2 Classification and agreement of sagittal balance and compensatory mechanics in younger and older adults with scoliosis

### Larry Cohen, Milena Simic, Sarah Dennis, Kathryn Refshauge, Evangelos Pappas

#### The University of Sydney, Sydney, Australia

##### **Correspondence:** Larry Cohen

**Introduction**

Sagittal balance, a more important factor than coronal Cobb values, has been linked to back pain and quality of life. The SRS-Schwab adult spine deformity classification provides baseline values to evaluate sagittal balance and predictive equations to determine lumbopelvic compensatory patterns (LPCP). These equations are used to guide surgical decision making and technique selection. Although other lumbopelvic compensation equations are available, these have not been compared with the SRS-Schwab equation.

**Objectives**

The aim was to evaluate sagittal balance and LPCP in younger and older adults with scoliosis and to compare the two most commonly used LPCP predictive equations (SRS-Schwab and Legaye).

**Methods**

EOS radiographic data from 41 adults with scoliosis (coronal Cobb > 10°; 51 ± 19 years) stratified into younger (*n* = 20) and older (*n* = 21) groups above and below the mean age was retrospectively analysed. T-tests were used to compare group characteristics and Fisher’s exact tests were used to evaluate differences in SVA (sagittal vertical axis), PT (pelvic tilt) and PI (pelvic incidence)-LL (lumbar lordosis) mismatch thresholds. Agreement between SRS-Schwab and Legaye classification was evaluated using Kappa tests and Bland Altman plots.

**Results and discussion**

62% of the older group and 10% of the younger group exceeded the SVA threshold of 40 mm (*p* < .001). 86% of the older group and 20% of the younger group exceeded the 20° pelvic retroversion threshold (*p* < 0.001). Normal PI-LL mismatch ranges were more prevalent in the younger group (70%) than the older group (28%) (*p* < .001) when analysed through the SRS-Schwab equation. Legaye equation analysis revealed no difference in the prevalence of normal PI-LL ranges between the younger (15%) and older group (10%) (*P* = .66). Lumbar hyperlordosis was more prevalent in the younger (25%) than older group (5%) (*p* < .001) when analysed through the SRS-Schwab equation but no difference was observed between the younger (10%) and older group (0%) (*p* > .05) when analysed through the Legaye equation. Lumbar hypolordosis was more prevalent in the older (67%) than the younger group (5%) (*p* < .001) but no difference was observed between the older (90%) and younger group (75%) when analysed through the Legaye predictive equation (*P* = .33). Agreement between the SRS-Schwab and Legaye equations was poor for the whole (κ = 0.148), older (κ = 0.277) and young groups (κ = 0.039).

**Conclusion and significance**

This study confirms that older patients more often exhibit higher SVA and pelvic retroversion than younger patients. Whilst analysis through SRS-Schwab classification reveals that younger patients more often exhibit lumbar hyperlordosis than older patients who more often exhibit lumbar hypolordosis, analysis through the Legaye equations revealed no differences. There is poor agreement between the SRS-Schwab and Legaye classification equations. Clinicians are cautioned to exercise clinical judgement when evaluating their patients with these equations until more research is done.

## O3 Correlation between surface topography measurements and largest Cobb angle vary depending on adolescent idiopathic scoliosis curve type

### Eric C Parent^1^, Matthew Pietrosanu^1^, Emily Redford^1^, Sheri Schmidt^1^, Douglas Hill^1,2^, Marc Moreau^1,2^, Douglas Hedden^1,2^, Samer Adeeb^1^, Edmond Lou^1,2^

#### ^1^University of Alberta, Edmonton, Canada; ^2^Alberta Health Services, Edmonton, Canada

##### **Correspondence:** Eric C Parent

**Introduction**

Surface topography (ST) quantifies external deformity. The clinical standard to quantify scoliosis is the Cobb angle from radiograph. Convergent validity studies assessing ST measurements against this clinical standard resulted in variable correlation estimates. This variability may be related to curve types.

**Objectives**

The objective was to compare the correlations between ST measurements reflecting deformity in the frontal plane and the largest Cobb angles among subgroups with different curve types.

**Methods**

Volunteers (*N* = 263) with AIS, 14.3 ± 1.8 years old, with Cobb angle >10^o^, and treated non-operatively had a full-torso ST scan and posterior-anterior radiograph during the same visit to a scoliosis clinic. A frame was used for both imaging methods to reduce variability in positioning the extremities and body sway. The largest Cobb angle was measured on frontal radiographs using Image J blind to ST measurements. From ST, we extracted 15 frontal external deformity measurements using custom Matlab software: deviation of torso cross-section centroids, apparent asymmetry, trunk inclination, pelvic tilt, distance in half-area centroid location, decompensation, cosmetic score, POTSI, waistline (height index, depth index, area index), waist area index, angle between acromion line and lower corner of scapula line, ASIS-sternum angle, and angle between sternum and C7-S line. Curve types were determined by the apex location of curves >10^O^. Correlations between ST and Cobb measurements were obtained for the overall sample and each curve type subgroups (with *n* > 25): single thoracic (48), single thoracolumbar or lumbar (39), triple (31), and double (thoracic and lumbar 89). Correlations >0.5 are clinically important in convergent validity studies.

**Results and discussion**

Age (±1 yr) and BMI (±1.5 kg/cm^2^) were similar between curve types. However, mean largest Cobb angles differed as follows: overall 30 ± 14^O^, thoracic 27 ± 15^o^, thoracolumbar 21 ± 9^o^, triple 38 ± 13^O^, and double 35 ± 14^O^. The number of significant correlations differed among curve types: overall = 12, thoracic = 8, thoracolumbar = 8, triple = 6, double = 13. The correlations over the clinically important threshold varied by curve type: overall (*n* = 2: centroid deviation = 0.56, distance in half-area centroid = 0.67), thoracic (6: centroid deviation = 0.69, distance in half-area centroid = 0.79, POTSI = 0.59, waist area index = 0.61, waistline height = 0.62 and area = 0.60 indices), thoracolumbar (4: centroid deviation = 0.63, distance in half-area centroid = 0.60, waistline depth index = 0.52, waist area index = 0.64), triple (1: distance in half-area centroid) and double (3: centroid deviation = 0.64, lateral half-area centroid = 0.59, Waistline area index = 0.52).

**Conclusion and significance**

The severity of the external deformity in the frontal plane is related to the Cobb angle but correlations varied between curve types. Most clinically important correlations were observed in the thoracic curve subgroup where spinal deformity affects the surface via the ribs without compensation in other areas or by soft tissues. While some parameters performed better with specific curve types, the lateral asymmetry in the distance of the half-area centroids performed well in all subgroups.

## O4 Upright, prone and supine spinal morphology in adolescent idiopathic scoliosis

### Rob C. Brink^1^, Tom P.C. Schlösser^1^, Dino Colo^1^, Koen L. Vincken^2^, Marijn van Stralen^2^, Steve C.N. Hui^3^, Winnie C.W. Chu^3^, Jack C.Y. Cheng^4^, René M. Castelein^1^

#### ^1^Department of Orthopaedic Surgery, University Medical Center Utrecht, Utrcht, The Netherlands; ^2^Image Sciences Institute, University Medical Center Utrecht, Utrecht, The Netherlands; ^3^Department of Imaging & Interventional Radiology, Prince of Wales Hospital, The Chinese University of Hong Kong, Shatin, Hong Kong SAR; ^4^Department of Orthopaedics and Traumatology, Prince of Wales Hospital, The Chinese University of Hong Kong, Shatin, Hong Kong SAR

##### **Correspondence:** Rob C. Brink

“This abstract has not been included here as it has already been published.”

## O5 Trunkal changes in patients after total hip or knee arthroplasty: a surface topography study

### Vasileios Kechagias^1^, Theodoros B. Grivas^2^, Konstantinos Vlasis^3^, Konstantinos Michas^4^

#### ^1^Private practice, Athens, Greece; ^2^Department of Orthopaedics and Traumatology, Tzaneio General Hospital of Piraeus, Piraeus, Greece; ^3^Department of Anatomy, Medical School, University of Athens, Athens, Greece; ^4^Kimi Health Center, Kimi, Greece

##### **Correspondence:** Vasileios Kechagias

**Introduction**

Total hip or knee arthroplasty (THA/TKA) restores the hip or knee range of motion and alleviates the pain due to hip or knee arthritis. However, there is paucity of studies searching how THA or TKA affects the trunkal parameters. This is a surface topography study assessing THA or TKA affects on various spine and pelvis parameters.

**Objectives**

In this prospective study the short term effect of THA or TKA upon the trunkal morphological parameters in patients operated for severe hip or knee osteoarthritis is assessed, utilizing the surface topography method.

**Methods**

*Ethical issues:* This project was IRB approved and the patients consented. *The patients with THA:* Twenty-two patients, 12 women and 10 men, mean age 65.6 years, range 48-84 years, were assessed, preoperative, 4 months postoperative, while 9 of them (6 women and 3 men, mean age 65 years, range 52-76 years) were additionally assessed 12 months postoperative. *The patients with TKA:* Twenty-six patients were assessed, 20 women and 6 men, mean age 72.7 years, range 54-86 years, who were studied preoperative, 4 months postoperative, while 12 of them (9 women and 3 men, mean age 72.4 years, range 57-86 years) were additionally assessed 12 months postoperative. Five patients (3 women and 2 men, mean age 73.2 years, range 66-83 years) were assessed preoperative and only 12 months postoperative. *The apparatus and the studied parameters:* The sagittal and coronal imbalance, the kyphotic, lordotic and scoliosis angle, the apical deviation of the spinal column, the trunk inclination and imbalance, the pelvic obliquity and torsion were assessed. The Diers Formetric 4D analysis system was used for the measurements. *The statistical analysis* was performed using the IBM SPSS Statistics v.22.0 package. For the comparison between the patients preoperative, 4 months and 12 months postoperative the non-parametric statistical test Wilcoxon-rank was used. The variables are described as medians +/- interquartile range (75th to 25th percentile), due to the relative small size of the groups. All statistical tests had a significance level of *p* < 0.05.

**Results and discussion**

The analysis of the data showed statistically significant changes of the spine in sagittal plane after THA. The comparison of the 4 months’ postoperative data with the preoperative revealed decreased lordotic angle at 42 degrees from 47.1 degrees (*p* = 0.033). The comparison of the 12 months’ postoperative data with the same preoperatively revealed that the cervical-thoracic inflection point (the flexion point between cervical lordosis and thoracic kyphosis) changed at 7 mm from 5 mm (*p* = 0.035). The analysis of the data showed also statistically significant changes of the spine in sagittal plane after TKA. The 4 months post- and preoperative comparison revealed decreased lordotic angle at 49 from 50.8 degrees (*p* = 0.009). The 12 months post- to preoperative comparison revealed decreased lordotic angle at 50.1 from 51.7 degrees (*p* = 0.016). The analysis of the data did not show statistically significant changes of the pelvis after THA or TKA.

**Conclusions and significance**

The THA or TKA not only restores the hip or knee range of motion to a more normal condition and reduces the severe pain, but also contributes to a more correct patient posture and movement, by the restoration of the maximum lordotic angle of the spine to normal ranges.

## O6 Trunkal changes in patients suffering severe hip or knee osteoarthritis: a surface topography study

### Theodoros B. Grivas^1^, Vasileios Kechagias^2^, Konstantinos Vlasis^3^, Konstantinos Michas^4^

#### ^1^Department of Orthopaedics and Traumatology, Tzaneio General Hospital of Piraeus, Piraeus, Greece; ^2^Private Practice, Athens, Greece; ^3^Department of Anatomy, Medical School, University of Athens, Athens, Greece; ^4^Kimi Health Center, Kimi, Greece

##### **Correspondence:** Vasileios Kechagias

**Introduction**

It is known that patients with severe symptomatic hip or knee osteoarthritis (OA) have reduced hip range of motion. However, there is paucity of studies on the effect of hip or knee OA in various trunkal parameters. This is a surface topography study which evaluates how the hip or knee OA affects various spine and pelvis parameters.

**Objectives**

In this prospective study a) the effect of hip or knee OA upon the trunkal morphological parameters in patients suffering severe hip or knee OA is assessed and b) these parameters are compared with the equivalent parameters in a normal age matched control group, utilizing the surface topography method.

**Methods**

*Ethical issue:* This project was IRB approved and the patients consented. *The patients with hip OA:* Twenty-two patients, 12 women and 10 men with mean age 65.6 years, range 48-84 years, were assessed. *The patients with knee OA:* Thirty-one patients, 23 women and 8 men, mean age 72.8 years, range 54-86 years, were assessed. *The control group* (CG) consisted of 15 persons, 12 women and 3 men, with mean age 60.6 years, range 43-90 years. *The apparatus and the studied parameters:* The sagittal and the coronal imbalance, the kyphotic, lordotic and scoliosis angle, the apical deviation of the spinal column, the trunk inclination and imbalance, the pelvic obliquity and torsion were assessed. The Diers Formetric 4D analysis system was used for the measurements. *The statistical analysis* was performed using the IBM SPSS Statistics v.22.0. For the comparison between the group of patients and CG the non-parametric statistical test Mann-Whitney U was used. The variables are described as medians +/-interquartile range (75th to 25th percentile), due to the relative small size of the groups. All statistical tests had a significance level of *p* <0.05.

**Results and discussion**

The analysis of the data showed statistically significant changes of the spine in sagittal and transverse plane in patients with severe hip OA. In the sagittal plane the comparison with the CG revealed increased kyphotic apex at 197.8 mm compared to 178.6 mm in the CG, the fleche cervicale (the horizontal spatial distance from the kyphotic apex to the cervical apex) at 89.3 mm compared to 62.4 mm in the CG, the trunk inclination at 10.3 degrees compared to 3.5 degrees in the CG, the sagittal imbalance at 10 degrees compared to 3 degrees and at 66.5 mm compared to 27 mm in the CG. The fleche lombaire (the horizontal spatial distance from the kyphotic apex to the lordotic apex) was also reduced to 23 mm compared to 34.2 mm in the CG. In the transverse plane the surface rotation was 4.8 degrees compared to 3.2 degrees in the CG and the vertebral rotation was 5 degrees compared to 3 degrees in the CG. The analysis of the data showed statistically significant changes of the spine in sagittal plane in patients with severe knee OA. The comparison with the CG revealed increased fleche cervicale at 88.6 mm compared to 62.4 mm in the CG, the trunk inclination at 6.4 degrees compared to 3.5 degrees in the CG, the sagittal imbalance at 6 degrees compared to 3 degrees and at 42.7 mm compared to 27 mm in the CG. The analysis of the data did not show statistically significant changes of the pelvis in patients with severe hip or knee OA.

**Conclusions and significance**

The severe hip or knee OA in addition to reduced hip or knee range of motion, occasionally malalignment or contracture of knee, and severe pain which is affecting seriously the patients’ QoL, is also a cause of trunkal morphology alterations in sagittal and transverse planes as described above in the results section. The severe hip OA affects more extensively the trunkal morphological parameters as compared with the pertinent changes documented due to severe knee OA.

## O7 Assessing volumetric bone mineral density in adolescent idiopathic scoliosis: quantitative computed tomography vs high-resolution peripheral quantitative computed tomography: a pilot study

### Elisa M.S. Tam^1^, Fiona W.P. Yu^1,2^, Vivian W.Y. Hung^1,2^, Lin Shi^3^, Ling Qin^2^, Bobby K.W. Ng^1^, Winnie C.W. Chu^4^, James Griffith^4^, Jack C. Y. Cheng^1,2^, Tsz Ping Lam^1,2^

#### ^1^Department of Orthopaedics & Traumatology, The Chinese University of Hong Kong, Shatin, Hong Kong SAR; ^2^Bone Quality and Health Centre, Department of Orthopaedics & Traumatology, The Chinese University of Hong Kong, Shatin, Hong Kong SAR; ^3^Department of Medicine and Therapeutics, The Chinese University of Hong Kong, Shatin, Hong Kong SAR; ^4^Department of Imaging & Interventional Radiology, Prince of Wales Hospital, The Chinese University of Hong Kong, Shatin, Hong Kong SAR

##### **Correspondence:** Tsz Ping Lam

**Introduction**

Low bone mass has been reported in about 30% of girls with Adolescent Idiopathic Scoliosis (AIS). Quantitative Computed Tomography (QCT) could provide differential assessment of cortical and trabecular volumetric BMDs (vBMDs) which cannot be measured by areal DXA. However, regular assessment with QCT in adolescents is not appropriate due to the high radiation exposure. High-resolution Peripheral QCT (HR-pQCT) has been used as an alternative measurement for the axial vBMD.

**Objectives**

This pilot study aimed to test whether the vBMDs of distal radius measured with HR-pQCT correlate with that of lumbar spine from QCT in patients with AIS.

**Methods**

14 severe AIS girls aged 13-19 with pre-operative CT scan of the spine for planning of navigation surgery (Cobb angle^3^45¡) were recruited. Lumbar spine (L1-L4) and non-dominant distal radius were measured by QCT and HR-pQCT respectively. Results were analyzed with Pearson correlation.

**Results and discussion**

Of the 14 patients, 3 (21.4%) were osteopenic with z-score of femoral neck â‰¤-1. Significant correlations were found between L1, L2 vertebrae and distal radius including cortical vBMD (L1: *r* = 0.769, *p* = 0.026; L2: *r* = 0.550, *p* = 0.042) and trabecular vBMD (L1: *r* = 0.797, *p* = 0.018; L2: *r* = 0.645, *p* = 0.013). Correlations between L3 vertebra and distal radius were marginally significant (*p* < 0.1). The results of this study substantiated the use of a low radiation technique to measure the bone quality in our adolescent subjects.

**Conclusion and significance**

The vBMDs measured by QCT at L1 and L2 vertebrae (central skeleton) were correlated with that of HR-pQCT at non-dominant distal radius (peripheral skeleton) indicating that HR-pQCT, with very low radiation and capable of trabecular microarchitecture measurement, could be used for assessment for systemic bone quality. Further studies with larger sample size would be carried out to validate the findings.

## O8 Reduced white matter integrity at splenium of corpus callosum connecting to somatosensory cortex in AIS compared with normal control - a cerebral diffusion tensor imaging (DTI) study

### Cindy Xue^1^, Lin Shi^2^, Steve C. N. Hui^1^, Tsz Ping Lam^3^, Bobby K. W. Ng^3^, Jack C. Y. Cheng^3^, Winnie C. W. Chu^1^

#### ^1^Department of Imaging and Interventional Radiology, The Chinese University of Hong Kong, Shatin, Hong Kong SAR; ^2^Department of Medicine & Therapeutics, The Chinese University of Hong Kong, Shatin, Hong Kong SAR; ^3^Department of Orthopedics & Traumatology, The Chinese University of Hong Kong, Shatin, Hong Kong SAR

##### **Correspondence:** Winnie C. W. Chu

**Introduction**

There is increasing evidence for the possibility of an underlying neurological disorder for Adolescent Idiopathic Scoliosis (AIS). Abnormal somatosensory functions have been widely reported in AIS patients, including prolonged and asymmetrical latencies of SSEP, correlating with the side and progression. Poor postural balance control in AIS can be attributed to underlying abnormal somatosensory, visuo-oculomotor, or vestibular dysfunction or a combination of all the above factors as a result of ineffective central information processing. In this study, we examined the cerebral white matter fiber bundles in AIS using diffusion tensor imaging (DTI), in particular the corpus callosum (CC) as morphological changes in CC have been reported by our group previously.

**Objectives**

To investigate whether the white matter integrity within corpus callosum (CC) in AIS subjects differ from that in control subjects

**Methods**

Thirty-three right-handed AIS patients (female, right thoracic curve, mean age 14.9 ± 1.4) and thirty age-matched normal controls (NC) (female, mean age 14.7 ± 0.9) underwent DTI along 32 non-linear directions using a 3 T scanner. 3D segmentation of CC was performed semi - automatically using ITK-SNAP 2.4, and followed by regional segmentation (divided CC into genu, anterior & posterior body, isthmus and splenium) of the CC using the template defined by Witelson, et.al.

**Results and discussion**

The mean fractional anisotropy (FA) values of the genu and splenium of the CC interconnecting the somatosensory cortex were significantly lower in AIS patients than those in NC (*p* < 0.05); whereas the mid body and isthmus of the CC also showed a lower FA value in AIS though not yet reaching statistical significance. In both AIS and NC, there is a general trend of higher FA in all segments of CC on the left side (except genu), which probably related to right-handedness of all subjects. A significantly wider L-R difference was only observed in genu of CC between AIS and controls but not in other segments.

**Conclusion and significance**

Corpus callosum links the cerebral cortex of the left and right cerebral hemispheres and is the largest fiber pathway in the brain. Our finding of generalized reduced FA values in CC of AIS patients indicates that there is reduced white matter integrity of CC, in particular the CC fibers interconnecting the splenium and somatosensory cortex are preferentially involved. The findings are consistent with our clinical observation of significantly prolonged latency and increased latency difference on the side of major curve, where this incidence of SSEP abnormality occurred above the C5 level, which could be partially explained by the observed functional changes in CC fibers leading to ineffective signal transmission to the somatosensory cortex as well as possible impairment in interhemispheric transmission.

## O9 Radiation dose of digital radiography (DR) versus micro-dose x-ray (EOS) on patients with adolescent idiopathic scoliosis

### Steve C.N. Hui^1^, Jean-Philippe Pialasse^1,3^, Judy Y.H. Wong^1^, Tsz Ping Lam^2^, Bobby K.W. Ng^2^, Jack C.Y. Cheng^2^, Winnie C.W. Chu^1^

#### ^1^Department of Imaging and Interventional Radiology, The Chinese University of Hong Kong, Shatin, Hong Kong SAR; ^2^Department of Orthopedics & Traumatology, The Chinese University of Hong Kong, Shatin, Hong Kong SAR; ^3^Department of Chiropractic, University of Quebec at Trois-Rivieres, Quebec, Canada

##### **Correspondence:** Winnie C.W. Chu

**Introduction**

Patients with Adolescent Idiopathic Scoliosis (AIS) frequently receive x-ray imaging for diagnosis and follow-up monitoring purposes. The ionizing radiation exposure has accumulated through their development stage and the effect of radiation to this young vulnerable group of patients is uncertain. To achieve the ALARA (as low as reasonably achievable) concept of radiation dose in medical imaging, a new implementation of radiography, the biplanar digital x-ray by the EOS system, has been adopted.

**Objectives**

To investigate the radiation dose difference between using micro-dose biplanar x-ray and conventional digital radiography on patients with AIS.

**Methods**

Fifty-one subjects with AIS were recruited from the outpatient clinic at the university hospital. 37 of them (Mean age: 17.2 ± 2.1; BMI: 19.1 ± 2.4; Cobb: 31.5¡ ± 11.1¡) underwent micro-dose biplanar x-ray with the EOS system and 14 (Mean age: 16.9 ± 3.9; BMI: 19.9 ± 4.1; Cobb: 26.2¡ ± 10.5¡) underwent conventional digital radiography for whole spine images at posterior-anterior view. Entrance-skin dose was measured using thermoluminescent dosimeters (TLD) at three regions (i.e. sternal notch, nipple line, symphysis pubis). Effective dose and organ dose were calculated by simulation using PCXMC 2.0. Data from two x-ray systems were compared using independent two-sample T-test and significance level at 0.05.

**Results and discussion**

Entrance-skin dose measured at sternal notch, nipple line and symphysis pubis were 25.8 ± 4.9, 27.1 ± 5.1 and 28.6 ± 6.4 (μGy) respectively from micro-dose biplanar x-ray, and 151.9 ± 43.9, 539.7 ± 204.9 and 771.2 ± 267.2 (μGy) respectively from conventional digital radiography. Entrance-skin dose from the micro-dose biplanar x-ray system was 5.9 - 27.0 times lower at various regions compared with conventional digital radiography. The calculated effective doses were 2.8 ± 0.5 (μSv) and 65.2 ± 18.5 (μSv) from micro-dose biplanar system and conventional digital radiography respectively. Patients with AIS underwent micro dose biplanar x-ray system received approximately 23 times less effective dose than those underwent conventional digital radiography. The organ dose at thyroid, lung and ovaries/testes regions were 0.84 ± 0.4, 5.8 ± 1.0, 2.4 ± 0.8 (μSv) respectively from the biplanar x-ray and 13.4 ± 2.7, 105.3 ± 33.9, 78.0 ± 30.2 (μSv) respectively from the conventional digital radiography. Patients with AIS underwent micro-dose biplanar x-ray received 16-31 times less organ dose compared to those underwent conventional digital radiography.

**Conclusion and significance**

Entrance-skin dose, effective dose and organ dose were statistically significantly lower in micro-dose biplanar x-ray compared with conventional digital radiography. As AIS patients require periodic x-ray follow up for surveillance of curve progression, clinical use of micro-dose bi-planar system is beneficial for these young patients to reduce the intake of ionizing radiation.

## O10 Prediction of scoliosis progression using three-dimensional ultrasound images: a pilot study

### Quang N. Vo, Lawrence H. Le, Edmond H. M. Lou

#### University of Alberta, Edmonton, Canada

##### **Correspondence:** Edmond H. M. Lou

**Introduction**

Scoliosis is a complicated three-dimensional spinal deformity. Currently, the Cobb angle, measured on the postero-anterior (PA) radiograph, is the gold standard to assess the severity of scoliosis. The PA Cobb angle does not reflect the true deformity, but it has been used as an indicator to estimate the progression of scoliosis. Our group has developed a three-dimensional ultrasound method to scan children with scoliosis and reconstruct the plane of maximum curvature (PMC) from which the true Cobb angle (CApmc) can be estimated. In addition, the axial vertebral rotation (AVR) and the lateral deviation (LDpmc) of each curve on the PMC can be measured.

**Objectives**

This study was to develop a model to predict the CApmc based on previous ultrasound measured parameters.

**Methods**

Thirty-two patients who were diagnosed with adolescent idiopathic scoliosis and had at least two consecutive clinic visits with ultrasound scans were recruited. Twenty-six datasets were used to build the prediction model while the other 6 were used for preliminary validation. The six parameters used for the prediction model included the CApmc, AVR, LDpmc, the number of curves (NC), and torsion of the first visit, and elapsed time between the two visits. Meanwhile, the CApmc of the second visit was the output of the prediction. The establishment of the prediction model was based on the linear regression. Each predictor and their combinations were analyzed to find the correlation with the output. The statistical Pearson coefficient (R-value) and mean absolute difference (MAD) were used to assess the results.

**Results and discussion**

The R-values of individual predictors were 0.890, 0.382, 0.423, 0.072, 0.289, and 0.130 for the CApmc, AVR, LDpmc, NC, torsion, and elapsed time, respectively. Even though the number of curves and elapsed time had individually insignificant influence on the prediction, the combination of all six predictors yielded the greatest multiple R-value (0.925) and smallest standard error (4.015) of the final prediction model: y = 12.25696 + 0.93794*x1 - 0.36834*x2 - 0.07740*x3 - 1.74132*x4 + 3.95618*x5 - 0.02777*x6 where: - x1: The nth-visit Cobb angle on the plane of maximum curvature, - x2: The axial apical vertebral rotation, - x3: The lateral deviation on the plane of maximum curvature, - x4: The number of curves, - x5: The torsion (= apical rotation/the number of vertebrae within the curve), - x6: The elapsed time between the two visits, - y: The (n + 1)th-visit Cobb angle on the plane of maximum curvature. The result also showed a high correlation between the predicted and measured Cobb angle with R-value = 0.811 and MAD = (3.8 ± 0.7) degrees. Among six validated cases, only one case showed a decrease in the measured Cobb angle while the corresponding predicted value remained unchanged.

**Conclusion and significance**

This preliminary model presented a potential for estimating curve progression. Further study involving more patients should be done to validate the model.

## O11 Validity of using ultrasound imaging method to detect curve progression for adolescent idiopathic scoliosis (AIS)

### Rui Zheng^1^, Douglas L. Hill^1,2^, Marc J. Moreau^1^, Douglas M. Hedden^1^, James K. Mahood^1^, Sarah Southon^1^, Edmond Lou^1,2^

#### ^1^University of Alberta, Edmonton, Canada; ^2^Alberta Health Services, Edmonton, Canada

##### **Correspondence:** Edmond Lou

**Introduction**

Ultrasound (US) imaging with the aid of previous radiographs (AOR) has been shown to reliably and accurately measure coronal curvatures in children with AIS. Over 109 measurements, the intra-class correlation coefficients (ICC) of intra- and inter-rater reliabilities of the US Cobb measurement were 0.95 and 0.91, respectively. The correlation (R2) and the mean absolute difference ± standard deviation (MAD ± SD) between the US and the radiographic measurements were 0.90 and 2.8 ± 2.2°, respectively. However, the validity of detecting curve progression using US has not been reported.

**Objectives**

To determine the sensitivity, specificity, positive predictive value (PPV) and negative predictive value (NPV) of the proxy Cobb angle in children with AIS using US imaging method.

**Methods**

Sixty-nine AIS subjects (60 F, 9 M; 14.6 ± 1.8 years; Cobb angle range 11 - 45o) who had no brace or surgical treatment during this study participated. All subjects had baseline radiographs (X0), first follow-up radiograph and US scan (X1, US1), and second follow-up radiograph and US scan (X2, US2). The range of time between the initial to the first visit and the first visit to the second is between 2 to 13 months. A rater with 3 years of ultrasound measurement experience measured the US proxy Cobb using the AOR method. The Cobb values measured during the scoliosis clinic and recorded in the electronic record from both X1 and X2 were used as the reference measurements and to determine the curve progression (Cobb change ≥ 5o). The sensitivity, specificity, PPV and NPV of the progression comparing (1) US2 vs X1, and (2) US2 vs US1 were reported.

**Results and discussion**

The MAD ± SD of the Cobb angle between US1 vs X1 and US2 vs X2 were 2.5 ± 1.7° and 2.1 ± 1.6°, respectively. Fifteen subjects had curves that progressed based on the clinical records. By comparing the second ultrasound (US2) with the first radiograph (X1), the sensitivity, specificity, PPV, NPV and accuracy were 12/15(80%), 52/54(96%), 12/14(86%), 52/55(95%), and 64/69(93%) respectively. Two false negatives were with curves that changed from 12 to 17o and one false negative occurred in a 19o curve with a standing posture found different from the images. By comparing the two US images (US2 vs US1), the sensitivity, specificity, PPV, NPV and accuracy were 10/15(67%), 46/54(85%), 10/18(56%), 46/51(90%) and 56/69(81%), respectively. Both false negatives and false positives were increased because the measurement errors increased by comparing 2 US measurements with 2 radiographic measurements (ultrasound measurement aided by previous radiograph).

**Conclusion and significance**

The imaging modalities of radiograph and US are different. Although the measurement error is similar, radiographs cannot be completely replaced with ultrasound. However, ultrasound has sufficient validity to be used in every 2nd clinic visit (US2 vs X1), reducing ionizing radiation exposure by up to 50%.

## O12 Automatic extraction of vertebral landmarks from ultrasound images

### Arnaud Brignol^1^, Farida Cheriet^2,3^, Marie-Claude Miron^3^, Catherine Laporte^1^

#### ^1^École de technologie supérieure, Montréal, Canada; ^2^Polytechnique Montréal, Montréal, Canada; ^3^CHU Sainte-Justine, Montréal, Québec

##### **Correspondence:** Arnaud Brignol

**Introduction**

3D ultrasound imaging is emerging as a promising non invasive modality for assessing adolescent idiopathic scoliosis. Using ultrasound images is challenging because of the low quality of the data. Quantitative assessment of the scoliotic deformity from these images currently relies primarily on manual landmark identification, which is time-consuming and prone to low repeatability.

**Objectives**

To address this difficulty, the objective of this study is to propose a fast, repeatable and automatic method to extract the spinous process and the two laminae from vertebral ultrasound images.

**Methods**

The extraction of the landmarks was accomplished by exploiting the appearance of the bone surfaces (bright intensity and shadow) in ultrasound images. We defined a feature map of an image wherein each position (x, y) takes a value equal to the ratio between the sum of the intensities of the pixels of the same row x and the sum of the intensities of the pixels of the same column y in the ultrasound image. Local maxima in this map are used to find the position of the spinous process and the laminae. The method was tested on vertebral ultrasound images (*n* = 30) acquired in the transverse plane with a linear probe (scanning depth of 4.5-6 cm and central frequency set to 9 MHz) for two healthy subjects lying on a stretcher in prone position. The spinous process and the laminae were then manually identified by four observers and automatically located with our method.

**Results and discussion**

The inter-observer variability between the four observers is 1.62+/-0.64 mm while the average distance between the results obtained by the proposed method and each observer is 1.71+/-0.62 mm. A paired t-test showed no significant difference between inter-observer variability and the average distance between the automatic and manual identification (*p* = 0.35). Automatic extraction of landmarks in one image requires 100 ms. Furthermore, automatic detection of the landmarks is repeatable and will lead to reproducible computed vertebra orientation.

**Conclusion and significance**

Our preliminary results show that the method has the potential to extract vertebral landmarks in a repeatable way with accuracy on the same order as the inter-observer variability of manual identification. Further work is under way to improve the precision by defining better criteria for locating the laminae. Once validated, the proposed method will improve the computation of the coronal curvature from the center of laminae method and will allow reliable clinical assessment of scoliosis from ultrasound images.

## O13 Does disc wedging contribute to the flexibility of thoracic curve in adolescent idiopathic scoliosis?

### Yong Qiu, Hao Liu, Zhen Liu, Ze-zhang Zhu, Bang-ping Qian

#### The Department of Spine Surgery, the Affiliated Drum Tower Hospital of Nanjing University Medical School, Nanjing, China

##### **Correspondence:** Yong Qiu

“This abstract has not been included here as it has already been published.”

## O14 Impacts of different torques on remodeling of the caudal vertebral growth plate

### XueCheng Liu^1^, Robert Rizza^2^, John Thometz^1^, Derek Rosol^1^, Channing Tassone^1^, Sergey Tarima^3^, Paula North^4^

#### ^1^Department of Orthopaedic Surgery, Medical College of Wisconsin, Milwaukee, WI, USA; ^2^Department of Mechanical Engineering, Milwaukee School of Engineering, Milwaukee, WI, USA; ^3^Division of Biostatistics, Medical College of Wisconsin, Milwaukee, WI, USA; ^4^Department of Pathology, Children's Hospital of Wisconsin, Medical College of Wisconsin, Milwaukee, WI, USA

##### **Correspondence:** XueCheng Liu

**Introduction**

Numerous studies indicate that longitudinal loading affects the vertebral growth plate and disc leading to vertebral wedging deformation. Torsional loading impacts disc gene expression and annular stiffness. However, its effect on chondrocytes in the growth plate (longitudinal and circumferential growth) has not been investigated, let alone the comparisons between static loading and dynamic loading in the asymmetric remodeling of the caudal vertebra.

**Objectives**

The aims of the study were: 1) to assess the bony morphological changes in radiography under no loading, static and dynamic torque; 2) to investigate the microstructural changes of the growth plate in histology; 3) to determine the changes in the expression of Proliferating cell nuclear antigen (PCNA) in the growth plate following the static and dynamic loading.

**Methods**

Eighteen Sprague-Dawley female rats were randomly divided into three groups: The Static Torque Group (ST) had six rats that received a static torque (1.25 Nm) between adjacent vertebral bodies of the rat tail (functional spine unit); the Dynamic Torque Group (DT) had six rats and received a dynamic loading (2.4 Nm at 1.0 Hz for 15 minutes/time, 3 times/week). The DT group was exposed to dynamic torque application with 20.5° rotation by a specifically designed solenoid device while being held in a tubular restraint. The Sham Control Group (SC) composed of six rats having no applied torque. All the rats were sacrificed after 4 weeks and prepared for histological analysis and immunocytochemistry. ANOVA, ANCOVA, and Post hoc testing were performed.

**Results and discussion**

There were no morphologic changes on the caudal vertebrae in the rat tail on X-ray. There were significant differences of right side disc height and average disc height on the proximal vertebrae space in the coronal plane of the X-ray among three groups. Although there were no significant differences of the physeal height between the ST and DT, there were significant differences of the physeal height between ST (0.17 ± 0.02 mm) and SC (0.21 ± 0.03 mm), or between DT (0.16 ± 0.03 mm) and SC (0.21 ± 0.03 mm) (*p* < 0.05). The PCNA were only detected in the reserve zone for the DT with 50% samples. The PCNA were found in the proliferative zone for both the ST and DT with 50% samples as compared to the SC with 17% samples. However, there was no difference of PCNA incidence between the ST and DT. In the hypertrophic zone in adjacent to the calcified area, the PCNA were apparently presented in the three groups (100% in the SC, 67% in the ST, and 50% in DT).

**Conclusion and significance**

This study may indicate that both dynamic and static torsional loading alters the incidence and location of cell proliferation and results in an abnormal growth. The mechanically modulation growth responds to different torques may help us comprehend the development and treatment of scoliosis.

## O15 Anorexia nervosa: a risk factor for scoliosis? Insights from a cross sectional study

### Fabio Zaina^1^, Francesca Pesenti^2^, Stefano Negrini^3,4^, Luca Persani^2^, Paolo Capodaglio^5^, Nicoletta Polli^2^

#### ^1^ISICO, Milan, Italy; ^2^Division of Endocrine & Metabolic Disease, Istituto Auxologico Italiano, Milan, Italy; ^3^Don Gnocchi Foundation, Brescia, Italy; ^4^Brescia University, Brescia, Italy; ^5^Rehabilitation Department, Istituto Auxologico Italiano, Verbania, Italy

##### **Correspondence:** Fabio Zaina

**Introduction**

A long debate exists about the possible role of anorexia in the genesis of scoliosis. Usually adolescent scoliosis patients have a reduced BMI with respect to matched age girls and this gave origin to a speculation about a possible correlation of these too pathologies. Despite different findings, all the studies about this topic have been performed in populations of scoliosis patients where researchers looked for eating disorders, but no study up to date ever evaluated the prevalence of scoliosis in anorexia nervosa patients.

**Objectives**

The aim of the study is to evaluate the prevalence of idiopathic scoliosis in patients affected by anorexia nervosa.

**Methods**

Design: cross-sectional study. Study group: convenience sample of all the available patients matching the inclusion criteria that attended the facility both as in- and out-patients from June 2013 to November 2014. Control Group: female subjects coming from an epidemiological screening for scoliosis performed in secondary schools. Inclusion criteria: diagnosis of anorexia nervosa according to DSM-IV-TR criteria. To be included patients must have developed anorexia during adolescence (age considered 11-18 yrs) and their body mass index (BMI) have been ≤ 17.5 kg/m^2^ for at least 6 months. Protocol: We applied a two-level screening, based on a first evaluation performed by a trained medicine student and then a second one by a scoliosis treatment expert. The participants were first screened using a Bunnell scoliometer being considered positive if resulted to have ≥5° Bunnell degrees and thus referred to the second level. Those who had a previous diagnosis of scoliosis were referred for the second level. We calculated also the odds ratio comparing the data with the Italian normative data about scoliosis prevalence coming from a large epidemiological study including more than 16,000 subjects.

**Results and discussion**

Seventy-seven out of 246 eligible patients affected by anorexia nervosa entered the study, while the control group consisted of 816 females screened for scoliosis during secondary of high school. The prevalence of scoliosis in Anorexia group was 16.9%, with an odds ratio of 5.77 (CI95 3.12-10-67) with respect to the control group, and 6.25 (CI95 5.70-6.79) with respect to literature data. This analysis is a best-case scenario, since we considered the drop-out as non affected by scoliosis. If we consider a worst-case scenario, with all the drop-out affected by scoliosis, the prevalence would be 28.5%.

**Conclusion and significance**

This is the first study performed in anorexia nervosa patients and it has shown that they have a 6-fold greater risk of presenting with idiopathic scoliosis. A cause-effect relationship cannot be determined due to the design of the study, but further prospective studies should be performed in order to better define the nature of this relationship and common biochemical pathways.

## O16 A longitudinal cohort of 513 patients - can bone mineral density (BMD) predict the curve progression and risk of surgery in newly diagnosed girls with adolescent idiopathic scoliosis (AIS)?

### Benjamin Hon Kei Yip^1,2^, Fiona Wai Ping Yu^1,3^, Vivian Wing Yin Hung^1,3^, Tsz Ping Lam^1,3^, Ling Qin^1^, Bobby Kin Wah Ng^1^, Jack Chun Yiu Cheng^1,3^

#### ^1^Department of Orthopaedics and Traumatology, Faculty of Medicine, Prince of Wales Hospital, The Chinese University of Hong Kong, Shatin, Hong Kong SAR; ^2^School of Public Health and Primary Care, Faculty of Medicine, The Chinese University of Hong Kong, Shatin, Hong Kong SAR; ^3^Joint Scoliosis Research Center of the Chinese University of Hong Kong and Nanjing University, Nanjing and Hong Kong, People’s Republic of China

##### **Correspondence:** Jack Chun Yiu Cheng

**Introduction**

Adolescent Idiopathic Scoliosis (AIS) is a three-dimensional spinal deformity with lateral curvature of > 10 degree measured by Cobb Angle. Osteopenia was found to occur in ~30% of AIS patients that can persist beyond skeletal maturity and recognized as a prognostic factor for curve progression. No previous study has been conducted to assess the predictive ability of BMD on curve progression in terms of Cobb angle reaching surgical threshold of 45 degrees or having gone through surgery.

**Objectives**

The objective of this study was to investigate the incremental prognostic value of osteopenia at initiative clinical visit on surgical outcomes through longitudinal follow up.

**Methods**

Between 1995-2014, 513 AIS girls with Cobb angle ^3^10¡ but ^2^40¡ (i.e. had not yet reached the study outcome) without prior treatment were recruited and followed from their first visit till skeletal maturity defined as age ^3^15.5 years and ^3^2 years post-menarchal with an average follow-up time of 5.1 years (SD = 2.6). Bilateral hips were measured by DXA at first clinic visit, followed by regular follow-up clinical & radiological assessments. The study outcome was indication of need for surgery defined as Cobb angle^3^45Â° or actually had undergone surgery. Statistically, Akaike Information Criterion (AIC) was used to evaluate incremental prognostic value of osteopenia status (OST) when compared to conventional model.

**Results and discussion**

At first clinical visit, the mean age was 13.1 years (SD = 1.1), with an average Cobb angle of 25¡ (SD = 5.7), and an average of the z-score of BMD of -0.4 (SD = 0.7). Among 513 subjects, 55 progressed to Cobb angle^3^45¡ or went through surgery. The proportions of subjects with osteopenia having the need for or actually having gone through surgery versus that without osteopenia were 17.2% and 7.6% respectively. Cox proportional hazard model with osteopenia status had better overall performance than model without (AIC533.13 vs 528.56) and improved the data fitness significantly (*p* = 0.0104). AIS patients with osteopenia had significantly higher risk (HR2.24, *p* = 0.011) of indication of surgery, while lower baseline Cobb angle (HR1.15), menarche status (HR0.414) and older baseline age (HR0.532) were significantly associated with lesser risk of indication of needs for surgery.

**Conclusion and significance**

In conclusion, results suggested that AIS patients with osteopenia status would have significantly higher risk of deterioration to the surgical level. Early evaluation of osteopenia status that could reflect the bone quality and mechanical bone strength might with further validations be an important additional investigation in predicting the risk of curve progression and subsequent need for surgery at the initial presentation. This could have important potential clinical implications in helping with the prognostication and bracing treatment decision.

## O17 Dysfunctional osteogenic and osteocytic activity in adolescent idiopathic scoliosis (AIS)

### Jiajun Zhang^1,2,3^, Wayne Yuk Wai Lee^1,2,3^, Huanxiong Chen^1,2,3^, Elisa Man Shan Tam^1,2,3^, Gene Chiwai Man^1,2,3^, Tsz Ping Lam^1,2,3^, Bobby Kin Wah Ng^1,2,3^, Yong Qiu^2,3,4^, Jack Chun Yiu Cheng^1,2,3^

#### ^1^Department of Orthopaedics and Traumatology, The Chinese University of Hong Kong, Shatin, N.T., Hong Kong; ^2^Joint Scoliosis Research Center of the Chinese University of Hong Kong and Nanjing University, Nanjing and Hong Kong, People’s Republic of China; ^3^SH Ho Scoliosis Research Laboratory, The Chinese University of Hong Kong, Shatin, NT, Hong Kong; ^4^Spine Surgery, The Affiliated Drum Tower Hospital of Nanjing University Medical School, Nanjing, China

##### **Correspondence:** Jack Chun Yiu Cheng

**Introduction**

Bone quality of Adolescent Idiopathic Scoliosis (AIS) has been characterized with lower bone mineral density, deranged bone microstructure, and abnormal ultrastructure of osteocytes and lacuno-canalicular network. In addition to abnormal primary osteoblasts activities, AIS was also reported to exhibit unique bone metabolite profile including higher serum RANKL/OPG ratio, higher serum osteocalcin and bone specific alkaline phosphatase. Osteoblasts and their descendent osteocytes orchestrate bone metabolism and quality via cell to cell communication and paracrine/endocrine signaling. We hypothesized that the observed pathologic phenomenon of AIS might be attributed to impaired osteogenic and osteocytic activity.

**Objectives**

To compare the osteogenic and osteocytic activities between AIS and non-AIS controls in vitro.

**Methods**

In this case-control study, primary osteoblasts were isolated from trabecular iliac crest bone biopsies intraoperatively from AIS and non-AIS control subjects. Osteoblast cultured in two-dimensional (2D) monolayer condition with standard osteogenic medium and 3D condition embedded within 2 mg/ml collagen type I gel were established to determine osteogenic and osteocytic activity, respectively. Osteogenic activity was determiend with Alamar blue for cell metabolic activity, alkaline phosphatase (ALP) staining and Alizarin Red staining (ARS) for mineralization capacity; qPCR for mRNA expression of representative osteogenic markers, and ELISA test for RANKL and OPG. While osteocytic activity was determined with osteocytes specific markers at mRNA, intracellular protein and secreted protein levels by qPCR, Western blot and ELISA, respectively.

**Results and discussion**

Alamar blue result showed AIS and control osteoblasts exhibiting similar metabolic activity. AIS osteoblasts produced less calcium deposits (ARS) after 14 days of culture (*p* = 0.021). qPCR study revealed higher mRNA level of collagen I (p = 0.018) and, but lower level of Osteopontin (*p* = 0.02), E11 (canaliculae formation marker) (*p* = 0.018) and OPG (*p* = 0.013) in AIS osteoblasts. AIS osteoblasts secreted less OPG (*p* = 0.011), resulting in higher RANKL/OPG ratio and numerically more number of TRAP positive multinuclear osteoclasts in primary mononuclear cells culture. AIS osteocytes differentiated from osteoblasts in 3D culture had lower mRNA expression of sclerostin (*p* = 0.021) and FGF23 (*p* = 0.02). ELISA assay showed the level of secreted sclerostin in 3D culture reduced by 14.8 fold in AIS.

**Conclusion and significance**

This study showed abnormal osteogenic and osteocytic activities in AIS, suggesting decreased mineralization ability, impaired extracellular matrix and canaliculae formation, decreased bone resorption inhibition and altered osteocyte activity which might contribute to the observed unique bone quality and metabolism in AIS. This study bridges the etiology gap of observed pathologic phenomenon with cellular level understanding.

**Acknowledgement**

This project is supported by Hong Kong RCG (463113), and CUHK Direct grant (4054190).

## O18 Mechanism of abnormal bone density and quality in adolescent idiopathic scoliosis: experimental scoliosis in bipedal C57BL/6 J mice

### Hao Liu, Zhen Liu, Zezhang Zhu, Bang Ping Qian, Yong Qiu

#### Spine Surgery, the Affiliated Drum Tower Hospital of Nanjing University Medical School, Nanjing, China

##### **Correspondence:** Yong Qiu

**Introduction**

The effect of melatonin on the bone density and quality status in adolescent idiopathic scoliosis (AIS) has not been identified.

**Objectives**

To clarify the effect of melatonin on the bone density and quality status in AIS, we investigated radiological and histological changes in the C57BL/6 J mice model.

**Methods**

A total of 80 mice were randomly divided into four equal groups: 20 quadrupedal mice served as controls (QP); 20 bipedal mice (BP); the remaining 20 quadrupedal (QP + MLT) and 20 bipedal mice (BP + MLT) received daily intraperitoneal administration of melatonin (8 mg/kg body weight in 10% ethanol/saline) at 22:00. Bipedal mice will be amputated at 3 weeks old under general anesthesia. The bipedal mice without a tail were able to walk with standing posture, whereas the quadrupedal mice did not walk with standing posture. The spines of all mice will be radiologically examined for the presence of scoliosis and micro-computed tomography (micro-CT) images will be used for the three-dimensional assessment of bone density and quality every 4 weeks. The following parameters will be calculated: trabecular bone vBMD, bone volume fraction (BV/TV), trabecular number (Tb.N), trabecular thickness (Tb.Th), trabecular separation (Tb.Sp). Before killing, blood samples were collected in the middle of dark cycle and melatonin levels were measured by radioimmunoassay. Histological specimens of the scanned lumbar vertebra were prepared, and a mid-sagittal section was stained with hematoxylin and eosin and tartrate-resistant acid phosphatase to evaluate the numbers of osteoblasts and osteoclasts, respectively.

**Results and discussion**

Scoliosis with rib humps developed in 16 of 20 bipedal mice and in six quadrupedal mice. In contrast, only 4 mice in the BP + MLT group and one in the QP + MLT group developed scoliosis. In BP and QP group, the serum melatonin was reduced to nearly zero; however, the normal level was restored in both BP + MLT and QP + MLT group. Micro-CT data revealed that mean value of BMD in BP + MLT groups were significantly higher at the vertebral body in mice compared with BP group (*P* < 0.001). BV/TV and Tb.N in the BP + MLT group was significantly higher than that in the BP group (*P* < 0.001), while Tb.Sp in the BP + MLT group was significantly lower than that in BP (*P* < 0.001). The number of osteoblasts was significantly increased in the BP + MLT group, compared with BP group and QP group (*P* < 0.001).

**Conclusion and significance**

Our results suggest that bipedal is critical in the development of scoliosis. Melatonin deficiency in bipedal mice appears to play crucial role for the development of scoliosis, and restoration of melatonin levels prevents the development of scoliotic deformity, low bone density and abnormal bone quality.

## O19 A replication study for association of TIMP-2 gene polymorphism with curve progression and initiation of right thoracic idiopathic scoliosis in caucasian population

### P. Harasymczuk^1^, M. Andrusiewicz^2^, P. Janusz^1^, P. Biecek^3^, T. Kotwicki^1^, M. Kotwicka^2^

#### ^1^Department of Pediatric Orthopedics and Traumatology, University of Medical Sciences Poznan, Poznan, Poland; ^2^Department of Cell Biology, University of Medical Sciences Poznan, Poznan, Poland; ^3^Department of Medical Statistics, University of Warsaw, Warsaw, Poland

##### **Correspondence:** P. Harasymczuk

**Introduction**

Idiopathic scoliosis (IS) is a common spinal deformity and has a strong genetic predisposition. Predicting curve progression is important in clinical practice. The initiation and progression of IS is reported to be associated with a number of genes. Associations with tissue inhibitor of metalloproteinase 2 (TIMP-2) have been reported in Han Chinese with IS, but not confirmed in Japanese population; however, there has been no replication study for it in Caucasian subjects. A genetic association study of rs8179090 single nucleotide polymorphism (SNP) of TIMP-2 gene promoter was previously reported to be associated with curve progression of idiopathic scoliosis (IS).

**Objective**

To determine whether the associations of rs8179090 with curve progression or initiation reported in Chinese population are replicated in Caucasian patients with IS in homogenous group based on curve pattern and clinically important curve progression criteria.

**Methods**

As a part of a larger TIMP-2 tagSNP study we recruited 100 girls with IS with a Cobb angle of 20° or greater of right thoracic scoliosis and 100 healthy girls. Study design power of 87%, based on expected OR of 3,0 and disease prevalence of 0,03 was calculated by Association Design Study Server. Scoliosis patients were grouped according to their peak annual progression rate into progression (>12 deg/year) and nonprogression groups(<12 deg/year). To replicate the studies conducted in Chinese and Japanese populations genotype analyses of rs8179090 were performed using allele specific PCR based hybridizing FRET probes designed with ProbeDesign software (Roche). We evaluated the association of rs8179090 in TIMP-2 with curve initiation and progression by comparing risk allele frequencies between both cases and controls and between 2 groups according to progression status.

**Results and discussion**

We evaluated both cases (*N* = 100) and controls (*N* = 100) and the progression (*N* = 53) and nonprogression (*N* = 47) subjects. Mean final Cobb angle (SD) for cases group was 41 (19,98) degrees, and 29,5 (8,3) degrees and 55,5 (20,6) degrees for nonprogression and progression groups respectively. Mean Peak APR for the progression group was 14,7 deg/year and 2,3 deg/year. Allele frequencies found in control group were GG = 17%; GC = 30%, CC = 53% and for the cases group were GG = 21%;GC = 32%, CC = 45%. Those frequencies were not significantly different both in cases/control comparison (*p* = 0.59 OR =0,7) and progression/nonprogression analysis (*p* = 0.37 OR = 1,96). We found no replication of the association on IS curve progression nor initiation with rs8179090.

**Conclusion and significance**

The associations of the rs8179090 and TIMP-2 gene with IS occurrence and progression are not definite. Further association studies based on suggested clinical criteria for progression would be necessary to identify SNPs associated with the curve progression.

## O20 Interleukin 6 gene polymorphism in patients with degenerative lumbar scoliosis

### Jung Sub Lee, Jong Ki Shin, Tae Sik Goh, Seung Min Son

#### Pusan National University School of Medicine, Busan, South Korea

##### **Correspondence:** Jung Sub Lee

**Introduction**

Low bone mass and a female gender increase susceptibility to the development of degenerative lumbar scoliosis (DLS), which suggests the potential involvement of an osteoporosis-related gene in the pathogenesis of DLS.

Interleukin6 (IL6) is a multifunctional cytokine required for osteoclast differentiation and function, and IL6 and its receptor have been suggested to be associated with bone loss. However, no previous report has indicated an association between bone mass in DLS patients and IL6 gene polymorphisms.

**Objectives**

In the present study, the authors studied the relations between polymorphisms of IL6 gene and DLS in a patient cohort.

**Methods**

One hundred and eighty-four patients with a diagnosis of DLS and 220 normal healthy control subjects were prospectively enrolled. The inclusion criteria of this study were as follows: aged older than 40 years, no history of scoliosis or spinal trauma, and no prior spinal surgery. In addition, all patients fulfilled radiographic criteria (Cobb’s angle of > 10°). The authors determined the presence of the -597 G/A, -572 G/C and -174 G/C polymorphisms, measured bone mineral densities at the lumbar spine (LSBMD) and femoral neck (FNBMD), assessed radiological findings including lumbar scoliosis and lateral listhesis, investigated biochemical markers of bone turnover, and compared these results obtained with those of 220 healthy normal controls.

**Results and discussion**

The genotype frequencies of all studied SNPs were determined by screening DNA samples from the 404 study subjects. Genotype frequency distributions of the three polymorphic SNPs were in Hardy-Weinberg equilibrium. A comparison of the genotype frequencies of the 184 DLS patients and 220 controls revealed a significant difference for the IL6-572 G/C polymorphism (*P* = 0.0168). IL6-597 G/A and IL6-174 G/C were completely linked and showed very low rare allele frequencies in both groups. The two study groups were compared with respect to genotype, age, BMI, and biochemical markers for each DLS genotype subgroups. No significant differences were identified. Mean LSBMD was lower in DLS patients than in controls (*P* = 0.0388). The IL6-572 G/C polymorphism was significantly associated with LSBMD but not FNBMD, lumbar scoliosis, or lateral listhesis. The LSBMD in DLS patients with the CC genotype was significantly higher than in DLS patients with the GC (*P* < 0.05) or GG (*P* < 0.05) genotypes. Allele frequencies of IL6-597 G/A and IL6-174 G/A in the DLS group were too low for statistical analysis.

**Conclusion and significance**

Summary, we examined the association between BMD and the IL6-572 G/C gene polymorphisms in Korean DLS patients. The IL6-572 G/C gene polymorphism was found to influence LSBMD but no definitive mechanism for low bone mass in DLS was elucidated.

## O21 Ultrastructure change of lacuna-canalicular network - new insight into abnormal bone mineralization in AIS

### Huanxiong Chen^1,2,3^, Wayne Yuk Wai Lee^1,2,3^, Jiajun Zhang^1,2,3^, Elisa Man Shan Tam^1,2,3^, Gene Chi Wai Man^1,2,3^, Tsz Ping Lam^1,2,3^, Bobby Kin Wah Ng^1,2,3^, Yong Qiu^2,3,4^, Jack Chun Yiu Cheng^1,2,3^

#### ^1^Department of Orthopaedics and Traumatology, The Chinese University of Hong Kong, Shatin, N.T., Hong Kong; ^2^Joint Scoliosis Research Center of the Chinese University of Hong Kong and Nanjing University, Nanjing and Hong Kong, People’s Republic of China; ^3^SH Ho Scoliosis Research Laboratory, The Chinese University of Hong Kong, Shatin, NT, Hong Kong; ^4^Spine Surgery, The Affiliated Drum Tower Hospital of Nanjing University Medical School, Nanjing, China

##### **Correspondence:** Jack Chun Yiu Cheng

**Introduction**

The etiopathogenesis of adolescent idiopathic scoliosis (AIS) is largely unknown and is believed to be multifactorial. Amongst these, we have identified systemic low bone mineral density and bone microstructure as important prognostic factors for curve progression. Osteocytes is now regarded as a key player in regulating bone metabolism, and change in the connectivity of their lacuno-canalicular network (LCN) has been reported to be associated with diverse skeletal disorders. We hypothesized that abnormalities in the osteocytes' activities and ultrastructure of LCN might manifest phenotypically as abnormal bone quality, thus affecting bone mineralization and bone strength, and contributing to the initiation/progression of AIS.

**Objectives**

To study the ultrastructure of LCN and the association with bone mineralization and mechanical property between AIS and control subjects.

**Methods**

This was a case-control study on trabecular iliac crest bone biopsies taken intra-operatively from 5 severe AIS patients and 5 age-matched controls with strict IRB approval. The biopsies were divided into several pieces for (i) histomorphometry; (ii) acid-etched scanning electron microscopy (SEM), (iii) confocal microscopy with quantitative fluorescein isothiocyanate (FITC)-Imaris technique; (iv) energy-dispersive X-ray spectrometry (EDX) and (v) microindentation. All results were presented in mean ± standard deviation. Statistical analyses were performed with Mann-Whitney test.

**Results and discussion**

The mean age of AIS and control subjects were 16.00 ± 2.24 and 17.60 ± 4.88 years respectively (*p* = 0.671). Mean Cobb angle of the AIS subjects was 51.00 ± 8.31 degrees. Compared with the aged-matched controls, bone histomorphometry showed significantly lower osteocyte density and higher osteoid volume in AIS. SEM and FITC-Imaris technique showed abnormal clusters of irregular roundish shape osteocytes with short and disorganized canaliculae in AIS in contrast to the well-organized LCN and osteocytes with clearly spotted spindle cell body and longer canaliculi protruded perpendicularly from the cell bodies in the control. Comparing to the control, EDX showed significantly lower calcium to phosphorous ratio in the peri-osteocytic matrix of AIS (*p* = 0.047), which was associated with lower bone mechanical properties of AIS were profound (33% reduction in elastic modulus and 6% reduction in micro-hardness with *p* = 0.017 and *p* = 0.215, respectively).

**Conclusion and significance**

To the best of our knowledge, this is the first ex vivo study reporting the association of abnormal ultrastructure of LCN to lower bone mineralization and mechanical property in AIS.

Significant ultrastructural change in LCN and abnormal matrix mineral content and mechanical properties suggested the pathogenic role of bone metabolism in AIS. Further study is warranted to investigate the association of osteocytes activities with curve severity.

**Acknowledgements**

This project is supported by HK Research Grants Council (463113 and 14116415), and CUHK Direct grant (4054190).

## O22 Observed length increases of magnetically controlled growing rods are lower than programmed

### Mark Schwartz, Sarah Gilday, Donita I. Bylski-Austrow, David L. Glos, Lindsay Schultz, Sara O'Hara, Viral V. Jain, Peter F. Sturm

#### Cincinnati Children's Hospital Medical Center, Cincinnati, OH, USA

##### **Correspondence:** Peter F. Sturm

**Introduction**

Magnetically controlled growing rod constructs are used in the treatment of early onset scoliosis (EOS) as an alternative to traditional growing rods to reduce the need for multiple surgeries. The primary purpose of this study was to determine whether the actual rod displacements were different than the length increases programmed into the external remote controller. Secondary goals were to determine whether previous placement of spinal instrumentation was a factor in the amount of lengthening achieved, and if tissue depth between the skin surface and the rod correlated with the length increases.

**Methods**

A retrospective study (IRB approved) was performed of consecutive patients after implantation of a magnetically activated growing rod construct. Inclusion criteria included placement of the magnetically controlled growing rod as either an index procedure or replacement of traditional growing rod construct. Rod displacements were measured using ultrasound. Differences between programmed and actual displacements were determined by paired two-tailed t-test (α = 0.05). Summary statistics were calculated for two groups of patients based upon prior instrumentation history. Regression and correlation were used to determine the relationship between tissue depth and length increases.

**Results and discussion**

Thirty-one patents were included, 18 males, 13 females, age 8.1 years (±2.5), main curvature 60° (±14.6) at time of magnetic rod insertion. Twelve patients had prior instrumentation. Tissue depth was measured in 20 of the 31 patients. The total length increase achieved relative to the programmed distraction was 86.1% (±21) (*p* < 0.001). Increases in rod length for patients with and without prior surgery were 86.6% (±23) and 85.8% (±19), respectively. Total lengthening was inversely proportional to tissue depth (*r* ^2^ = 0.38, *p* < 0.005); the relative decrease in lengthening achieved was 2.1%/mm.

**Conclusions and significance**

In this clinical study of magnetically controlled growing rods, actual increases in rod length were within 14% of, and significantly lower than, the programmed displacement. Prior instrumentation did not decrease lengthening effectiveness. The distance between the rod and skin surface did affect the magnitude of distraction. Long-term studies of magnetically controlled growing rods are necessary to better determine what factors potentially affect the effectiveness of lengthening.

## O23 Biomechanical effect of pedicle screw distribution in AIS instrumentation using segmental translation technique: Preliminary results

### Xiaoyu Wang^1,2^, Dennis G. Crandall^3^, Stefan Parent^2^, Noelle Larson^4^, Hubert Labelle^2^, Carl-Eric Aubin^1,2^

#### ^1^Polytechnique Montréal, Montréal, Canada; ^2^Sainte-Justine University Hospital Center, Montréal, Canada; ^3^Sonoran Spine Center and Research Foundation, Phoenix, AZ, USA; ^4^Mayo Clinic, Rochester, MN, USA

##### **Correspondence:** Xiaoyu Wang

**Introduction**

Wide variation persists regarding the number and distribution of screws in AIS instrumentation. Instrumentation with fewer screws may have benefits. Clinical efforts to reduce the number of implants are hampered by a lack of biomechanical studies; there is a significant need for knowledge on the effect of screw distribution.

**Objectives**

The objectives were to evaluate different screw patterns with screw dropouts on the convex vs. concave side at alternate vs. periapical levels and determine whether equivalent deformity correction can be achieved with similar bone-screw force levels.

**Methods**

The study was performed through spinal instrumentation simulations on 3 AIS cases using computerized patient-specific biomechanical models. The 3 cases were two Lenke 1A and one Lenke 3B. The preoperative main thoracic (MT) Cobb angles were 55° with thoracic kyphosis (TK) of 7°, 23° and 37°, and apical vertebral rotations (AVR) of 18°, 17°, and 6° respectively. We simulated simultaneous segmental translation with one reference (bilateral screws at every level fused) and four alternative screw patterns that used 5 fewer screws. Screw dropouts were respectively on the convex and concave sides at alternate levels or periapical levels. Deformity corrections and bone-screw forces were compared.

**Results and discussion**

MT Cobb angles with alternative screw patterns were 3° to 5° to the reference screw pattern. The simulated TK with alternative screw patterns were within 2° to the reference screw pattern. Differences were less than 2° in the simulated AVR. Bone-screw forces were 65 ± 35 N (21 - 136 N), 47 ± 26 N (17 - 122 N), and 133 ± 64 N (14 - 21 N) with the reference screw pattern. Alternative screw patterns had 2 to 34% higher bone-screw forces in 10 of the 12 simulations while they were 22% and 25% lower in 2 simulations. The highest bone-screw forces with the alternative screw patterns were less than 47% higher in 10 simulations, but they were 61% and 114% higher in 2 simulations. The highest bone-screw force was 468 N observed in case #3 with convex alternate screw dropouts, which was lower than the average reported bone-screw connection strength. Highest forces in concave side periapical screw dropout patterns as compared to the convex side dropout patterns were 17% to 37% higher. Concave screw dropouts vs. convex screw dropouts and alternate screw dropouts as opposed to periapical screw dropouts resulted in higher maximum bone-screw forces in more simulations.

**Conclusion and significance**

According to biomechanical results, deformity correction can be achieved with alternative screw patterns using 23% fewer screws, but with about 25% higher bone-screw forces. Convex side screw dropouts as opposed to concave side and periapical level screw dropouts vs. alternate levels seem more likely to result in lower bone-screw forces. This study provided preliminary biomechanical evidence on deformity correction using alternative screw densities and some insight into the effects of screw dropout locations.

## O24 Patient cushion interface pressure and LFCN injury during scoliosis surgery

### Negar Behzadi Fard^1^, Sarah Southon^2^, Marc Moreau^3^, Douglas Hedden^3^, Kajsa Duke^1,3^

#### ^1^Department of Mechanical Engineering, University of Alberta, Edmonton, Canada; ^2^Alberta Health Services, University of Alberta Hospital, Edmonton, Canada; ^3^Division of Orthopaedic Surgery, University of Alberta, Edmonton, Canada

##### **Correspondence:** Kajsa Duke

**Introduction**

During scoliosis surgery damage to the lateral femoral cutaneous nerve (LFCN) has been reported in 18%-24% of patients. It is hypothesised that high intraoperative cushion interface pressure can cause LFCN injury. In order to measure the pressure a Force Sensing Array (FSA) pressure mapping system, similar to those used on wheelchairs, were used in this study. Other factors, like BMI and the duration of surgery, have been shown to be important in LFCN injury.

**Objectives**

The objective was to characterize intraoperative pressures at the patient cushion interface and examine the effect of BMI and duration of the surgery on the incidence of LFCN injury.

**Methods**

Twenty-three patients with adolescent idiopathic scoliosis, two male and twenty-one females, age range 9-18 years, were recruited from the University of Alberta Stollery Children's Hospital. A set of four FSA pressure mats (Vista Medical, Winnipeg), are used to measure intraoperative pressure. Data collection began after patient positioning on the surgery table and continued during the screw replacement, rod insertion and until closure. At the end of operation, pictures were taken of the redness on both hips. A post-operation follow-up form including: age, gender, BMI, weight, height as well as light touch sensation exams around the iliac area to detect numbness or tingling. The average pressure on the mats was plotted over time and the peek pressures were also recorded. Results were compared between patients with and without redness and LFCN problem after the surgery.

**Results and discussion**

Overall eleven patients (48%) had problems such as redness on their hip after the surgery. Of those, four (17%) had numbness and tingling in the LFCN region post-op. The average overall pressure (mmHg) was 29.4 and 26.7 for the redness and normal groups respectively. Higher average peek pressure of 264.4 was seen in the redness group compared to the normal group 236.5. Comparing the group that had numbness and LFCN injury the overall pressure was 30.6 compared to 27.3 for the normal. Similarly, their peek pressures were also higher at 254 versus 246. Expected trends were seen in all groups however none of the differences were statically significant. All four patients with LFCN numbness were of normal BMI 21.7-23. There were no significant differences found related to BMI and redness. As the scoliosis surgery is a lengthy surgery, it is important to investigate the effect of surgery duration. The average time for patients with LFCN injury was 350 (±54) minutes, significantly greater (*p* = 0.006), compared to 264 (±79) minutes for the normal group.

**Conclusion and significance**

Higher cushion interface pressures showed a trend for LFCN injury and redness but this was not statistically significant. Intra-operative time was the only factor that had a significant effect on LFCN injury. Due to the limitations with this study, further investigations is needed to determine if there is a pressure time threshold that should be avoided in order to reduce LFCN injury during AIS surgery.

## O25 Low dose postoperative ketamine as an adjuvant analgesic: A review of 50 pediatric patients with adolescent idiopathic scoliosis (AIS)

### Sarah Southon^1^, Leeann Lukenchuk^1^, Matthew Kerslake^1^, Geraldine Huynh^1^, Jill Chorney^2^, Ban Tsui^1^

#### ^1^Stollery Children's Hospital, Edmonton, Alberta, Canada; ^2^IWK Health Centre, Halifax, Nova Scotia, Canada

##### **Correspondence:** Ban Tsui

**Introduction**

Achieving optimal pain control in children after scoliosis surgery remains a complex problem. Opioids are the gold standard, but they may not control pain adequately and are associated with side unpleasant effects. Ketamine, an NMDA receptor antagonist, can attenuate development of acute tolerance to opioid analgesia and suppress rebound analgesia in animals, and its use in children as an anesthetic and analgesic is well-documented. Nonetheless, several meta-analyses provide conflicting evidence regarding the efficacy of ketamine for peri-operative analgesia in adults. To date, there is limited information on the use of ketamine for pain control in pediatric patients.

**Objectives**

To assess the effects of low-dose ketamine infusion as an adjuvant analgesic for children undergoing scoliosis surgery.

**Methods**

With the permission from the lead investigator of the larger Post-Operative Recovery following Spinal Correction: Home Experience (PORSCHE) study, and with local institutional ethics approval, we analyzed data from study patients recruited locally. Data were collected prospectively from 51 pediatric patients with AIS undergoing posterior instrumentation and fusion. Included were patients who received or not receive a postoperative ketamine infusion as part of their pain control regimen. All patients received patient-controlled analgesia (PCA) morphine/hydromorphone as part of their analgesic plan. Data, including method of analgesia, side effects, and patient satisfaction with pain control, were recorded.

**Results and discussion**

Data from 47 patients were analyzed. Four patients were excluded: one did not receive surgery, and three withdrew. Thirty patients received ketamine postoperatively, while 17 received PCA only. Mean patient age was 14.7 ± 1.6 years old, and mean patient weight was 53.8 ± 11.0 kg. On average, ketamine infusions were run for 2.1 ± 2.0 days. Comparing ketamine and non-ketamine groups, outcomes were not significantly different with respect to total days on bowel medications (*p* = 0.05), PCA use (*p* = 0.20), total days on anti-emetics (*p* = 0.69), or total days in hospital (*p* = 0.68). The non-ketamine group reported significantly higher satisfaction with pain control on post-operative day one (*p* = 0.03), but patient satisfaction was similar between groups on post-operative days 2-6. Together, these results suggest that ketamine has little effect as an adjunct to PCA opioid for pain control following scoliosis surgery in children.

**Conclusion and significance**

In contrast to early anecdotal evidence, this prospective study found no statistically or clinically significant benefits of ketamine as a post-operative adjunct analgesic for surgery in AIS. Since this local analysis is part of a larger multi-centre study, our study is not powered adequately for the small sample size. Overall, we did not observe any trends toward a positive outcome when ketamine was included in the post-operative analgesia regimen.

## O26 Avoiding spinal fusion: Is it time to revisit conservative therapy?

### Daniel Tobert^2^, Prachi Bakarania^1^, Hagit Berdishevsky^1^, Kelly Grimes^1^, Hiroko Matsumoto^1^, Joshua Hyman^1^, Benjamin Roye^1^, David Roye^1^, Michael Vitale^1^

#### ^1^Columbia University Medical Center/Morgan Stanley Children's Hospital, New York, USA; ^2^Harvard Medical School, Boston, USA

##### **Correspondence:** Prachi Bakarania

**Introduction**

The role of physiotherapeutic scoliosis specific exercises (PSSEs) remains controversial in the conservative management of adolescent idiopathic scoliosis (AIS). In the last 3 years there have been several RCTs that have shown the benefits of PSSEs compared to monitoring alone. To date, there are no multicenter long term studies that have looked at the efficacy in the US. For this reason, physical therapists have been finding it difficult to convince third party payers to reimburse for physical therapy visits.

**Objectives**

The purpose of this study is to examine patient and parent preferences to enroll in a RCT that compares PSSEs to current standards of care.

**Methods**

Patients with AIS and are skeletally immature (Risser <4 or <1 year postmenarachal) were enrolled. Exclusion criteria were previous spine surgery and non-English speakers. Hypothetical Trial: A 20 item (patient) and 25-item (caregiver) questionnaire assessed basic demographic information and patient willingness to participate. All participants were informed of the two treatment arms of the study: either continuing with monitoring or enrollment in learning PSSEs with a certified scoliosis physical therapist (PT). Intervention would include four, five-hour sessions over two weeks to learn basic PSSEs, 30 minutes daily exercise, and postural correction throughout the day. The PTs would follow up at 3-6 month intervals and measure Cobb angles, angle of trunk rotation with a scoliometer. Outcome measure used are SRS-22, Spinal appearance questionnaire and the VAS scale.

**Results and discussion**

77% (63% of patients, 93% of caregivers) agreed to participate in the RCT. Agreement of both the patient and caregiver to participate occurred 64% of the time. Of those willing to participate, the top reasons cited by patients included 'Chance to try new treatment' (50%), 'Doctor encouraged participation' (30%), and 'Interest in research' (10%). The top reasons cited by caregivers for participation included 'Chance to try new treatment' (57%), 'Doctor encouraged participation' (29%), and 'Interest in research' (10%). Forty percent of patients and 14% of caregivers reported they would drop out of the trial if the time commitment was too great. Of the patients unwilling to participate, the reasons include 'Potential time commitment' (86%) and 'Desire for doctor to decide treatment' (14%). One caregiver did not want to participate due to a 'Desire for the doctor to decide treatment'. This study was performed to understand what factors would hinder a proposed trial. Time commitment was a major reason not to participate.

**Conclusion and significance**

Conservative management of AIS has long been debated and definitive evidence on the subject is needed to better inform providers, patients and their caregivers. This study provides evidence that a large-scale multicenter trial comparing PSSEs to current standards of care is feasible and provides important information for its implementation.

## O27 Current knowledge of scoliosis in physiotherapy students trained in the UK

### Jason Black^1^, Michael Bradley^1^, Shawn Drake^2^, David Glynn^3^, Erika Maude^1^

#### ^1^Scoliosis SOS, London, United Kingdom; ^2^Arkansas State University, Jonesboro, AR, United States; ^3^Independant, York, United Kingdom

##### **Correspondence:** Jason Black

**Introduction**

It has previously been highlighted in both Poland and the United States that knowledge of Idiopathic Scoliosis (IS) among physiotherapy students is limited with respect to the SOSORT Guidelines. Early detection of scoliosis and correct initial management is essential in effective scoliosis care, and thus physiotherapists should be aware of the basic criteria for diagnosis and indications for treatment.

**Objectives**

The aim of this study was to evaluate the basic knowledge of idiopathic scoliosis in physiotherapy students trained in the UK.

**Methods**

A previously designed and tested 10-question survey, including knowledge of SOSORT Guidelines, 2011 was transcribed onto an online-survey platform. Questions were designed to analyse knowledge of: definition, cause, development, prevalence, diagnosis, treatment and bracing of scoliosis.

All physiotherapy-based UK universities were invited to participate, with the programme lead of each institution asked to distribute the questionnaire among the target population of final and penultimate year physiotherapy students (Master's and Bachelor's Degrees). The final number of students who received the study invitation is unknown. The survey link closed after 8 weeks of data collection.

**Results and discussion**

A total of 206 students, split over 12 institutions successfully completed the questionnaire.

Analysis showed that 79% of students recognised when Idiopathic Scoliosis (IS) is likely to develop, yet only 52% recognised that Idiopathic Scoliosis’ aetiology is unknown. 88% of students incorrectly defined IS as a 2-dimensional deformity, with only 24% of students successful in recognising the prevalence of IS within the scoliosis population. 12% of students could recognise the criteria for diagnosis and 93% were unable to recognise the appropriate treatment approach through therapeutic exercise. Finally 54% students managed to correctly identify when bracing is recommended for IS.

In comparison to previous studies within the US, UK students performed worse in relation to all questions except treatment (7% answered correctly vs 3% in the US)

**Conclusion and significance**

With only 7% of students able to answer >50% of the survey questions correctly, there is a clear lack of knowledge of appropriate Idiopathic Scoliosis diagnosis and care which could directly impact the information these patients are given within first contact primary care in the UK.

## O28 Teen compliance with Schroth therapy: is it possible?

### Hagit Berdishevsky, Amelia Lindgren, Prachi Bakarania, Kelly Grimes, Hiroko Matsumoto, Nicholas Feinberg, Zachary Bloom, David Roye, Michael Vitale

#### Columbia University Medical Center/Morgan Stanley Children's Hospital, New York, NY, USA

##### **Correspondence:** Hagit Berdishevsky

**Introduction**

Schroth therapy is a unique avenue of conservative care for patients with adolescent idiopathic scoliosis (AIS). Thus far, there have been very few and small studies to determine the efficacy of the treatment and its role in scoliosis management.

**Objectives**

To conduct a retrospective review to determine how many patients who initiate training, follow through to completion (attending 10 sessions). And to determine the compliance of patients who have finished formal training and transition to a home exercise program (HEP), with compliance defined as exercising for at least 80 minutes per week. The goal is to better understand patient behavior to identify how to design a prospective study to investigate the efficacy of Schroth.

**Methods**

Patients with AIS, seen at our institution and received a referral for Schroth therapy in 2014-2015, were screened. 88 patients met our inclusion criteria: a diagnosis of AIS, attended at least one Schroth session and therapy was completed prior to PSIF. Patient records were reviewed for the number of Schroth visits attended. A retrospective compliance survey was conducted to determine adherence to HEP following the last formal session, 3 months later and at the time of survey. Level III study.

**Results and discussion**

Seventy one patients completed the survey, 13 could not be reached and 4 declined to participate, for a response rate of 80.7%. Average patient age was 12.9 years (8-18), 86.4% were female. 31 patients (35.2%) successfully completed Schroth training. Following formal SSE training, 36.6% were complaint with HEP, 38.0% completed some HEP, while 25.4% did not complete any HEP. 3 months following therapy, 25.5% were compliant with HEP, 25.5% did partial HEP, while those who were noncompliant increased to 49%. Of those who completed the long term follow up: 27.4% of patients remained compliant with the HEP, 16.2% performed some HEP, however 56.5% of patients did not practice HEP. When stratified by completion of Schroth training, completers were compliant with HEP: 48% at 1 week after training, 40% at 3 months, and 50% at time of survey. For those who did not complete Schroth training, 30.4% were compliant at 1 week, 19.4% at 3 months, which dropped to 15.9% at time of survey. There was no significant difference in training completion or HEP compliance based on age or gender.

**Conclusion and significance**

In our study, ~35% of patients completed the recommended 10 training sessions. It is interesting that both completers and noncompleters participated in a HEP and patients were compliant in both groups at all time points. A significantly higher proportion of those who completed Schroth training remained compliant with HEP at all time points. Overall, there was a general trend for decreased participation in HEP the farther out from formal training the patient was. Identifying a significantly large cohort of patients who will remain compliant with Schroth HEP will be a major hurdle to developing a study to determine the efficacy of Schroth therapy and its role in the treatment of AIS.

## O29 Biomechanical modeling of global postural re-education and curve reducibility in physiotherapy treatment of adolescent idiopathic scoliosis

### Sarah Dupuis^1,2^, Carole Fortin^2,3^, Christiane Caouette^1,2^, Carl-Éric Aubin^1,2,3^

#### ^1^Polytechnique Montréal, Montréal, Canada; ^2^CHU Sainte-Justine, Montréal, Canada; ^3^Université de Montréal, Montréal, Canada

##### **Correspondence:** Carole Fortin

**Introduction**

Global Postural Re-education (GPR) is an approach used as a physiotherapy treatment for adolescent idiopathic scoliosis (AIS). In GPR, the physiotherapist evaluates trunk reducibility potential through manual correction and patient's self-correction postures. The correction of the postures relies on the experience of the physiotherapist and there is no quantitative tool to assess curvature reduction as well as treatment planning and follow-up.

**Objectives**

To evaluate the biomechanics of two GPR treatment postures (physiotherapist's manual correction and patient's self-correction capacity) using a computer modeling approach.

**Methods**

Study Design: biomechanical analysis. Level of evidence: N/A. The finite element model of 3 AIS cases (thoracic/lumbar Cobb, kyphosis, lordosis: P1) 32°, 27°, 21°, 54°; P2) 43°, 43°, 15°, 56°; P3) 28°, 30°, 21°, 50°), who undergo a physiotherapy treatment, was built from a surface scan and a 3D radiographic reconstruction. The physiotherapist hand pressures were numerically represented by applying a pressure of 50kPa on the right side of the trunk under the apex and of 30kPa on the left side of the trunk at pelvic level. A reducibility index was defined as the ratio of the pressure applied by the simulated hand at thoracic apex over the scoliosis Cobb angle reduction (kPa/degree). Patient's self-correction was numerically represented by transforming the skin of the model from a deformed position to an undeformed position. The curvature reduction obtained from the simulations for the two postures was compared to the initial deformation and biomechanically assessed.

**Results and discussion**

The simulated AIS cases allowed to numerically reproduce the two GPR postures studied. The simulated physiotherapist's manual correction (50kPa at thoracic level) induced reducibility indices of 8, 18 and 26 kPa/° respectively for the 3 cases (a small index indicates a more malleable spine). Patient's self‐correction posture showed an average 25% and 10% reduction respectively for the lumbar and thoracic curves. Thoracic (T) and lumbar (L) Cobb angles before/during manual correction/during self‐correction were: P1) T: 32°/26°/30°, L: 27°/26°/22°; P2) T: 43°/40°/41°, L: 43°/39°/34°; P3) T: 28°/26°/23°, L: 30°/25°/20°. Kyphosis (K) and lordosis (L) before/during manual correction/during self‐ correction were: P1) K: 21°/21°/21°, L: 54°/58°/59°; P2) K: 15°/16°/17°, L: 56°/55°/56°; P3) K: 21°/20°/22°, L: 50°/51°/52°.

**Conclusion and significance**

The feasibility of the computer modeling approach was shown to biomechanically represent and evaluate two GPR postures. The reducibility index calculated from the manual correction posture contributes to quantify the trunk and muscular stiffness. The ongoing clinical study intends now to integrate the experimental recordings to refine the reducibility index, to improve the self-correction and to assess the biomechanics of correction integration.

## O30 Does exercise effect subjective visuel, postural and haptic perception in adolescent idiopathic scoliosis?

### Gozde Gur, Yavuz Yakut

#### Hacettepe University, Ankara, Turkey

##### **Correspondence:** Gozde Gur

**Introduction**

The right perception of subjective vertical has been reported to be important to provide and maintain upright posture and gait. It has been theorized that abnormal perception of vertical may cause defect on perception of body orientation on earth in idiopathic scoliosis. Core Stabilization is one of the most recent exercise approach aims to improve postural balance and spinal stabilization through controlling position of the trunk in static postures and functional activities. We hypothesized that core stabilization exercises (CSEs) may effect subjective visual, postural and/or haptic perception in adolescent idiopathic scoliosis (AIS).

**Objectives**

The aim of this study was to investigate the effects of CSEs on subjective visual, postural and haptic perception in patients with AIS.

**Methods**

Twelve patients with AIS (mean age 14.4 ± 1.9 years, Risser sign 2.1) were included in this study. Average Cobb angle of patients was 20.8 ± 8.2° for thoracic and 23.7 ± 6.3° for lumbar regions. Patients received 10-week CSEs program comprising two supervised sessions in the clinic and home-based exercises five times per week for approximately 60 min. Cobb's angle was measured on standard standing anterior-posterior spine radiograph at baseline. The following assessments were included: Subjective visual perception (SVP) test at the angles of 0° vertical, 90° horizontal, 30°, 45°, 60° left and 30°, 45°, 60° right, subjective postural perception (SPP) test at the angles of 0° vertical 30° left, 30° right using manually controlled laser liner device and subjective haptic perception (SHP) test at 0° vertical, 90° horizontal, 45° left and 45° right using wooden stick with angle gauge in a dark room. The deviations from true line for each angle were recorded. The measurements were conducted at baseline and at the end of the ten-week treatment period. Results were analyzed using the Wilcoxon Signed Rank Test to compare repeated measurements at two different time points.

**Results and discussion**

All SVP, SPP and SHP scores improved (*p* < 0.05) except for the angle of 45° right, 30°, 60° left for SVP test and 90° horizontal for SHV test (*p* > 0.05).

**Conclusion and significance**

In this study, CSEs provided improvement in visual, postural and haptic perception in patients with AIS. Further studies, which investigate the effects of different exercise therapies on subjective visual, postural and haptic perceptions and relation of perception with postural correction, are needed.

## O31 Intensive Schroth treatment for patients with scoliosis in Balkans

### Nikola Jevtić^1,2^, Sanja Schreiber^3^, Axel Hennes^4^, Milan Pantović^1^

#### ^1^University of Novi Sad, Faculty of Sport and Physical Education, Novi Sad, Serbia; ^2^Scolio Centar, Novi Sad, Serbia; ^3^University of Alberta, Department of Pediatrics, Edmonton, Canada; ^4^MVZ Bad Sobernheim GmbH, Bad Sobernheim, Germany

##### **Correspondence:** Nikola Jevtić

**Introduction**

Intensive Schroth scoliosis treatment (ISST) is an intensive 10-day rehabilitation camp for patients with scoliosis. To date, five ISST camps were held in Balkans since January 2014. The Schroth treatment consists of 3D neuromuscular exercises that aim to recalibrate the posture affected by scoliosis using the patient's ability to auto-correct. They are used in combination with activities of daily living to assure constant active correction throughout the day. Given that the standard of care for scoliosis in Balkans does not include physiotherapeutic scoliosis specific exercises, which have been shown to lead to improved scoliosis outcomes, including Cobb angles, back muscle endurance, and quality of life, there is a need for an alternative rehabilitation model.

**Objectives**

To present the protocol of the ISST camp and to test the change in back muscle endurance and leg flexibility following the five 10-day ISST camps in Balkans.

**Methods**

Upon arrival, the patients were presented with an overview of the Schroth method and the treatment protocol, followed by the clinical assessment. The baseline and final assessments included measurements of body weight, height, "sit&reach" test, and Biering-Sorensen back muscle endurance test (BS). A certified Schroth therapist classified patients into one of four Schroth curve types, which guides the exercise prescription. Following the assessment, each patient was prescribed a set of 8-12 exercises. The exercises were performed 1.5 hours three times a day (total of 4.5 h/day), with 1.5-hour break between the exercise sessions. On the fourth and eighth day, the third exercise session was substituted with a massage therapy or a visit to the swimming pool. The patients were recommended to continue with the home exercise program daily. The changes in BS and "sit&reach" tests were analyzed using dependent samples t-tests.

**Results and discussion**

The total of 42 patients participated in five ISST camps, of which nine participated in ≥2. Patients represented four Balkan countries: Serbia (*N* = 27), Bulgaria (*N* = 12), Macedonia (*N* = 2) and Slovenia (*N* = 1). The mean age was 15.60 (SD = 4.42) and the mean Cobb angle 35.5° (SD = 15.7°). Thirteen patients wore a brace. Thirty-three patients were diagnosed with adolescent idiopathic scoliosis (AIS), three with juvenile and six with adult scoliosis with onset in adolescence. There were three 3c, 11 3cp, 11 4c and 17 4cp Schroth curves. There was a statistically significant improvement in Biering-Sorensen back muscle endurance test hold time of 46.0 ± 41.0 sec, from 127.6 ± 66 sec to 173.6 ± 72 (*p* < 0.0001). Likewise, the flexibility was statistically significantly improved by 4.0 ± 4.8 cm (t(41) = -5.378, *p* < 0.0001), from 8.0 ± 13.0 to 12.0 ± 10.5.

**Conclusion and significance**

Schroth exercises are well accepted among patients with scoliosis and frequently requested by patients and their families in Balkans. Back muscle endurance and flexibility were improved following the 10-day ISST in patients with scoliosis.

## O32 Weaning results of a consecutive series of 73 adolescent idiopathic scoliosis treated by the new Lyon brace (ARTBRACE)

### Jean-Claude de Mauroy^1^, Frédéric Barral^2^, Sophie Pourret^2^

#### ^1^Clinique du parc, Lyon, France; ^2^Lecante Group, Lyon, France

##### **Correspondence:** Jean-Claude de Mauroy

**Introduction**

ART is the acronym for Asymmetry, Rigid, Torsion. The realization of this brace is based on new concepts (circled helicoid, global detorsion, soft contact, mayonnaise tube effect, axilla baby lift, wrench & bolt) The excellent in-brace correction has been proven on a consecutive series of 141 AIS selected according to the SRS criteria in a prospective database. Results at six months and a year were compared with those of the former Lyon brace and confirm that these good results remain constant.

**Objectives**

It is now possible to study the Cobb angle results of the first 73 AIS reaching the end of treatment. Are the first results at 1 year confirmed?

**Methods**

This is a prospective controlled cohort observational study based on ongoing database including 544 patients with AIS treated with ARTbrace from May 2013 to November 2015. Only primary curves were selected and lumbar scoliosis (Lenke 5) are excluded as treated with the short GTB brace. The weaning protocol is identical to that of the former Lyon brace and compliant with the SOSORT guidelines. Statistical analysis has been done with SPSS v20. For all variables, normality of data was ascertained by the Kolmogorov-Smirnov's test. All tests were two-sided, with significance set at p < 0.05. Results are presented as mean ± standard deviation (SD).

**Results and discussion**

The dropout rate calculated for the first 125 patients is 14%. The mean age is 14.44 years (SD = 1.32, range: 10‚Äì17). 62 patients are female (85%). The average Initial Cobb angle is 26.84° (SD = 7.15, range: 18-48°). The average In-brace Cobb angle is 6.19° (SD = 8.27, range: [-18]-34°). The average In-brace correction (Percent) is 79.4% (SD = 26.6, range: 28-150%). The average Weaning Cobb angle is 15.89° (SD = 9.40, range: [-4]-44°). The average Weaning correction (Percent) is 53.0% (SD = 25.6, range: 4-150%). Out of 100 curvatures (45 thoracic and 55 lumbar), no curvature is worsened by more than 5°, 17 are stable, and 83 have an improvement of more than 5°. Correlations: There is a negative correlation between initial angulation and end treatment percent correction, *r* = -0.414, *n* = 43, *p* = 0.001 for thoracic curves and less *r* = -0.292, *n* = 53, *p* = 0.034 for lumbar curves. There is a strong positive correlation between in-brace correction rate and end treatment correction rate, *r* = 0.684, *n* = 43, *p* = 0.000 for thoracic curves and *r* = 0.596, *n* = 53, *p* = 0.000 for lumbar curves. In our experience, the weaning results are very close to those 2 years after weaning and can be considered as significant. Patients are a little older (14.4 vs 13.4) and with lower initial angulation than in the general statistics (26.84 vs 29.61). The in-brace reduction is greater (79.4% vs 69.4%) which explains the outstanding weaning correction rate (50% vs 25% for the old Lyon brace).

**Conclusion and significance**

For the same indications and with the same management principles, we can confirm that: 1. The early treatment with low angle; 2. The quality of immediate in-brace correction are fundamental to successful outcome.

## O33 Immediate in-brace correction with the new Lyon brace (ARTbrace): results of 141 consecutive patients in accordance with SRS criteria for bracing studies

### Jean-Claude de Mauroy^1^, Frédéric Barral^2^, Sophie Pourret^2^

#### ^1^Clinique du Parc, Lyon, France; ^2^Lecante Group, Lyon, France

##### **Correspondence:** Jean-Claude de Mauroy

**Introduction**

All recent studies confirm that the outcome of a conservative orthopaedic treatment depends on the compliance and the effectiveness of bracing measured by immediate in-brace curve correction. There is a correlation between immediate in-brace correction and biomechanical effectiveness of brace treatment in adolescent idiopathic scoliosis. In a retrospective analysis of the immediate in-brace correction obtained from a consecutive series of patients treated by the major SOSORT teams, the conclusion was that an effective brace should be able to achieve 50% correction of the curve magnitude, immediately after application.

**Objectives**

The aim is to provide a point of comparison between different braces and study the factors determining the reduction.

**Methods**

This is a prospective controlled cohort observational study based on ongoing database including 544 patients with AIS treated with ARTbrace from May 2013 to November 2015. Only primary curves were selected, lumbar curves Lenke 5 are excluded as treated with the short GTB brace. The SRS criteria group consisted of 141 patients with 177 curves.

Brace checking is performed 3-4 days after brace delivery with EOS ultra-low dose.

Statistical analysis has been done with SPSS v20. For all variables, normality of data was ascertained by the Kolmogorov-Smirnov's test. All analyses were performed according to the intention-to-treat principle. All tests were two-sided, with significance set at *p* < 0.05. Results are presented as mean ± standard deviation (SD).

**Results and discussion**

All 141 patients were reviewed at the control: no drop out. The mean age was 12.92 years (SD = 1.39, range: 10-15). 125 patients are female (88.7%). The average initial Cobb was 29.62° (SD = 4.6, range: 25-40°). The average in-brace correction (Percent) was 72.5% (SD = 21.9, range: 29-140%). At the Thoracic level (*n* = 98). The average initial Cobb was 30.33° (SD = 4.6, range: 25-40°). The average in-brace Cobb was 10.04° (SD = 7.1, range: -12-29°). The average in-brace correction (Percent) was 67.6% (SD = 21.2, range: 29-140%), significant (*p* = 0.000). At the Thoraco-Lumbar and Lumbar for double major level (*n* = 75).

The average initial Cobb was 28.61° (SD = 4.1, range: 20-40°). The average in-brace Cobb was 6.36° (SD = 6.5, range: -9-25°). The average in-brace correction (Percent) was 78.8% (SD = 21.3, range: 40-136%), significant (*p* = 0.000). A Spearman's rank-order correlation was run to determine the relationship between initial thoracic angulation and in-brace correction (Percent). There was a strong, positive correlation between initial and in-brace correction, which was statistically significant (*r* = -.339, *p* = .001). There was also a strong, positive correlation between initial and in-brace correction for lumbar curves, which was statistically significant (*r* = -.296, *p* = .001). For the BrAIST study, average in-brace correction was 33% (*n* = 152, range: -48 to 100%)

**Conclusion and significance**

The in-brace correction obtained by the ARTbrace corrects at least two times more than conventional polyethylene braces.

## O34 Immediate in-brace correction in patients affected with idiopathic scoliosis: determining factors

### Angelo Gabriele Aulisa^1^, Vincenzo Guzzanti^1^, Marco Galli^2^, Francesco Falciglia^1^, Lorenzo Aulisa^2^

#### ^1^U.O.C. of Orthopedics and Traumatology, Children's Hospital Bambino Gesù, Institute of Scientific Research, P.zza S. Onofrio 4, Rome, 00165, Italy; ^2^Department of Orthopedics, University Hospital "Agostino Gemelli", Catholic University of the Sacred Heart School of Medicine, Rome, 00168, Italy

##### **Correspondence:** Angelo Gabriele Aulisa

**Introduction**

Clinical experience shows that it not always possible to achieve the same degree of scoliotic curve correction even if the brace is the same with similar modalities of applying the corrective actions.

**Objectives**

Aim of the study is to investigate other possible factors influencing the initial in-brace correction.

**Methods**

From our prospective database we retrieved 381 patients affected with idiopathic scoliosis below 40 Cobb degrees who underwent full time bracing till end of skeletal growth. Each patient underwent X-rays controls in teleradiography (2 meters, 36 X 91 films) in AP e LL view. For the present study, only the X-ray performed at two conventional times were considered: t0) beginning of treatment; t1) 3 months after treatment beginning. The following data were recorded: age at t0, age at menarche, Risser at t0, severity of the curve in Cobb degrees (at t0 and t1), severity of rotation in Pedriolle degrees (at t0 and t1). Patients were divided into two groups according to the response to initial in-brace correction. One group (Group A) included those patients who showed an "optimal" response to treatment defined as more than a 50% rate of curve correction in Cobb degree at t1. The other patients (those who got less than a 50% of curve correction at t1) were included in the group with a "sub-optimal" response to treatment (Group B). Logistic regression analysis was carried out assuming the "optimal" vs "sub-optimal" outcome as dependent variable.

**Results and discussion**

Group A included 146 patients with a mean curve severity of 26,5 (SD 4,6) Cobb degree at t0. Mean Cobb degree after initial in-brace correction (t1) was 9,1 (SD 3,8). Group B included 235 patients with a mean curve severity of 29,5 (SD 5,4) Cobb degree at t0. Mean Cobb degree after initial in-brace correction (t1) was 19,2 (SD 4,7). Logistic regression analysis gave the below equation L = 2,93 - 0,06 (Cobb degrees) - 0,148 * (Pedriolle degrees). The Cobb coefficient, though significant (*p* = 0,025), is far less significant than the Pedriolle's (*p* = 0,0004). By this equation the probability of the result can be estimated in any single patients and a probability >50% is assumed as indicative of the "optimal" result. The accuracy of this predictive test is about 70%.

**Conclusion and significance**

At the diagnosis the most determinant factor for the prediction of the initial response to treatment is the severity of vertebrae rotation in Pedriolle's degrees. Also the assessment in Cobb degrees is significant, though less important. The stepwise logistic model discarded other bio-physiological variables (age, sex, Risser and menarche).

## O35 Retrospective monocentric study on 64 teenagers treated by brace for Scheuermann's disease: evaluation of disability and quality of life once brace has been removed

### Jean-Claude Bernard^1^, Julie Deceuninck^2^, Eric Berthonnaud^1,2,3^, Adrien Rougelot^1^, Marie-Eva Pickering^1^, Emmanuelle Chaleat-Valayer^1^

#### ^1^Croix Rouge française-CMCR des Massues, Lyon, France; ^2^Laboratoire de physiologie de l'exercice, Université de Lyon, Saint Étienne, France; ^3^Hôpital Nord-Ouest, Villefranche sur Saône, France

##### **Correspondence:** Jean-Claude Bernard

“This abstract has not been included here as it has already been published.”

## O36 Orthotic treatment in patients with adult onset spinal deformity (AS): a systematic review

### Richard Webb^1^, Josette Bettany-Saltikov^2^, Barbara Neil^2^

#### ^1^Teesside University/Peacocks Medical Group, Newcastle, UK; ^2^Teesside University, Middlesbrough, UK

##### **Correspondence:** Richard Webb

**Introduction**

Studies from the USA have estimated the incidence of spinal deformity in the adult population (mean age 70.5 years) at up to 68% (Schwab et al., 2005). Patients with AS are increasing being referred to orthotists and other medical and allied health professionals for problems relating to increasing spinal deformity. Patients generally present with spinal pain, reduced mobility, decreased function and low self-esteem. Given the non-invasive nature and relatively low cost of orthotic treatment together with the increasing demographic of older people in the UK as well as worldwide, it is highly likely that orthotic referrals will increase significantly in the near future.

**Objectives**

To evaluate the efficacy of orthotic treatment in patients with adult onset spinal deformity (scoliosis or kyphosis).

**Methods**

Search Strategy: The following databases were searched with English Language limitations: NTIS, OpenGrey.org On-going research via online professional forums and other expert opinion, BAPO, ISPO AAOP - JPO AAOP CPO, CAPO, CENTRAL - Cochrane Library, EBSCO including, Cinahl -full text, MEDLINE AMED. Limiters to "all adult". English language only. Selection criteria: Randomised controlled trials, prospective cohort studies, quasi experimental designs, comparing orthotic treatments with no treatment, and other non-surgical interventions and co- interventions.

**Results and discussion**

Only four studies that matched the inclusion criteria were found. These included a randomised cross-over trial (Pfeifer, Begerow and Minne, 2004), a single blinded trial (Azadinia et al., 2013), Sinaki et al's study (2005) which had a quasi-experimental design and the final study which was a randomised, placebo control trial (Vogt et al., 2008). This systematic review looked for high quality evidence regarding the orthotic treatment of adult onset spinal deformities. The four papers that were found and reviewed, related to orthotic treatment of kyphosis in subjects of over 60 years of age all of which reported improved balance scores, reduction of deformity, muscle strength and pain after orthotic treatment. All four studies had a number of inherent weaknesses in study design, and to various extents internal and external validity. Given this and the small number of reports discovered the results should be viewed with caution.

**Conclusion and significance**

Given the predicted change in the age demographic worldwide, to a more elderly population, the authors recommend further research into the orthotic treatment of AS with particular emphasis placed on the full reporting (ie: comprehensive description of the orthotic design, material/construction) of the orthoses used, using valid and appropriate patient-oriented outcome measures. Finally, research is needed to establish the specific elements of orthotic design/materials that are important in producing positive outcomes. Establishing the precise relationship spinal orthosis design and construction and positive outcomes could lead to lighter and more effective devices with increased patient compliance in patients with AS.

## O37 Peak scoliosis brace can reduce pain in adults with painful scoliosis: results from a prospective cohort study

### Fabio Zaina^1^, Martina Poggio^1^, Sabrina Donzelli^1^, Monia Lusini^1^, Salvatore Minnella^1^, Stefano Negrini^2,3^

#### ^1^ISICO, Milan, Italy; ^2^Don Gnocchi Foundation, Brescia, Italy; ^3^Brescia University, Brescia, Italy

##### **Correspondence:** Fabio Zaina

**Introduction**

Adult scoliosis is sometime associated to back pain and severe curves can progress over time. The main approach for these patients is the surgical one, however surgery is not appropriate for all patients, and certain patients do not accept surgery. Despite scoliosis has been estimated to affect up to 68% of the population over 60, there is scant literature about conservative treatment for adult scoliosis. Custom fabricated rigid torso braces, similar to those commonly used for children are sometime used in adult patients, however, the goal of these braces is to correct and/or sustain the sagittal plane of patients, no data have been published on the efficacy of these braces in pain relief, and such braces are typically not well tolerated by adults. Recently a new brace has become available, the Peakª Scoliosis Brace (Aspen Medical Products) designed to alleviate pain for adult patients with chronic pain secondary to scoliosis.

**Objectives**

To test the efficacy of the Peakª Scoliosis Brace in reducing pain in adult scoliosis patients.

**Methods**

Design: prospective experimental cohort study. Population: 20 adults with back pain secondary to Idiopathic Scoliosis. The sample size calculation based on a pilot study. Inclusion criteria: Adults affected by Idiopathic scoliosis of 30¡ Cobb or more and chronic low back pain (cLBP). Exclusion criteria: secondary scoliosis. Outcome measures: NRS, Oswestry Disability Index (ODI), Roland Morris Questionnaire (RM), COMI. Statistical analysis: paired t-test. Protocol: patients were evaluated at baseline immediately before starting with the brace and after 1 month. The brace must be worn for at least 2 hours per day.

**Results and discussion**

Twenty out of 29 eligible female patients entered the study (age 67.8 ± 10.5, curve 61.9 ± 12.6¡ Cobb). We had no drop out. Worst pain (back or leg) and leg pain significantly improved from 7.15 to 5.85 and from 5.65 to 3.55 (*p* < 0.05), while back pain improvement didn't reach statistical significance. Six patients achieved the minimal clinically important difference of 2 points for worst pain, 12 for leg pain. RM improved (*p* < 0.05), no differences for ODI and COMI score.

**Conclusion and significance**

The Peak Scoliosis brace showed a significant improvement at 1 month of worst pain and leg pain in a group of adult women with scoliosis and cLBP. Back pain slightly improved, but the change was not statistically nor clinically significant. Also the quality of life didn't change in a significant way even if the patients reported satisfaction with the treatment. The follow up time was really short, it's possible that a longer treatment could be more effective.

## O38 Prospective study of 618 lumbar scoliosis treated with the short polyethylene detorsion brace GTB1

### Jean-Claude de Mauroy^1^, Frédéric Barral^2^, Alith Hoang^2^

#### ^1^Clinique du parc, Lyon, France; ^2^Lecante group, Lyon, France

##### **Correspondence:** Jean-Claude de Mauroy

**Introduction**

Fifty years ago the majority of North American braces were made in delordosis and extension. In Europe, the 3 points brace of Michel and Allegre was essentially a translation system in the frontal plane with extension by the iliolumbar thrust. The GTB1 of Jacques Griffet is based on lordosis and untwisting.

**Objectives**

The results on large homogenous prospective series have never been published to date.

**Methods**

This is a prospective controlled cohort observational study (Oxford Levels of Evidence 2) based on ongoing database including 618 consecutive patients with AIS treated with GTB1 from 1998 to 2013. The statistical treatment was performed using SPSS v20 pack. The overall untwisting of the lumbar spine requires two fixed points on the iliac crest and at the base of the rib cage. Geometric detorsion is obtained by a translation along the vertebral axis by increasing the distance between the iliac crest and the base of the thoracic cage. Mechanical detorsion is a rotation around the vertebral axis by translation of the soft tissues. The original curved anterior trim line provides stability and facilitates untwisting. The majority of patients in this series had an initial short plaster cast before the realization of the GTB1.

**Results and discussion**

One hundred fifty-six patients (25%) did not reach the end of treatment. The proportion of drop out is higher before 2013 when the plaster cast was preceding the GTB1 brace: (*n* = 428) = 26%. After 2013 with regional moulding the rate is: (*n* = 34) = 8%. There is no correlation between the angular in-brace reducibility and initial age at the beginning of treatment, *r* = 0.026, *n* = 456, *p* = 0.576. The angular in-brace reducibility does not depend on the age at the beginning of treatment. 240 scoliosis were controlled at least 2 years after brace weaning. The mean initial Cobb angle is 25.73¡ ± 5.71¡ at the mean age of 14.25 ± 1.76 years. The average treatment time is 15.84 ± 1.66 months. The in-brace Cobb angle is 6.02¡ ± 5.95¡, 77% of reducibility rate. The Cobb angle at the end of treatment is 15.33¡ ± 7.11¡ i.e. 40% of reduction rate. The Cobb angle 2 years after weaning is 16.32¡ ± 7.16¡ i.e. 6% of stability rate. 185 patients (77%) have an improvement of more than 5¡. 275 (59%) patients are night time wearing. The data can be compared to that of the Aulisa's PASB. PASB: *n* = 110 with initial Cobb = 29¡, In-brace = 14¡, Weaning = 12¡, After 2 years = 13¡. The mean initial angulation is higher for the PASB, which probably explains the lower in-brace reducibility. The fact of wearing the brace between 18 and 22 h/24, while more than half of our patients are night or 16 h/24 time wearing influences the final result with better results for the PASB.

**Conclusion and significance**

The drop-outs rate decreased significantly with the new correction protocol with regional moulding. The angular in-brace reducibility is high as well as the final result at the end of treatment and does not depend on the initial age. There is a good stability 2 years after brace weaning.

## O39 Outcome assessment of bracing in pubertal girls with idiopathic scoliosis: a longitudinal analysis until brace weaning or surgery

### Saihu Mao, Benlong Shi, Bangping Qian, Zezhang Zhu, Xu Sun, Yong Qiu

#### Spine Surgery, the Affiliated Drum Tower Hospital of Nanjing University Medical School, Nanjing, China

##### **Correspondence:** Yong Qiu

**Introduction**

Rapid longitudinal growth is widely accepted as a critical modifying factor for curve progression of idiopathic scoliosis (IS). The curve behavior by growth spurt, however, hasn't been analyzed critically.

**Objectives**

This study is to analyze the pubertal growth parameters, the influence of growth peak upon the angle velocity fluctuation and the bracing outcome in female patients with IS.

**Methods**

Seventy physically immature bracing female IS patients with open triradiate cartilage (averaged 10.5 yrs) were evaluated every three to six months through their growth spurt until Risser 4 and distal radius and ulna (DRU) classification reaching R10 or surgery. Serial measurements of Cobb angle and multi-dimensional maturity indicators involving chorological age, triradiate cartilage, Risser sign, height, simplified skeletal maturity scoring (SSMS) system and DRU classification were regularly assessed. The angle velocity (AV) fluctuation by onset of peak height growth velocity (PHV) was defined as alleviative if the AV was negative, otherwise it was progressive. Comparisons were made between the positive and negative AV groups using independent sample T test and crosstab analysis. Logistic regression analysis was used to identify the predictive factors of failed brace treatment.

**Results and discussion**

Brace treatment through the pubertal growth spurt demonstrated a failure rate of 57.1% and a surgical rate of 45.7%. Onset of PHV triggered the occurrence of alleviative AV in 54.3% of the recruited patients, which were associated with lower failure rate (36.8% vs. 81.2%, *p* = 0.016) and surgical rate (21.1% vs. 75.0%, *p* = 0.002). The timing and magnitude of PHV, as well as initial Cobb angle were comparable between these two groups (*p* > 0.05). Patients in the failure group showed greater ratio of thoracic curve (80.0% vs. 26.7% *p* = 0.002) and higher AV at growth peak (2.3 ± 9.1 vs. -6.5 ± 11.4¡/yrs *p* = 0.016). The logistic regression analysis revealed that negative AV by PHV (OR = 9.268, 95% CI = 1.279-67.137, *p* = 0.028) and thoracic curve type (OR = 13.391, 95% CI = 2.006-89.412, *p* = 0.007) were strong predictive factors of failed brace treatment.

**Conclusion and significance**

Bracing prescribed before pubertal spurt was associated with higher risk of failed brace treatment, especially for those with occurrence of alleviative AV by growth peak and curve pattern being major thoracic scoliosis.

## O40 Assessment of a brace design approach combining CAD/CAM and numerical modeling for the brace treatment of adolescent idiopathic scoliosis

### Nikita Cobetto^1,2^, Carl-Éric Aubin^1,2^, Stefan Parent^2,3^, Soraya Barch^2^, Isabelle Turgeon^2^, Hubert Labelle^2,3^

#### ^1^Polytechnique Montréal, Montréal, Canada; ^2^CHU Sainte-Justine, Montréal, Canada; ^3^University of Montréal, Montréal, Canada

##### **Correspondence:** Carl-Éric Aubin

**Introduction**

Bracing is the conservative treatment to prevent curve progression for adolescent idiopathic scoliosis (AIS) and correlation was found between immediate in-brace correction and brace treatment's long-term effectiveness. Recent studies also demonstrated that braces created with computer-aided design and manufacturing systems (CAD/CAM) are as effective as braces made with the traditional plaster-cast methods. Recent advances combining patient-specific finite element modeling (FEM) to a CAD/CAM system allow the iterative improvement of the brace design and the assessment of its biomechanical efficiency before its fabrication.

**Objectives**

To assess the immediate in-brace effectiveness of braces designed using CAD/CAM and FEM for the conservative treatment of AIS.

**Methods**

Sixty AIS patients were prospectively recruited and randomized into two groups. 31 patients were assigned to the control group for whom the brace was designed using a scan of patient's torso and a conventional CAD/CAM approach (CtrlBrace). The 33 other patients were assigned to the test group for whom the brace was designed using the scan of the torso, the CAD/CAM approach and a patient-specific FEM obtained from 3D reconstructions of the spine, rib cage and pelvis (FEMBrace). The FEMBrace design was then simulated and iteratively optimized to maximize the correction of the curve and to minimize the contact surface and material of the brace before its fabrication.

**Results and discussion**

Both groups had comparable age, sex, weight, height, curve type and severity. Scoliosis Research Society standardized criteria for bracing were followed. For the control group, average Cobb angle prior to bracing was 27° for thoracic (T) and lumbar (L) curves (SD 6°, min 15° and max 45°). For the test group, initial Cobb angles were 32° and 27° for thoracic (T) and lumbar (L) curves respectively (SD 7°, min 14° et max 43°). CtrlBraces reduced T and L curves by 8° (31%) and 11° (43%) respectively, compared to 14° (42%) (*p* < 0.04) and 14° (51%) for FEMBraces. FEMBraces Cobb angle correction was predicted with a difference inferior to 5°. On average, FEMBraces were 50% thinner and had 20% less covering surface than CtrlBraces.

**Conclusion and significance**

Braces designed using the CAD/CAM and 3D FEM simulation approach were more efficient and lighter than standard braces using CAD/CAM only at first immediate in-brace evaluation. This study is ongoing to recruit 100 patients and to analyze the long-term effect of bracing after 2 years. These results suggest that AIS brace effectiveness may be improved using this new design platform.

## O41 Mechanical and physiological effects of three dimensional scoliosis brace

### Hasan Md Arif Raihan^1^, Datta Tarit Kumar^1^, Chapal Khasnabis^2^, Ameed Equbal^1^, Ashis Kumar Chakraborty^3^, Abhishek Biswas^1^

#### ^1^The West Bengal University of Health Sciences & National Institute for the Orthopaedically Handicapped, Kolkata, India; ^2^World Health Organization, Geneva, Switzerland; ^3^Indian Statistical Institute, Kolkata, India

##### **Correspondence:** Datta Tarit Kumar

“This abstract has not been included here as it has already been published.”

## O42 Effect of a spinal brace on postural control in adolescent idiopathic scoliosis

### Gozde Gur^1^, Burcu Dilek^1^, Cigdem Ayhan^1^, Engin Simsek^2^, Ozgen Aras^3^, Songul Aksoy^4^, Yavuz Yakut^1^

#### ^1^Department of Physiotherapy and Rehabilitation, Faculty of Health Sciences, Hacettepe University, Ankara, Turkey; ^2^School of Physical Therapy and Rehabilitation, Dokuzeylul University, Izmir, Turkey; ^3^Department of Physiotherapy and Rehabilitation, School of Health Sciences, Dumlupinar University, Kutahya, Turkey; ^4^Department of Audiology, Voice and Speech Disorders, Faculty of Health Sciences, Hacettepe University, Ankara, Turkey

##### **Correspondence:** Gozde Gur

“This abstract has not been included here as it has already been published.”

## O43 Use of ultrasound imaging determines curve flexibility to predict in-brace correction

### Edmond Lou^1,2^, Doug Hill^2^, Rui Zheng^1^, Andreas Donauer^2^, Melissa Tilburn^2^, Jim Raso^2^, Marc Morau^1^, Douglas Hedden^1^

#### ^1^University of Alberta, Edmonton, Canada; ^2^Alberta Health Services, Edmonton, Canada

##### **Correspondence:** Edmond Lou

**Introduction**

For children with Adolescent Idiopathic Scoliosis (AIS), spinal flexibility affects correction attainable by bracing; more flexible spines should be more correctable in a brace. However, to date measurement of curve flexibility has relied on methods based on ionizing radiation. Our group developed an ultrasound spinal imaging method to measure quasi-Cobb angles and vertebral rotations reliably and repeatably. We extended our method to measure spinal flexibility during the brace design.

**Objectives**

To determine how to use curve flexibility information to predict in-brace correction.

**Methods**

Eleven AIS subjects (10 F, 1 M; Major Cobb: 38 ± 6o; 13.2 ± 1.6 years) prescribed full-time TLSO participated. During the casting clinic, subjects were scanned with ultrasound (US) in the prone position while performing maximum side bending, to both sides in turn. While scanning, all subjects kept their hips level and both shoulders in contact with the bed. Only the major treated curves were analyzed in this study. Spinal bending correction (flexibility) was calculated as [Pre-brace X-ray Cobb - US Bending Cobb] / Pre-brace X-ray Cobb x 100%. Final in-brace correction was calculated as [Pre-brace Cobb - In-brace Cobb] / Pre-brace Cobb x 100%. The association between the spinal flexibility and the in-brace correction was then determined.

**Results and discussion**

Curve flexibility and in-brace correction averaged 72 ± 14% (range: 51% - 89%) and 55 ± 21% (range: 32% - 90%), respectively. Thresholds of 60% for flexibility and 40% for in-brace correction were found to have association. Among 11 cases, 8 cases had both flexibility and in-brace correction above the thresholds and 2 cases had both below the thresholds. The only case not matching to this prediction in which the flexibility versus the in-brace correction was 53% vs 83%, respectively. Using the selected thresholds (60% flexibility, 40% in-brace correction), the positive predictive value (PPV) was 8/8 (100%), the negative predictive value (NPV) was 2/3 (67%), sensitivity of 8/9 (89%) and specificity of 2/2 (100%). All cases did not require adjustment. Knowing the flexibility assisted orthotists and surgeons to aim for optimum in-brace correction. Approximately 5 minutes were added to the clinic visit time: 1 minute to scan, 3 minutes to process and 1 minute to measure the parameters.

**Conclusion and significance**

Ultrasound imaging provides radiation-free real-time measures of spinal flexibility and it can be used to aim for optimum in-brace correction. More cases are required to validate the prediction model before it can be widely used.

## O44 Spinal flexibility assessment on the patients with adolescent idiopathic scoliosis (AIS)

### He Chen, Wong Man-Sang

#### Interdisciplinary Division of Biomedical Engineering, The Hong Kong Polytechnic University, Hong Kong, China

##### **Correspondence:** He Chen

**Introduction**

Spinal flexibility of the patients with adolescent idiopathic scoliosis (AIS) is one of the essential assessment parameters for clinical decision. Various spinal flexibility assessment methods have been proposed but there is a lack of consensus on their specific applications.

**Objectives**

To review contemporary spinal flexibility assessment methods on the patients with AIS.

**Methods**

The databases of AbleData, IBSS, Academic Search Premier, MEDLINE/PubMed, CINAHL, Native Health Databases, CIRRIE, RECAL Legacy, Compendex, REHABDATA, EMBASE, Global Health and Web of Science were searched. The study inclusive criteria are: 1) prospective study; 2) investigated spinal flexibility on patients with AIS; 3) published over the past two decades.

**Results and discussion**

Thirteen eligible articles were included in this review. Generally accepted assessment methods for spinal flexibility include lateral bending (standing/supine lateral-bending, fulcrum-bending, prone/supine with manual reduction) and traction (suspension, supine traction, supine traction under general anesthesia(UGA), supine traction with manual reduction and supine traction with manual reduction UGA). In general, tractions demonstrated more spinal flexibility/correction on severe curves and less on moderate curves versus side-bending tests. Among the side-bending methods, fulcrum-bending revealed more spinal flexibility/correction on main thoracic curves, while supine lateral-bending demonstrated more on proximal thoracic curves and lumbar curves. Prone with manual reduction was less accurate to predict the postoperative correction, but it offered better prediction not only in the last instrumented vertebra rotation and deviation from the midline but also in selective fusion of nonstructural curves and trunk balance. For the traction methods, the flexibility/correction was subjective to the traction or manual reduction force exerted to the spine. Traction UGA could avoid muscle spasm and reach to a higher correction rate than surgery. Radiography is a commonly used technique to assess spinal flexibility but it would expose patients to radiation risk. Some imaging techniques, such as MRI, Ultrasound, EOS and Photogrammetry, have a potential to evaluate spinal flexibility but they may not be readily available for widespread usage at present.

**Conclusion and significance**

The traction methods could be considered for the patients with severe curves, while the side-bending methods could be suggested for the moderate curves. Among various side-bending methods, the fulcrum bending is suggested to assess the flexibility of main thoracic curve whereas the supine side bending should be considered for the proximal thoracic, thoracolumbar or lumbar curves. A more comprehensive guideline for selection of appropriate method should be established via further studies. A non-invasive, user-friendly and reliable technique to assess spinal flexibility should be developed considering repetitive radiography to the patients with AIS in current practice.

## O45 Non radiographic methods of measuring global sagittal balance: a systematic review

### Larry Cohen, Sarah Kobayashi, Milena Simic, Sarah Dennis, Kathryn Refshauge, Evangelos Pappas

#### The University of Sydney, Sydney, Australia

##### **Correspondence:** Larry Cohen

**Introduction**

Progressive stooped posture, a common consequence of the ageing process, is associated with poor quality of life. This posture, which can be described according to the vertical alignment of the trunk over the pelvis, is defined as global sagittal balance and is termed anterior sagittal balance when exceeding, radiographically measured, predetermined threshold values. Due to the high cost and adverse radiation exposure, repeated radiographic measurement and monitoring of sagittal balance in the clinical setting is unsuitable. Non-radiographic methods of measuring global sagittal balance are available but their psychometric properties have not been evaluated systematically.

**Objectives**

The aim of this systematic review was to synthesise and evaluate the reliability and validity of the non-radiographic methods of assessing global sagittal balance.

**Methods**

Results from a systematic review of five electronic databases (MEDLINE, EMBASE, Web of Science, CINAHL and AMED) based around three main term groups (sagittal alignment, psychometric properties and physical tests) were screened by two independent reviewers. The reviewers extracted and evaluated, in consultation with a third adjudicating reviewer, the relevant data and article quality according to the 13 item Brink and Louw critical analysis tool (B&L CAT).

**Results and discussion**

The search strategy retrieved 3602 records. Following analysis, twelve articles describing six methodologies were identified. One article measured validity, one article validity and reliability and ten articles measured reliability. Average quality was 54% and only 3 articles rated as high quality scoring > 60% on the B&L CAT. Surface topography demonstrated moderate validity (*R* = 0.68 *p* < 0.001 & RMSD radiographic trunk inclination, of 1.07 cm for SVA) and high to very high reliability (ICC & Cronbach α range 0.84- 0.985).Spinal mouse determined sagittal balance has moderate to high reliability (ICC range 0.67-0.87) but its validity has not been investigated. Simple plumbline methods in two papers that did not score > 60% on the B&L CAT demonstrated high precision (0.9 mm-2.2 mm) and high reliability (ICC range 0.76- 0.86). There was little consistency among articles with respect to subject populations, reporting parameters and methods of evaluating agreement. This prevented a quantitative meta-analysis of pooled results.

**Conclusion and significance**

Our review established that non radiographic methods have demonstrated moderate to very high reliability however further work needs be undertaken with regard to their moderate validity, which is at the moment is limited, in poorer quality articles, to surface topography. The psychometric properties of lower cost methods such as the spinal mouse are moderately to highly reliable and the simple plumbline method which has also not been validated shows promising precision. The overall quality of studies was moderate to poor and there was a lack of homogeneity with regard to populations, parameters of measurement and statistical agreement methods.

## O46 A systematic review of torso motor control impairments in adolescents with idiopathic scoliosis (AIS) with implications for the planning of conservative interventions

### Fatemeh Aslanzadeh^1^, Eric C. Parent^1^, Brian MacIntosh^2^

#### ^1^University of Alberta, Edmonton, Canada; ^2^University of Calgary, Calgary, Canada

##### **Correspondence:** Eric C. Parent

**Introduction**

Fiber type abnormalities have been observed in AIS. However, because of the limited and conflicting knowledge on muscle involvement in the etiology and progression of AIS, a systematic review is needed. Scoliosis-specific exercise approaches have not yet stated their rationale based on motor control deficits of torso muscles. Such evidence could inform exercise prescription.

**Objectives**

The objectives were to systematically review the literature on differences in: 1) strength and electromyographic (EMG) activity of the erector spinae and abdominal muscles in AIS compared to healthy subjects. And 2) motor control deficits between the sides of spinal curvatures in patients with AIS compared to healthy controls.

**Methods**

A search was conducted in EMBASE, MEDLINE, CINAHL, Pedro, and Web of Science. Free text and indexed search terms described concepts such as: scoliosis, spinal deformity, strength, endurance, fatigability, muscle fatigue, latency, co-activation, EMG activity and timing. The references of included articles were reviewed. Two reviewers screened abstracts, then full-text articles using the inclusion criteria: in English or French; AIS; with torso motor control measurements. Exclusion criteria were: post-operative or post-exercise; or less than 10 subjects. Reviewers completed the Newcastle/Ottawa quality appraisal and the extraction form. This review followed the PRISMA guidelines.

**Results and discussion**

Our search yielded 10887 hits of which 6534 were unique records. After abstract screening 98 full-texts were reviewed and 27 were included. Inter-reviewer agreement for abstracts was Kappa = 0.79. No study met >50% of the quality criteria (no blind examiners; only 13% with details on missing data). Many studies had small sample size, unclear methodology, heterogeneity in curve size, type of AIS patients and task used. Study designs were adequate and demographics were reported. Eleven studies compared muscle activity between groups and 16 compared differences in convex-concave side between AIS and controls. Limited level of evidence was observed for higher heterolateral to homolateral activity ratios during side bending tasks, and higher bilateral activation duration in erector spinae in scoliosis than controls. In contrast, limited evidence exists of no differences in the amount of asymmetry in normalized activity during submaximal isometric contraction in extension at different intensities. Limited evidence supports weaker abdominal muscles, weakness in erector spinae muscle compared to controls during bilateral and unilateral movements, and different patterns of asymmetry during isokinetic contractions, which are more important in larger curves.

**Conclusion and significance**

Interestingly, despite exercises consistent with targeting endurance, there were no EMG studies on fatigability in AIS. We also found no studies using Transcranial Magnetic Stimulation to quantify central nervous system role in the torso muscle activity of AIS patients. Ultrasound and MRI studies of torso muscles were rarely done. Patients with AIS had higher EMG asymmetries than control, and higher activity on the convex side of the curve, specifically in patients with progressive curves.

## O47 Does back trunk asymmetry change in children and adolescents in forward flexion and standing erect position? A study using the scoliometer and 4d formetrics

### Emmanouil G. Maragkoudakis^1^, Theodoros B. Grivas^2^, Ioannis D.Gelalis^3^, Christina Mazioti^2^, Gerasimos Tsilimidos^2^, R. Geoffrey Burwell^4^

#### ^1^Private Practice, Athens, Greece; ^2^"Tzaneio" General Hospital of Piraeus, Piraeus, Greece; ^3^University Hospital of Ioannina, Ioannina, Greece; ^4^Nottingham University Hospitals Trust, Nottingham, UK

##### **Correspondence:** Theodoros B. Grivas

**Introduction**

The purpose of this study is to evaluate the effects of the forward bending (FB) test versus the standing erect (SE) position on back trunk asymmetry (TA).

**Objectives**

The Scoliometer readings in FB and the 4D Formetrics (4DF) readings in SE positions were assessed.

**Methods**

The measurements: The angle of trunk inclination (ATI) was measured in midthoracic, thoracolumbar and lumbar levels using the Scoliometer in the FB and the 4DF in SE position. The examined subjects: 134 subjects attending the Scoliosis Clinic (86 girls and 48 boys) aged from 7 to 18 years were assessed. The children and adolescents were divided in three groups according to the severity of TA, symmetric group 1 (0-2 degrees), asymmetry group 2 (2 to 6 degrees) and group 3 having asymmetry of 7 or more degrees. Children with leg length discrepancy were excluded from the study.

Statistics. The IBM SPSS v.20 package was used.

**Results and discussion**

The mean frequency of (group 1) symmetric boys and girls (0-2 degrees) was 37% and 63% respectively using the Scoliometer and 29% - 72% using 4DF. The mean frequency of (group 2) asymmetry 2-6 degrees for boys was 38% and for girls 62% using the Scoliometer and 35% for boys and 65% for girls using 4DF. The mean frequency of (group 3) asymmetry of 7 degrees or more was 23% for boys and 77% for girls using the Scoliometer and 37% for boys and 63% for girls using 4DF. At the midthoracic level comparing the Scoliometer to 4DF readings in males in group 1 the Wilcoxon Signed Ranks Test *p* = 0.451 while the Spearman's Rho = -0.138, in group 2 *p* = 0.184 and Rho = 0.204, in group 3 *p* = 0.109 and Rho =0.500. For females in group 1 *p* = 0.000 while Rho = 0.003, in group 2 *p* = 0.008 and Rho = 0.000, in group 3 *p* = 0.003 while Rho = 0.642. At the thoracolumbar level in males for group 1 *p* = 0.004 and Rho = -0.517, in group 2 *p* = 0.006 and Rho = 0.000, in group 3 *p* = 0.043 while the Spearman's Rho = 0.053. For females in group 1 *p* = 0.000 and Rho = -0.095, in group 2 *p* = 0.000 and Rho = -0.171, in group 3 p = 0.001 while Rho = -0.081. At the lumbar level for males in group 1 *p* = 0.000 while Rho = 0.149, in group 2 the *p* = 0.003 and Rho = 0.373, in group 3 *p* = 0.109 and Rho = (-). For females in group 1 *p* = 0.000 while Rho = -0.072 in group 2 *p* = 0.001 and Rho = 0.168 in group 3 *p* = 0.068 while Rho = 0.500. In all three anatomical regions of the spine, in both males and females, the change from a FB position to a SE position, shows a reduction of the mean asymmetry.

**Conclusion and significance**

The results of this study show that the back TA in children and adolescents is not similar in FB and SE position. This phenomenon probably is attributed to the complicated trunkal (spinal, thoracic and pelvic) anatomy and the results of this study may be used as a useful foundation for further understanding of torso dynamics.

## O48 Prevalence of idiopathic adolescent scoliosis among primary and middle school students in Wuxi, China

### Yu Zheng^1,2,3,4^, Xiao-Jun Wu^4^, Yi-Ni Dang^5^, Ning Sun^4^, Yan Yang^4^, Tao Wang^4^, Cheng-Qi He^2^, Man-Sang Wong^1^

#### ^1^Interdisciplinary Division of Biomedical Engineering, The Hong Kong Polytechnic University, Hong Kong, China; ^2^Department of Rehabilitation Medicine, West China Hospital, Sichuan University, Chengdu, Sichuan, China; ^3^Institute for Disaster Management and Reconstruction, Sichuan University-Hong Kong Polytechnic University, Chengdu, Sichuan, China; ^4^Department of Rehabilitation Medicine, Wuxi Rehabilitation Hospital, Wuxi, Jiangsu, China; ^5^Department of Gastroenterology, The First Affiliated Hospital of Nanjing Medical University, Nanjing, Jiangsu, China

##### **Correspondence:** Yu Zheng

**Introduction**

Adolescent idiopathic scoliosis (AIS) develops at the age of 10-16 years and is characterized by lateral curvature ^3^10¡ combined with vertebral rotation. It is not possible to find a specific disease causing the deformity. School-based AIS screening is an effective method for early detection and management. Based on this understanding, many studies on prevalence of scoliosis were conducted in Mainland China. Nonetheless, there is no study that reports the prevalence of AIS in the Wuxi population.

**Objectives**

The objectives of this study were to better understand the epidemiology of AIS in China based on a representative subject sample from Wuxi and to develop an efficient strategy for future screening in China.

**Methods**

From Jul. 2014 to Nov. 2015, students aged 10 to 16 years from most schools in Wuxi were screened. Initially, physical examination and Adam's forward bending test were performed. Students who had an angle of trunk inclination (ATI) of ^3^5¡ (determined using a Scoliometer) were referred to Wuxi Rehabilitation Hospital for whole spine X-ray examination. Their diagnosis was confirmed with Cobb angles of ^3^10¡. Data reflecting demographics and clinical items including curve type and level, Cobb angle and Risser sign were collected. Overall prevalence as well as prevalence by gender and age group were estimated. Multivariate logistic regression of diagnostic status on demographic and clinical items were eventually performed. Unit-non response (absent from X-ray examination although screened positive) was adjusted for by propensity score modeling. All results were provided with weighted and unweighted data.

**Results and discussion**

Totally, 86,145 students (46,669 boys and 39,476 girls) were screened in this study. Around 4% were screened positive including 1,120 boys (34.5%) and 2,125 girls (65.5%). The average BMI was 18.63 ± 2.65 (kg/m2). Body fat and resting metabolism were 20.56 ± 5.13 (%) and 1469.83 ± 249.32 (kcal), respectively. Average ATI degree evaluated with Scoliometer was 6.4 ± 1.9. The number of screened positive increased by age and was highest in 14-15 yr olds. Inversely, frequency decreased by ATI degree both in boys and girls. For the absolute agreement on ATI degree, inter-rater correlation coefficient (ICC) was 0.86 which is considered very high. The characteristics of respondents and non-respondents as well as weighted and unweighted overall prevalence will be presented later as the hospital-based diagnosis by X-ray examination is still in process and the referral rate now is around 50% which is expected to be at least 70% before the commence of the IRSSD-SOSORT 2016. Detailed epidemiological results will be reported by then.

**Conclusion and significance**

Based on this representative sample, it is able to estimate the prevalence of AIS in Wuxi city and explore its representative in China. The results would serve to target the screening at a population with high risk of AIS. Moreover, the application of propensity score model in this study would reduce bias launched by non-respondents.

## O49 Compliance monitor usefulness is confirmed by patients with AIS and their parents who used it: results from a survey

### Sabrina Donzelli^1^, Gregorio Martinez^1^, Alberto Negrini^1^, Fabio Zaina^1^, Stefano Negrini^1,2,3^

#### ^1^ISICO Italian Scientific Spine Institute Milan Italy; ^2^University of Brescia, Brescia, Italy; ^3^Don Gnocchi Foundation, Milan, Italy

##### **Correspondence:** Sabrina Donzelli

**Introduction**

A temperature monitor, can be used to objectively measure the hours of brace wear. Since 2010 we introduced this tool, that we called Thermobrace (TB), as a standard of care in everyday clinical practice. Through TB we documented a compliance dramatically higher than previously reported (91.7% of prescription; IC95 56.6-101.7%). After this five-year experience, when braced patients do not accept the use of sensors, treatment becomes more difficult and less accurate. Attitude towards this electronic device have never been investigated.

**Objectives**

The present study aims to investigate the attitude of parents and patients towards the use of temperature sensor for measuring brace wear compliance, in the following domain: understanding of the device, usefulness, acceptance, reliability, and feeling related to the moment of data reading.

**Methods**

We required participation of a sample of 301 consecutive girls and 63 boys and their parents, mean age 14,65 (SD 2.36) mean Cobb angle 34,18 (SD 13,57), average prescription was 21,76 hours per day (SD 2,53). The population was selected according to the following inclusion criteria: brace wear fulltime prescription at first visit and at least one visit with download and discussion of TB data. The main domain of the questionnaire regarded: usefulness, acceptability, reliability, feeling related to the moment of data download, reading and discussion. All these item were investigated in parents and patients. A first draft of the questionnaire was prepared by two expert physicians (SD and SN), after a pilot study involving 10 patients, a final version of the questionnaire was defined. Patients were invited to complete the questionnaire anonymously by the administrative staff.

**Results and discussion**

Among the recruited subjects we collected 336 questionnaires (rate of responders: 92.3%). Patients stating that TB is useful were the 74%, and 88% parents. The 64.5% of patients consider the TB at least in part an ally and helpful in improving their adherence to prescription; among parents the percentage was similar: 63%. The 68% of parents think it's useful to know that their children are monitored during therapy and the 86% declare that there is not any affection of the trust for their children. Sharing data with their physician or their parents, never disturbed patients in the 87% of cases. The 91% of parents would use TB again and would recommend it to other people in the 89% of cases.

**Conclusion and significance**

This is the first study investigating the attitude of parents and patients towards brace wear compliance monitor. People who experienced these objective monitoring is aware of the advantages related to it, and support its usefulness not only for clinicians, but also for patients and parents without affecting the children and parents, neither the patients-physician relation-ship. The present results should encourage the spread of these tools in daily clinical practice.

## O50 Patients undergoing casting for early onset scoliosis experience decreases in short-term health related quality of life

### Hiroko Matsumoto^1^, Nicholas Feinberg^1^, Matthew Shirley^1^, Hasani Swindell^1^, Zachary Bloom^1^, David P. Roye^1^, Behrooz A. Akbarnia^2^, Sumeet Garg^3^, James O. Sanders^4^, David L. Skaggs^5^, John T. Smith^6^, Michael G. Vitale^1^, Children's Spine Study Group^7^, Growing Spine Study Group^8^

#### ^1^Columbia University Medical Center/Morgan Stanley Children's Hospital, New York, NY, USA; ^2^Rady Children's Hospital, San Diego, CA, USA; ^3^Children's Hospital Colorado, Aurora, CO, USA; ^4^University of Rochester, Rochester, NY, USA; ^5^Children's Hospital of Los Angeles, Los Angeles, CA, USA; ^6^University of Utah, Salt Lake City, UT, USA; ^7^Growing Spine Foundation, San Diego, CA, USA; ^8^Children's Spine Foundation, Valley Forge, PA, USA

##### **Correspondence:** Michael G. Vitale

**Introduction**

The primary goal of treatment for Early Onset Scoliosis (EOS) is to improve on the natural history of the disease, including poor pulmonary function and low quality of life, by stopping curve progression. In order to provide the best care we must understand not just the efficacy, but also the impact on health-related quality of life (HRQoL) of the treatments we deploy in combatting EOS.

**Objectives**

The purpose of this study is to describe the changes HRQoL and Family Burden of patients with EOS who underwent treatment with surgery or casting.

**Methods**

We conducted a retrospective cohort study recording the HRQoL and Family Burden of patients with EOS treated with surgery or serial casting. Surgical treatment included Vertical Expandable Prosthetic Titanium Rib and Growing Rod instrumentation. Seventeen surgical patients presented with post-operative complications: 12 non-idiopathic patients had minor complications (complications not necessitating re-operation) and 3 had major complications (complications necessitating re-operation); the idiopathic patients had 2 minor complications. The Early Onset Scoliosis 24-Item Questionnaire (EOSQ-24) was administered to caregivers of patients prior to treatment and at 6 months to 1 year after treatment. Included were 8 idiopathic and 49 non-idiopathic surgically-treated patients, and 14 idiopathic and 9 non-idiopathic casting patients. Student’s T-test was used to analyze the difference in EOSQ-24 responses.

**Results and discussion**

In surgically-treated patients with idiopathic EOS most HRQoL categories remained unchanged as did Family Burden. A marked improvement in parental impression of patient pulmonary function was observed. In addition, a marked decline in energy level was seen. Casting patients with idiopathic EOS had post-treatment declines in transfer (patient mobility), daily living, physical function, and emotion categories, with the other HRQoL domains and Family Burden staying unchanged. In surgically-treated patients with non-idiopathic EOS, parental impression of patient pulmonary function improved while the remaining HRQoL categories and Family Burden were unchanged. Casting patients with non-idiopathic EOS showed a marked decline in the emotion domain. All other measurements were unchanged.

**Conclusion and significance**

Patients treated surgically had an improved parental impression of patient pulmonary function. Idiopathic surgery patients had a decline in energy level. By contrast, casting appears to be associated with decreasing HRQoL scores in multiple domains.

This study describes the short-term trajectory of patient experience and should help us more adequately inform patients regarding their course of care.

## O51 Does casting with window cut affect biomechanical strength in the treatment of children with scoliosis?

### Robert Rizza^1^, XueCheng Liu^2^, John Thometz^2^

#### ^1^Department of Mechanical Engineering, Milwaukee School of Engineering, Milwaukee, WI, USA; ^2^Department of Orthopaedic Surgery, Medical College of Wisconsin, Milwaukee, WI, USA

##### **Correspondence:** Robert Rizza

**Introduction**

Serial casting has been broadly recognized as an effective conservative treatment for children with early-onset scoliosis (EOS). The casting technique that was described by Dr. Mehta requires cutting anterior and posterior windows in order to reduce pulmonary complications and facilitate derotational anomalies of the rib hump. However, there are concerns with the dimensions of window in casting, which may have detrimental impact on corrective strength provided by the casting material's properties.

**Objectives**

This study was motivated by goal to compare the effect of cut-outs on the mechanical performance of a cast brace.

**Methods**

A finite element analysis (FEA) of a patient's brace was created using an optical scan of the brace. Two basic designs were considered. In the first design a "front" cut-out located anteriorly on the abdominal region of the brace was considered. In the second design, a lateral-posterior ("side") cut-out was considered. The number of layers in the cast model was varied to determine the number of optimal layers. The direction of the reinforcing fibers ("schedule") was varied. Nine different schedules were considered for each cast brace design to determine the optimal design. The effect of the various schedules, number of layers and cut-outs on the lateral deflection was determined and compared to a cast brace with no cut-outs. Two different cast materials (Delta-Cast Soft® and 3 M® ScotchCast™ Plus Casting Tape) were used in the analysis.

**Results and discussion**

The simulations indicate that the no-cut-out cast brace with 6 layers will generate at most 4.7 mm of deformation. All simulations with the different schedules for the "side" cut-out showed a maximum deformation of 4.8 mm for Delta-Cast Soft®. The deformation ranged from 4.2 mm to 5.0 mm for the 3 M ScotchCast™. Interestingly, the brace may be designed by varying the schedule so the lateral deflection is even less than the solid cast. For the "front" cut-out the deformation ranged from 5.5 mm to 4.1 mm depending on the schedule for the Delta-Cast Soft® and 4.9 mm to 5.9 mm for the 3 M ScotchCast™.

**Conclusion and significance**

With Delta-Cast Soft® there is no effect on the brace performance for a "side" cut-out. For 3 M ScotchCast™, there is more of an effect but it is minimal. In the case of the "Front" cut-out, the schedule plays an important role in the structural performance of the brace and there is a much more significant difference between the solid brace and the brace with the cut-out, irrespective of whether the brace is made of Delta-Cast Soft® or 3 M ScotchCast™. Thus, when a "front" cut-out is used in a cast brace, the schedule must be properly chosen to ensure that the brace supports the biomechanical loads applied to the spine.

## O52 Results of ultrasound-assisted brace casting for adolescent idiopathic scoliosis

### Edmond Lou^1,2^, Doug Hill^2^, Andreas Donauer^2^, Melissa Tilburn^2^, Douglas Hedden^1^, Marc Moreau^1^

#### ^1^University of Alberta, Edmonton, Canada; ^2^Alberta Health Services, Edmonton, Canada

##### **Correspondence:** Edmond Lou

**Introduction**

In standard practice, in-brace correction is assessed during the first follow-up clinic approximately 6 weeks after the brace has been fabricated. Unsatisfactory correction may trigger adjustments to the brace which will delay the effective treatment.

**Objectives**

This study assessed the benefits of using real-time ultrasound (US) to assist brace casting (intervention) versus the standard brace design method (control) (NCT02996643).

**Methods**

Thirty-four full time TLSO candidates with AIS participated with 17 (2 M, 15 F; Major Cobb: 35 ± 8 degrees; 12.9 ± 1.6 years) in the intervention group and 17 (2 M, 15 F; Major Cobb: 32 ± 9 degrees; 13.2 ± 1.4 years) in the control group. A medical ultrasound (US) system integrated with position sensing was used to acquire real-time spinal images. A custom Providence brace design system, pressure adjustment system and measurement software were used to provide real time feedback to the orthotist. The ultrasound assisted method has been described in a previous study. Participants were assessed during their first in-brace follow-up clinic.

**Results and discussion**

For the US group, only 1 subject (6%) required adjustment. The average in-brace correction at the first in-brace follow-up clinic was 48 ± 17%. For the standard brace design group, 8 of 17 (47%) subjects required brace adjustments. The average in-brace correction at the initial in-brace clinics was 40 ± 20%. Among the subjects requiring adjustment, 3 (38%) required a second adjustment. The *p*-value of the Chi-square for requiring brace adjustment was 0.0065 which is a statistically significant difference between the two groups. The *p*-value of the unpaired Student t-test of the in-brace correction between the two groups was 0.35 which shows no statistically significant difference. The reduction of the number of in-brace radiographs was large, 18 in-brace radiographs from the US group versus 28 in-brace radiographs from the control group, a saving of 10 radiographs in 17 subjects.

**Conclusion and significance**

The ultrasound system provided a radiation-free method which reduced the need for brace adjustments, resulting in decreased radiation exposure and quicker time to effective brace treatment.

## O53 Effectiveness and cost effectiveness of orthotic interventions in scoliosis: A systematic review

### Aoife Healy, Sybil Farmer, Nachiappan Chockalingam

#### Faculty of Health Sciences, Staffordshire University, Stoke-on-Trent, United Kingdom

##### **Correspondence:** Nachiappan Chockalingam

**Introduction**

There has been an increased amount of research on the orthotic interventions in the clinical management of spinal deformities and scoliosis in the past few years. Given the variety in the types of interventions, there is a lack of structured information on the effectiveness and cost effectiveness of these interventions. In addition, little is known about the types of materials and effectiveness of the outcome measures used within these studies.

**Objective**

The main objective of this review is to see if we can establish the effectiveness and cost effectiveness of orthotic intervention in scoliosis and spinal deformities.

**Methods**

The following 14 databases were searched: Web of Science, Medline, PubMed, CINAHL Plus, EMBASE, SCOPUS, Rehabdata, PsycInfo, ERIC, Education Research Complete, Business Source Complete, IEEE, NIHR and CEA Registry. MeSH headings and free text terms for orthotics were used along with study design categories to capture all research in the area of orthotics and spinal deformities. No language restriction was applied to the search. Searches were adapted for each database and were completed during September 2015. Initial screening was conducted by two reviewers. Two reviewers then independently screened the titles and abstracts of these studies for full text review. The independent screening was completed using the online screening software provided by Covidence (https://www.covidence.org/), with discrepancies resolved through discussion.

**Results and discussion**

This review provides a clear outline of the types of braces and other orthotic interventions used within the management of scoliosis and other spinal deformities. The results highlight main regions of the world where these studies are being conducted and the lack of quantitative outcome measures used within these studies. More importantly, this review discusses the quality of these studies and proposals for improving these studies.

**Conclusions and significance**

Whilst there is some evidence for the effectiveness of orthotic intervention in scoliosis and spinal deformities, there is still a paucity of structured high quality research which clearly provides cost effectiveness of these interventions.

## O54 Is the Risser test valuable to determine the start of weaning from the brace in the conservative treatment of idiopathic scoliosis

### Angelo Gabriele Aulisa^1^, Vincenzo Guzzanti^1^, Marco Galli^2^, Paolo Pizzetti^3^, Lorenzo Aulisa^2^

#### ^1^U.O.C. of Orthopedics and Traumatology, Children's Hospital Bambino Gesù, Institute of Scientific Research, P.zza S. Onofrio 4, Rome, 00165, Italy; ^2^Department of Orthopedics, University Hospital "Agostino Gemelli", Catholic University of the Sacred Heart School of Medicine, Rome, 00168, Italy; ^3^Independent practitioner, Rome, Italy

##### **Correspondence:** Angelo Gabriele Aulisa

**Introduction**

All authors agree that the brace treatment should be continued for the entire period of skeletal growth. In fact, to achieve remodeling of the motion segments, the mechanical action of the brace should act for all the period during the vertebral cartilages are active. However, it should be underlined that the stage of skeletal maturity is determined by indirect indicators, such as the Risser sign and/or ossification of the ring-vertebral apophyses, whose correlation with chronological age and end of spinal growth, is highly variable among individuals.

**Objectives**

The aim of the study is to determine the role of the Risser sign in the conservative treatment presenting the results of bracing patients that start the treatment with Risser 4 and them that have abandoned the brace at Risser 4.

**Methods**

The study includes two groups of patients, (who had their first evaluation in our institute, between 1999 and 2014). Group A is 39 patients (10 males) all AIS with Risser 4 at the beginning of treatment, Group B is 20 patients (1 male) all AIS which have abandoned the brace at Risser 4. All Patients had full-time brace prescription (22 hours/day), weaning in Group A was started at complete fusion of ring apophysis and SOSORT management criteria were respected. The follow-up from the end of treatment was 24 months. Antero-posterior radiographs were used to estimate the curve magnitude (CM) and the torsion of the apical vertebra (TA) at: beginning of treatment (t1), at time of abandoned (t3) and 2-year minimum follow-up (t5). Three outcomes were distinguished in agreement with SRS criteria. Statistical analyses were performed with GraphPad Prism 6.

**Results and discussion**

The results of our study showed that the CM mean value of Group A was 33.6 ± 8.9 SD at t1 and 24.55 ± 10.7 SD at t5. TA was 15.89 ± 5.5 SD at t1 and 13.2 ± 6.7 at t5 instead of Group B was 33.1 ± 11.08 SD at t1, 19.60 ± 9.11 SD at t3 and 28.35 ± 9.4 SD at t5. TA was 15.4 ± 4.2 SD at t1 and 11.2 ± 3.9 at t5. The variations between measures of Cobb and Perdriolle degrees between CM t5-t1 and TA t5-t1 were statistically significantly different for both groups (*p* < 0.0001). Matched results, from Group A and B showed that at baseline, no differences were noted for Cobb angle (33.6 ± 8.9 vs 33.1 ± 11.08) or Perdriolle (15.89 ± 5.5 vs 15.4 ± 4.2) instead the correction at follow up was slightly better for the group A, but didn't reach statistical significance (24.55 ± 10.7 vs 28.35 ± 9.4). No differences were noted for Curve correction (79.5% vs 80%) and curve progression (2.5% vs 5%).

**Conclusion and significance**

Our results show that patients who started treatment at Risser 4 had a correction slightly better than those who have abandoned at Risser 4. This occurs because the growth of the spine ends to the fusion of ring apophysis.

In conclusion, to achieve better results, the weaning from the brace must start at complete ring apophysis fusion.

## O55 Indication of brace treatment for adolescent idiopathic scoliosis patients with Risser III or IV

### Toru Maruyama^1^, Yosuke Kobayashi^2^, Yusuke Nakao^2^

#### ^1^Saitama Prefectural Rehabilitation Center, Ageo, Japan; ^2^Saitama Medical Center, Saitama Medical University, Moroyama, Japan

##### **Correspondence:** Toru Maruyama

**Introduction**

In 2013, BrAIST study demonstrated the effectiveness of brace treatment for adolescent idiopathic scoliosis (AIS) patients with Risser 0-II. Also Scoliosis Research Society (SRS) brace study standardization criteria include AIS patients with Risser 0-II. Originally these criteria have been proposed to evaluate the treatment results of more progressive curves to facilitate demonstration the effectiveness of bracing or comparison among studies. However, these criteria are likely to be misunderstood as the indication of bracing and sometimes patients with Risser sign of III or IV are considered as out of the indication of brace treatment.

**Objectives**

To analyze outcomes of brace treatment for AIS patients with Risser sign of III or IV and to clarify its indication for them.

**Methods**

AIS patients with age over 10 years, Risser sign of III or IV, Cobb angle of 25 to 40 degrees before treatment and underwent no prior treatment were included. At the final follow-up after the patients reached Risser sign of V, the radiological outcomes and the rate of the patients who underwent or who were recommended surgery were investigated according to the SRS brace study standardization criteria.

**Results and discussion**

A total of 17 female patients were included in the analysis. The average age was 12.9 years (11-14) and the average Cobb angle was 32.9 degrees (25 to 40) before treatment. Risser sign was III in five and IV in 12 patients. There were seven thoracic, five thoracolumbar, and five double major curves. Initial correction rate by the brace was 38.8%. After an average follow-up period of 39 months, the average Cobb angle changed to 32.5 degrees. Two patients improved in more than 6 degrees. Changes of 15 patients were within 6 degrees. No patients progressed in more than 6 degrees, so all the curves were stabilized by the treatment. No patients exceeded 45 degrees nor underwent surgery. These results were better than those of the patients with Risser 0 to II treated in our institution or the reported natural history.

**Conclusion and significance**

100% of the AIS curve with Risser III or IV could be stabilized by the brace treatment. Majority of the curves were unchanged, but some curves were improved by the treatment. Brace treatment is indicated for AIS patients even with Risser III or IV.

## O56 Does disc wedging contribute to the effects of brace treatment in adolescent idiopathic scoliosis?

### Hao Liu, Bang Ping Qian, Yong Qiu, Sai-hu Mao, Bin Wang, Yang Yu, Zezhang Zhu

#### Spine Surgery, the Affiliated Drum Tower Hospital of Nanjing University Medical School, Nanjing, China

##### **Correspondence:** Bang Ping Qian

**Introduction**

Wedging of the scoliotic inter-vertebral disc (IVD) was previously reported as a contributory factor for progression of idiopathic scoliotic (IS) curves. And initial disc wedging percentage was proposed to serve as a new prognostic factor of curve progression in AIS. However, it remains unknown whether there is a link between the initial disc wedging percentage and the outcome of brace treatment.

**Objectives**

To investigate whether the initial disc wedging percentage could serve as an important factor in predicting the curve behavior, regression, or stabilization, after the treatment of bracing in adolescent idiopathic scoliosis (AIS).

**Methods**

A retrospective review was performed on patients with AIS who received standardized bracing treatment at our clinic from January 2009 to July 2014. Standardized SRS criteria for bracing study were utilized in the case selection. The demographic data, growth status, and Cobb angle of each visit were recorded. The initial disc wedging percentage was identified. Patients were divided into progressive (group A, ≥6^0^) and non-progressive (group B, <6^0^) groups based on their final bracing outcome. Differences between two groups were identified and the associations between the bracing outcome and the indices before bracing, including anthropometric measurements (BMI), curve magnitude, and disc wedging percentage were evaluated using univariate analysis and regression analysis.

**Results and discussion**

Sixty-one patients were included. There were 15 (24.59%) girls in group A and 46 (75.41%) girls in group B, respectively. Significant differences between progressive and non-progressive groups were found in terms of BMI (17.4 ± 2.6 vs. 15.3 ± 3.2, *P* < 0.05), initial disc wedging percentage (31.7% ± 18.3% vs. 63.4% ± 22.4%, *P* = 0.001), and Cobb angle (29.4^0^ ± 6.7^0^ vs. 25.5^0^ ± 5.6^0^, *P* < 0.05). As revealed by the multiple logistic regression analysis, initial disc wedging percentage (*P* = 0.001) was identified as an independent risk factor in curve progression in AIS girls, during the total duration of bracing treatment.

**Conclusion and significance**

The initial disc wedging percentage is identified to serve as a new independent risk factor in the curve progression during the duration of bracing treatment. The evaluation of initial disc wedging percentage before bracing may help to predict the effects of brace treatment.

## O57 Lenke, Suk, Rigo classification systems: how do they compare?

### Hagit Berdishevsky, Amelia M. Lindgren, Prachi Bakarania, Kelly Grimes, Melvin C. Makhni, Jamal Shillingford, Michael G. Vitale

#### Columbia University Medical Center/Morgan Stanley Children's Hospital, New York, NY, USA

##### **Correspondence:** Hagit Berdishevsky

**Introduction**

Adolescent Idiopathic Scoliosis (AIS) is a 3-D deformity of the spine. Several classification systems have been developed to delineate appropriate treatment. The most well known system is the Lenke system, which aims to be a comprehensive classification with 42 possible curves. The Suk system only has 8 curve types, while Rigo has 16.

**Objectives**

To review the Lenke, Suk and Rigo classification systems, classify 100 AIS patients by all three systems and investigate the correlations between the different systems.

**Methods**

100 consecutive AIS patients were identified who underwent spinal fusion surgery between 11/2009-6/2012. Pre-operative Scoliosis X-Ray series, including coronal, sagittal, left and right bending films, were utilized for the classification of each patient. The X-Ray series were reviewed by one individual and classified separately by the Lenke, Suk and Rigo systems.

**Results and discussion**

Seventy eight were female and 22 were male, with an average age of 14.7 years old (10-23). Lenke: there were 48 Type1 curves, 19 Type2, 7 Type3, 3 Type4, 11 Type5 and 12 Type6 curves. The most common Lenke classifications were 1AN, 1CN, 2AN, 6CN and 5CN. Suk: there were 47 ST curves, 20 DT, 28 DM and 5 TL curves. The most common Suk classifications were: STA, DMB, and STB. Rigo: included 31 A, 47 B, 17 C and 5 E curves. 15 cases contained a proximal thoracic D modifier. The most common classifications were: B1, A1, B2, C2 and A3. Classification Comparisons: Within the Type1 Lenke curves, 33% were classified as single thoracic curves by Rigo (A1, A2 or C1). 62.5% fell into double curve categories (A3, B1, B2 or C2). 79% of Type1 were also classified as Suk ST. Type2 Lenke curves were found to have a D proximal thoracic Rigo modifier 58% of the time; the remainder of the cases did not have significant proximal thoracic rotation to qualify for the D modifier. Similarly, 74% were classified as Suk DT. Both Lenke Type3 and Type6 are double major curves depending on which curve is larger, and all cases were classified as Rigo double curves. Specifically, 58% were B1, 37% were B2. Lenke3 was split between Suk ST (57%) and DM (43%), depending on curve size. Lenke6 was entirely consistent with Suk DM. Type5 Lenke curves fell into both Rigo E/Suk TL (45.4%), as well as RigoB1 curves/Suk DM (54.5%) where the thoracic curve bent out.

**Conclusion and significance**

These 3 systems were designed for distinct purposes and did not correlate perfectly. Although there are core areas of overlap, there are inherent differences that make it difficult to translate curve types between systems. Each system prioritizes different curve characteristics. Although both Lenke and Suk are geared towards optimal surgical planning, Lenke focuses on flexibility while Suk focuses more on rotation. Rigo is geared towards brace design and is more appropriate for smaller curves. Future research efforts should focus on the relative success of bracing of various Rigo curve subtypes and the natural history of evolution into curve patterns as described by Suk and Lenke.

## O58 Exploring the impact of physiotherapy scoliosis specific exercise (PSSE) based therapy on idiopathic scoliosis using SRS-30 and health economic measures: a provisional analysis

### Jason Black^1^, Erika Maude^1^, Abbie Turland^1^, David Glynn^2^

#### ^1^Scoliosis SOS, London, United Kingdom; ^2^Independant, York, United Kingdom

##### **Correspondence:** Jason Black

**Introduction**

A key objective of research in scoliosis should be to demonstrate value. Health economics assesses the value of treatment to health systems and is increasingly used to inform funding decisions. To measure value across disease area, generic quality of life measures such as the EQ5D are used. The dimensions of this measure are mobility, self-care, usual activities, pain and anxiety/depression. These 5 dimensions have 5 levels ranging from no problems to extreme problems. These dimensions are weighted to reflect trade-offs in quality and quantity of life using values from the UK public and are used to calculate quality adjusted life years (QALYs). As the SRS Questionnaires are used in a number of scoliosis studies, understanding how these are related to the EQ5D and how scoliosis therapies such as ScolioGold affect economic measures is an important aspect of demonstrating value to health systems.

**Objectives**

This study aims to understand how SRS-30 and EQ5D5L are related and estimate the magnitude of benefit from ScolioGold exercise based therapy.

**Methods**

Participants presenting at the Scoliosis SOS clinic filled out both SRS-30 and EQ5D5L questionnaires before and after treatment with ScolioGold method. Preliminary analysis of the effect of the treatment and the association between the measures are carried out using statistical methods.

**Results and discussion**

One hundred sixty-one consecutive patients were registered on the database as of 27/11/2015. Of this number 156 (97%) provided pre-treatment data and 110 (68%) provided both pre and post treatment data. Before treatment mean SRS-30 score was 3.57 ranging from 1.86 to 4.90 and mean EQ5D score was 0.76 ranging from 0.22 to 1, *n* = 156. The mean change after treatment for EQ5D was an improvement of 0.09 (95% CI 0.055 to 0.114) for the SRS-30 this was 0.24 (95% CI -0.148 to 0.332), *n* = 110. Both were statistically significant improvements with *p* < 0.01 for both measures. The relationship between SRS-30 and EQ5D scores was estimated to allow researchers to predict EQ5D scores from SRS-30 data. The model estimated has an intercept of -0.029 (SE 0.043) and the following coefficients for function: 0.073 (SE 0.016), pain: 0.067 (SE 0.014) and mental health: 0.063 (SE 0.011). Self-image and satisfaction with care were not statistically related to EQ5D so were dropped from the model. The model was based on 156 observations and explains 71% of the variation in EQ5D. The only baseline variable statistically related to EQ5D or SRS-30 was age with higher age associated with lower scores (*p* < 0.01 for both). The spinal fusion classification was weakly associated with SRS score (*p* = 0.09).

**Conclusion and significance**

The ScolioGold method is associated with significant improvements in both SRS-30 and EQ5D. The relationships estimated here can be used to map between SRS-30 and EQ5D allowing researchers to predict EQ5D scores from SRS-30 data. Subject recruitment continues and data at 6 months and 12 months is being collected for further analysis to investigate longer term effects.

## O59 ISYQOL: a Rasch consistent questionnaire for measuring health related quality of life in adolescents with spinal deformities

### Antonio Caronni^1^, Luciana Sciumè^2^, Sabrina Donzelli^3^, Fabio Zaina^3^, Stefano Negrini^3,4,5^

#### ^1^Department of Neurorehabilitation Sciences, Casa di Cura del Policlinico, Milan, Italy; ^2^University of Milan, Milan, Italy; ^3^ISICO Italian Scientific Spine Institute, Milan, Italy; ^4^University of Bresciam, Brescia, Italy; ^5^Don Gnocchi Foundation, Milan, Italy

##### **Correspondence:** Sabrina Donzelli

**Introduction**

Spinal deformities are usually associated with poor quality of life (QoL). Several questionnaires have been developed to evaluate QoL in idiopathic scoliosis (the SRS-24, SRS-22, the SQLI and the EOSQ for early onset scoliosis), and some questionnaires have been developed to measure specific dimension of the spinal deformity, like the body image (SAQ) the impact of brace like the BSSQ and BrQ questionnaires. Rasch analysis is a statistical methodology to develop good quality QoL questionnaire, able to make them interval instead of ordinal measures with obvious clinical and research advantages. Only the SRS -22, has been evaluated with Rasch analysis, but showed poor clinimetric properties after Rasch analysis application.

**Objectives**

The main aim of the present research is to develop a new questionnaire (ISYQOL: Italian Spine Youth Quality of Life questionnaire) able to satisfy the fundamental requirement for Rasch analysis.

**Methods**

A multistage classical methodology has been used, including: - A content analysis of the messages posted by adolescents with spinal deformities in an internet forum specifically developed in 2006 (containing 5758 visitors' posts and 1156 answers from expert clinicians), guided the items selection. - An opinion poll among 23 experts provided a first version of the questionnaire, starting from 50 possible items. - This first draft of the questionnaire, 50 questions, was tested in 94 patients. - A Rasch analysis guided the generation of a second version of the questionnaire (23 questions) that was tested again in 39 patients, to verify its validity. - A final study, with 402 participants who self-filled one of the two versions of the ISYQOL questionnaire in the waiting room, immediately before medical evaluation. Statistics: Rasch analysis was performed by using Winsteps Rasch Measurement software (2009, version 3.69.1; partial credit model).

**Results and discussion**

After Rasch analysis, 20 items fitted the model and constituted the final version of the ISYQOL questionnaire. Differential Item functioning was significant for brace (-0.87 vs -1.62 logit, respectively; *p* = 0.0015), thus allowing comparison among patients with and without brace. The principal component analysis on Rasch residual confirmed the unidimensionality of the ISYQOL The finding of an addition variable hidden in the ISYQOL Rasch residuals (1st factor Eigenvalue = 2.2) explains 5.2% of the total data variance. Participant reliability of ISYQOL is 0.83 and thus ~3 significantly different strata can be discerned in the sample population.

**Conclusion and significance**

The present work presents the Italian Spine Youth Quality Of Life (ISYQOL) questionnaire, the first questionnaire developed in the Rasch analysis to measure HRQOL in adolescents with an idiopathic spine deformity. Being Rasch consistent, ISYQOL offers an HRQOL measure which is additive, generalizable and unidimensional thus complying with requirement of a genuine continuous measure. ISYQOL can offer an insight on the impact of the brace prescription.

## O60 The effect of Schroth exercises added to the standard of care on the Cobb angle in adolescents with idiopathic scoliosis - an assessor and statistician blinded randomized controlled trial

### Sanja Schreiber^1^, Eric C. Parent^1^, Elham Khodayari Moez^1^, Douglas M. Hedden^1,2^, Douglas L. Hill^1,2^, Marc Moreau^1,2^, Edmond Lou^1^, Elise M. Watkins^1^, Sarah C. Southon^2^

#### ^1^University of Alberta, Edmonton, Canada; ^2^Alberta Health Services, Edmonton, Canada

##### **Correspondence:** Sanja Schreiber

**Introduction**

In America the non-surgical standard of care for adolescent idiopathic scoliosis (AIS) includes observation and bracing, but not exercises. Schroth scoliosis-specific exercises showed promise in several studies of sub-optimal methodology. The Scoliosis Research Society calls for rigorous research studies supporting the role of exercises before recommending them as a treatment for scoliosis.

**Objectives**

To determine the effect of a six-month Schroth intervention added to standard of care (Experimental group) on the Cobb angle compared to standard of care alone (Control group) in patients with AIS.

**Methods**

Fifty patients with AIS aged 10 -18 years, with curves of 10¡- 45¡ and Risser grade 0 -5 were recruited from a single pediatric scoliosis clinic in Canada and randomized to the Experimental or Control group (NCT01610908). The primary and secondary outcomes were the change in the Largest Curve and Sum of Curves from baseline to 6 months. The intervention consisted of a 30 – 45-minute long daily home program of Schroth exercises and weekly 1 h-long supervised sessions. The exercises, adapted to the curve types were taught over 5 individual sessions, and adjusted weekly during supervised sessions according to an algorithm. The Cobb angles for all curves were measured on digital radiographs using a semi-automated method with high reliability. Intention-to-treat (ITT) and per protocol linear mixed effects model analyses are reported.

**Results and discussion**

The mean Largest Curve and Sum of Curves were 28.5¡ (SD = 8.8¡) and 51.2¡ (SD = 22.3o). Risser sign was 1.60 (SD = 1.73). Schroth curve types were balanced between groups including: 3c (*n* = 7), 3cp (*n* = 15), 4c (*n* = 5) and 4cp (*n* = 23). Six/50 (12%) dropped out (4 in the Schroth and 2 in the Control group). Using ITT principle 76% of visits and 73% of the prescribed home exercises were completed. After six months the ITT showed that Schroth group had significantly smaller Largest Curve than controls (-3.5¡, 95%CI -1.1¡ to -5.9¡, *p* = 0.006). Likewise, the difference in the square root of the Sum of Curves was -0.40¡, (95%CI -0.03¡ to -0.8¡, *p* = 0.046), suggesting that an average patient with a 51.2¡ at baseline, will have a 49.3¡ Sum of Curves at six months in the Schroth group, and 55.1¡ in the control group with the difference between groups increasing with severity. Per protocol analyses produced even larger differences: Largest Curve (-4.1¡, 95%CI -1.7¡ to -6.5¡, *p* = 0.002) and SQRT [Sum of Curves] (-0.5¡, 95%CI -0.8 to 0.2, *p* = 0.006). Assuming that the patients with missing values had curve progression, more patients were successfully treated (improved + stable) in the Schroth than in the control group (88% vs. 60%, Chi2 *p* = 0.024).

**Conclusion and significance**

Schroth exercises added to the standard of care were superior compared to standard of care alone in reducing the curve severity in patients with AIS. Schroth exercises alone or in combination with brace management present an enhanced alternative to conservative standard of care in patients with curves <45¡.

## O61 Effects of Schroth exercises added to standard care in adolescents with idiopathic scoliosis (AIS) on surface topography parameters - a randomized controlled trial (RCT)

### Eric C. Parent^1^, Sanja Schreiber^1^, Elham Khodayari Moez^1^, Preston Sloan^1^, Douglass Hedden^1,2^, Marc Moreau^1,2^, Douglas Hill^1,2^, Sarah Southon^1,2^, Elise Watkins^1^

#### ^1^University of Alberta, Edmonton, Canada; ^2^Alberta Health Services, Edmonton, Canada

##### **Correspondence:** Eric C. Parent

**Introduction**

In studies of suboptimal quality, Schroth exercises have shown promise for slowing curve progression and improving posture in patients with AIS. However, few studies have reported quantitative measurements of the effect of scoliosis specific exercises on the external deformity. Reviews call for randomized and prospective controlled studies on exercises for scoliosis.

**Objectives**

This assessor and statistician-blinded RCT in patients with AIS (NCT01610908) aimed to determine the effect of Schroth exercises added to standard of care (EXP group) to standard of care alone (CTRL group) on the external deformity measured using surface topography.

**Methods**

Fifty consecutive participants with AIS, aged 10-18 years, with curves of 10°-45° and Risser 0-5, were recruited from a scoliosis clinic and randomized to the EXP or CTRL group. A Schroth home program adapted to each curve type was taught over five individual sessions, and adjusted weekly according to an algorithm during a therapy visit. Compliance with the home program and the quality of the performance of exercises was assessed weekly using a checklist. Postural measurements (10 frontal, 8 transverse, 3 sagittal and one combining planes) were recorded at baseline, 3 and 6 months using full torso surface topography laser scanners with a frame standardizing the positioning of the extremities. Reliable surface measurements were extracted using custom software following digitization of key landmarks by an evaluator blinded to group allocation. Linear mixed models were used to test differences between groups over time while adjusting for covariates.

**Results and discussion**

At baseline, despite lighter weight in the Schroth group, groups were similar for age [EXP 13.5 yrs (12.7-14.2) CTRL 13.3 yrs (12.7-13.9)], gender, height, use of a brace (17 per group), curve type (≤1 subject difference per type) and baseline Cobb angle [EXP: 29 degrees (95%CI 25-32) CTRL 27.9 degrees (24-32)]. For 10 of 22 variables analyzed, a statistically significant difference between groups was observed after 3 or 6 months in parameters reflecting deformity in the frontal (at 3 mths: Pelvic tilt; at 6 mths: Sternum vs C7S angle, waistline depth, and decompensation), transverse (at 3mths: back surface rotation; at 6 mths: Deformity in the Axial Plane Index, back depth), and sagittal (at 6 mths: kyphosis and lordosis angles) planes. The following covariates reached significance in between 1 and 5 analyses each indicating they have an influence on the effect of treatment: age, height, weight, bracing, curve type and self-efficacy (perception of ability to perform Schroth corrective exercises).

**Conclusion and significance**

Six months of Schroth exercises added to standard care produced significant improvements in objective postural measurements in all 3 planes. Interpreting the influential covariates suggest that Schroth curve type, bracing, self-efficacy and personal characteristics also influence outcomes. We recommend these covariates be carefully controlled and reported in future exercise trials.

## O62 Effects of Schroth exercises added to standard care in adolescents with idiopathic scoliosis (AIS) on marker-less surface topography asymmetry measurements – a randomized controlled trial (RCT)

### Eric C. Parent^1^, Maliheh Ghaneei^1^, Samer Adeeb^1^, Sanja Schreiber^1^, Marc Moreau^1,2^, Douglas Hedden^1,2^, Douglas Hill^1,2^, Sarah Southon^1,2^

#### ^1^University of Alberta, Edmonton, Canada; ^2^Alberta Health Services, Edmonton, Canada

##### **Correspondence:** Eric C. Parent

**Introduction**

In studies of suboptimal quality, Schroth exercises have shown promise for slowing curve progression and improving posture in AIS. Few studies have reported quantitative measurements of the effect of scoliosis-specific exercises on the external deformity. Reviews call for randomized and prospective controlled studies on exercises for scoliosis.

This assessor and statistician-blinded RCT (NCT01610908) in AIS aimed to determine the effect of Schroth exercises added to standard of care (EXP group) to standard of care alone (CTRL group) on asymmetry measurements from marker-less surface topography (ST) analysis.

**Methods**

Fifty consecutive participants with AIS, aged 10-18 years, with curves of 10°-45° and Risser 0-5, were recruited from a scoliosis clinic and randomized to the EXP or CTRL group. A Schroth home program adapted to each curve type was taught over five individual sessions, and adjusted weekly according to an algorithm during a therapy visit. Compliance with the home program and the quality of the performance of exercises was assessed weekly using a checklist. Full-torso ST scans were acquired at baseline, 3 and 6 months using a frame standardizing the positioning of the extremities. Scans were analyzed blind to group allocation to calculate the best plane of symmetry by minimizing the distances between the torso and its reflection about the plane of symmetry. Distances between the torso and its reflection were displayed as deviation color maps. Outcomes extracted from the asymmetry patch corresponding to the largest curve were the change in the maximum deviation (MaxDev) and root mean square (RMS) of the deviations within the patch. Linear mixed models were used to test differences between groups over time while adjusting for self-efficacy and curve type covariates.

**Results and discussion**

At baseline, despite lighter weight in the EXP group, groups were similar for age [EXP 13.5 yrs (12.7-14.2) CTRL 13.3 yrs (12.7-13.9)], gender, height, use of a brace (17/group), curve type (≤1 subject difference per type) and Cobb angle [EXP: 29^O^ degrees (95%CI 25-32) CTRL 27.9^o^ (24-32)]. Six patients dropped out (EXP = 4, CTRL =2). For compliance, 76% of visits and 73% of the home exercises were completed. Schroth exercises improved maxDev for the asymmetry patch compared to the deterioration in CTRL significant only at 3 months (*p* = 0.038). Maxdev worsened @3 m in the CTRL group (adjmean ± SE: 15.1 ± 1.4 mm, to 16.6 ± 1.4@3 m, to 13.7 ± 1.4@6 m), but improved with exercises (19.1 ± 1.4 mm, 17.7 ± 1.4@3 m, 18.2 ± 1.4@6 m). The 4C curves presented less severe maxDev than 4CP (*p* = 0.002). Schroth exercises also improved the RMS over the asymmetry patch compared to the deterioration in CTRL significant only at 3 months (*p* = 0.031). RMS asymmetry worsened @3 m during follow-up in the CTRL (AdjMean ± SE: 7.5 ± 0.6 mm, to 8.3 ± 0.6@3 m, to 7.5 ± 0.6@6 m), but was improved with exercises (9.8 ± 0.6 mm, 9.1 ± 0.6@3 m, 9.5 ± 0.6@6 m). The 4C curves presented less severe RMS than 4CP with both less severely affected than 3cp(*p* = 0.002).

**Conclusion and significance**

Six months of Schroth exercises added to standard care produced significant short-term improvements in objective 3D marker-less postural measurements.

## O63 ApiFix treatment for adolescent idiopathic scoliosis (AIS): the importance of Schroth method exercises after the minimal invasive operation

### Nikos Karavidas

#### Scoliosis Spine Laser Centre, Athens, Greece

**Introduction**

ApiFix is an innovative system to treat AIS, which combines a short peri-apical fixation with Scoliosis Specific Exercises. A ratchet-type implant is attached to the apex and gradually elongates through exercises.

**Objectives**

The purpose of the study is to present the short-term results of the ApiFix treatment and to evaluate the effectiveness of Schroth method after surgery.

**Methods**

Prospective case-series study (Level of Evidence IV). 6 females (mean age 15.6 years, Risser 3.7, Cobb angle 41.8^ο^, 2 Lenke Type I and 4 Lenke Type V curvatures) were treated with ApiFix system in Greece. All patients followed a Scoliosis Specific Exercises program for 6 months after operation, under the supervision of a Schroth Certified Physiotherapist. The outcome parameters analyzed were Cobb angle, Angle of Trunk Rotation (ATR), Aesthetics (measured by TAPS questionnaire and TRACE scale) and Pain (measured by Visual Analogue Scale). The average follow-up for the patients was 17.5 months. Unpaired student t-test was used for statistical analysis.

**Results and discussion**

A significant Cobb angle correction of 35.9% (from 41.8^ο^ to 26.8^ο^, *p* = 0.031) was achieved for the whole group. Some of the patients did not have absolute indications for ApiFix treatment and this might have restricted the final correction. One patient had a complication and underwent a revision surgery, due to a backup of the ratchet mechanism that was corrected by locking the mechanism. Another patient had no chance for elongation of the implant and further correction, due to improper length of the mechanism. A further analysis of the pre/post exercises result in the other 4 patients showed that Schroth method reduced the Cobb angle by 3.3^ο^ (from 26.3^ο^ to 23^ο^, *p* = 0.603), the ATR by 2.3^ο^ (from 10.5^ο^ to 8.2^ο^, *p* = 0.252), the TAPS score by 0.7 (from 3.2 to 3.9, *p* = 0.113), the TRACE score by 2 (from 3.75 to 1.75, *p* = 0.001) and the VAS score by 1.3 (from 2 to 0.7, *p* = 0.04). Moreover, the Schroth exercises stabilized the secondary curvatures, normalized the sagittal plane in some cases and educated the patients to unload their spine and avoid mechanical forces by a specific training of Activities of Daily Living (ADL).

**Conclusions and significance**

ApiFix system, with the assistance of Schroth method, can significantly decrease Cobb angle (35.9%, *p* = 0.031) and treat AIS without spinal fusion. Schroth method significantly improved ATR, aesthetics and pain after operation, and a satisfied, but not significant (3.3^ο^, *p* = 0.603) correction was achieved for Cobb angle. Schroth exercises, designed only by a Certified Physiotherapist, must be implemented after ApiFix operation in order to enhance the final treatment result. The proper choice of the most suitable patients with clear indications is of paramount importance, while better quality studies, larger samples and long-term results are needed in future research.

## O64 Exercise versus surgical intervention for pain and disability in adults with lumbar spondylolisthesis

### Despoina Dritsa, Josette Bettany-Saltikov, Nigel Hanchard

#### Teesside University, Middlesbrough, United Kingdom

##### **Correspondence:** Despoina Dritsa

**Introduction**

Spondylolisthesis (SPD) is a condition that directly affects the vertebra and is most commonly seen in the lumbar spine (Earl, 2002). It is a movement or translation of one vertebral body over the other. Slippage may be forward, backward, or sideways, but usually an anterolisthesis (forward movement) is implied by this term (Haun and Kettner, 2005). The most common types of SPD found in the adult population are isthmic and degenerative (Earl, 2002). Even though there are important differences in these two types of SPD, the treatment approach to this condition remains the same. The main management approaches for lumbar spondylolisthesis are conservative treatment, and surgery (Vibert et al., 2006). Both interventions have been proven to work independently but the question still remains as to which route is the most beneficial for a satisfactory clinical outcome both in the short and long-term.

**Objectives**

To determine whether exercise or surgery is more effective for the treatment of lumbar spondylolisthesis in adults.

**Methods**

A systematic search was conducted in MEDLINE, CINAHL, AMED, EMBASE, SPORTDISCUS, and EBMR for articles published through November 2015. PICO was used to design the selection criteria for relevant studies. Quality assessment was evaluated using the 'Physiotherapy Evidence Database' (PEDro). A narrative synthesis was conducted in order to analyse the relationships within and between the studies. Mean numerical values were presented as a mean difference (MD) and the findings were compared to determine potential heterogeneity of treatment effect.

**Results and discussion**

Three RCT's fulfilled all the inclusion criteria. Only one study showed substantially better results in pain and disability for surgery over exercise in the medium and long term (Moller and Hedlund, 2000). However, the evidence relating to efficacy is of low-moderate quality. Two studies reported that the benefits of surgery are likely to outweigh the possible harms (Moller and Hedlund, 2000; Weinstein et al., 2007). Sample size, differences in inclusion criteria, age groups, cultural backgrounds, types of exercise, surgery approaches, and outcome measures makes the effects of the studies included in this review difficult to assess for generalisability and applicability.

**Conclusion and significance**

Following a rigorous search strategy, 3 studies were identified and included in the final review. Internal and external validity was assessed and found to be low to moderate. The results found no significant differences between the two treatment strategies. Only Moller and Hedlund (2000) found posterolateral fusion to be more effective than exercise in the long term. Yet, methodological quality of this paper was low-moderate, thus the results should be used with caution. No clear conclusions were drawn for the best treatment strategy. Further research is very likely to influence the estimated effect of the studies.

## O65 A retrospective study on effect of treatment for adult scoliosis with chronic lower back pain using K-HYU method

### Donghyun Kim^1^, Junlae Kim^1^

#### Seoul Hyun Rehabilitation Hospital, Seoul, Korea

##### **Correspondence:** Donghyun Kim

**Introduction**

One of common complications found in Adult Scoliosis(AS) is chronic lower back pain(CLBP). AS patients with scoliosis are often treated with general physical therapy, simple exercise, injection, and surgical procedure to simply ease the pain.

However, additional treatment is needed considering the fact that compared to normal people patients with scoliosis require different strategy to maintain proper alignment of the spine, pelvis, and the lower extremities, and to have the right posture control. K-HYU method is a 3D approach on spine and peripheral musculoskeletal structure correction, a functional approach on achieving gradual antigravity state and correction in daily life, and a cognitive behavioral training for self-correction, which would be considered as an effective treatment for AS with CLBP.

**Objectives**

To compare the effect of conventional treatment (CT) - focused on pain relief, with treatment using K-HYU Method- a structural and functional scoliosis correction treatment, in treating AS with CLBP, by measuring and comparing the change of 3D curvature of spine and pelvis and health related Quality of Life(QOL) before and after each treatment.

**Methods**

One hundred ten female scoliosis patient in their 20s to 40s who suffered CLBP over 3 months were treated using either CT (Physiotherapy, injection, conventional exercise for lower back pain) or K-HYU Methods in outpatient care for 1 hour a day, 2-3 times a week, and average of total 20 sessions. Korean version of the scoliosis Research society-22 questionnaire was used to measure health related QOL, and Cobb's angle, vertebral rotation, plumb line offset (frontal and sagittal plane), Lumbar obliquities, pelvic parameters (pelvic incidence, sacral slope, pelvic tilt), Thoracic kyphosis, Lumbar lordosis were measured using diagnostic x-ray imaging technique. Distribution of foot pressure and deviation of COG was measured using foot pressure scanner. Data analysis was done using independent T-test and paired T-test. A value of *P* < 0.05 was used to indicate statistical significance.

**Results and discussion**

Compared to CT, K-HYU methods showed greater improvements of SRS-22 scores (function (*p* = 0.003), self-image (*p* = 0.025), mental health (*p* = 0.034), treatment satisfaction (*p* = 0.087), and total score (p = 0.039), Cobb's angle(p = 0.000), vertebral rotation (*p* = 0.000), plumb line offset (sagittal (*p* = 0.027), frontal (*p* = 0.033), lumbar obliquity (*p* = 0.000), lumbar lordosis (*p* = 0.015), Distribution of foot pressure (*p* = 0.044), and deviation of COG (*p* = 0.031).

**Conclusion and significance**

This study shows that treating AS patients with CLBP using K-Hyu methods is an effective way not only to improve alignment of the spine and peripheral musculoskeletal structure, but also easing the pain and improving the QOL. Therefore, in treating CLBP of AS patients, the comprehensive treatment to achieve structural functional correction of deformed spine is a more effective way of treatment than a treatment simply focusing on alleviating the pain.

## O66 Description of physiotherapeutic scoliosis-specific exercise treatments for adults with scoliosis and self-perceived results and compliance with home exercises after discharge

### Amy Sbihli^1^, Eric Parent^2^

#### ^1^Spine Academy Physical Therapy, PLLC, Lexington, MA, USA; ^2^University of Alberta, Edmonton, Canada

##### **Correspondence:** Amy Sbihli

**Introduction**

Physiotherapeutic Scoliosis-Specific Exercise (PSSE) originally focused on reducing the risk of curve progression in growing children. However, adults diagnosed with scoliosis in early or late in life are seeking care from PSSE providers. There is limited research on the use of PSSE and their perceived results by adults with scoliosis.

**Objectives**

The purpose of this study was to assess how adults perceive their use of PSSE in the clinic and training at home impacts their pain, function and quality of life after discharge from outpatient physical therapy.

**Methods**

A 10 question internet survey was sent to all Risser 5 patients (*n* = 52) having been seen at one clinic by a physical therapist trained in PSSE. Three reminders emails were sent by a clinic aide over 13 weeks. Questions developed by the author assessed current age, age at diagnosis, number of visits for treatment and home program development, compliance with home exercise and perceptions of the treatment results. Treatment was provided in one hour sessions including education on curve anatomy, joint protection strategies and modified 3D curve-specific spinal stabilization exercise (Schroth Method). Compliance expectations were 10-20 minutes daily during active treatment and a minimum of 20 minutes 3 times per week after discharge.

**Results and discussion**

Among 52 invitations, 29 responded (56%). Most were female (90%) and diagnosed with scoliosis before 18 years old (64%). Current ages ranged from 18 to >75 years with 40% between 55 and 65 years old. Visit frequency revealed 52% were seen for 5-10 visits and 24% for 11-16 visits. After 5 visits, 38% reported feeling confident about understanding the exercises, while 27% thought it took 5-10 visits. The reported frequency of performing the home exercises was 3-4 times per week for 27%, to 5 times per week for 27% and the remaining 44% reported exercising 1-2 times per week. The home program performed varied from 11-20 minutes for 39% to 5-10 minutes for 44%. Forty percent felt compliant, while 30% reported "finding time" was the largest barrier to compliance. No one reported a negative impact on perceived quality of life. One patient reported no change. "Significant improvement", "moderately positive" and "minimally positive" impact on quality of life were reported by 31%, 51% and 11%, respectively. The rank order of the domains from best to worst perceived treatment results was: posture control (4.59), less pain (4.05), breathing (3.76), function (walk, sit, stand) (3.41), mental well-being (3.41), physical appearance (2.52).

**Conclusion and significance**

Adult with scoliosis seeking conservative care are using exercise and training. PSSE treatment completed in small doses seems to have some positive effects in adults with scoliosis especially on perceived posture control, pain, and breathing. Compliance strategies and follow-up visits may benefit this population's on-going success. Research is needed to document the observed effects of PSSE and ideal dosages in adults.

## O67 Rib vertebral angle difference as a predictive measure for progression of adolescent idiopathic scoliosis in the skeletally immature patient

### Lauren Levey, Mark Holowka, Leigh Davis

#### Children's Healthcare of Atlanta, Atlanta, GA, USA

##### **Correspondence:** Lauren Levey

**Introduction**

While Cobb angle and Risser sign have been shown to be strong predictors of curve progression in patients with adolescent idiopathic scoliosis (AIS), other radiologic factors remain underexplored in this population. In 1972, Mehta developed the radiographic measurement of the Rib Vertebral Angle Difference (RVAD) to diagnose infantile scoliotic curvatures as progressive or resolving. RVAD has been shown to be robust for classifying infantile scoliosis curves, and similar results have been found for juvenile scoliosis, however RVAD has been minimally examined in the AIS population.

**Objectives**

The purpose of this study is to examine the relationship between RVAD and the risk of curve progression in skeletally immature patients with AIS. We hypothesize that RVAD will be an effective measure to predict likelihood of curve progression in this AIS population.

**Methods**

This study retrospectively examined subject data and radiographs from the large, multicenter controlled Bracing in Adolescent Idiopathic Scoliosis Trial (BrAIST, Weinstein et al, 2013 NCT00448448). Digital radiographs were examined from patients with an SRS classification of single thoracic, double thoracic, or double major curve. Using the protocol developed by Mehta, RVAD was measured at the apical (V3), 2 superior (V2, V1), and 2 inferior (V4, V5) vertebra. RVAD was compared to other clinically relevant measures (rotational prominence, Risser sign, etc.). For analysis, subjects were classified as treatment success or failure using the BrAIST threshold of final Cobb angle less than 50 degrees as treatment success. Subjects were also analyzed in classifications of progressive or non-progressive curves, using a threshold of 10 degrees of curvature change to be considered progressive. Multivariable logistic regression was used to create predictive models for curve progression.

**Results and discussion**

One hundred sixty-three subjects met inclusion criteria for analysis; 64 subjects were unbraced and 99 received bracing treatment. No statistical differences in RVAD at the apical vertebra were found between success/failure groups (11 ± 8.3 versus 10.5 ± 9.2) or progressive/non-progressive groups (10.1 ± 7.9 versus 11.9 ± 9.6), regardless of receiving orthotic treatment. Higher RVAD at the V5 vertebra demonstrated a higher, but statistically insignificant, correlation to treatment failure (9.9 ± 7.2 versus 12.3 ± 8.9, *p* = 0.067). Rotational prominence was statistically different between success/failure groups (*p* = 0.04), and similarly, a higher apical RVAD correlated to a higher rotational prominence (*p* = 0.051). Inclusion of RVAD and rotational prominence into the statistical model for curve progression did not change the strength of the multivariate model (ROC = 0.753 versus ROC = 0.756).

**Conclusion and significance**

In this AIS population, RVAD was not a strong statistical predictor of curve progression. Future studies should examine the interaction between RVAD and orthotic treatment, in-brace curve correction, and in-brace RVAD correction with respect to treatment outcomes in AIS.

## O68 Sanders vs. Risser: which system should be used to develop bracing indications?

### Lori A. Dolan, Stuart L Weinstein, BrAIST Study Group

#### University of Iowa, Iowa City, IA, USA

##### **Correspondence:** Lori A. Dolan

**Introduction**

The Cobb angle and skeletal maturity are highly linked to the risk of curve progression. Current bracing indications are based on the Cobb angle and the Risser grade, but evidence from BrAIST implies these indications are too broad, resulting in significant overtreatment.

**Objectives**

The goals of this project were to 1) develop evidence-based bracing indications which minimize under-treatment (not treating high risk patients) without excessive over-treatment (treating low risk patients) and 2) to determine which maturity measure, the Sanders maturity stage (SMS, from a hand x-ray) or the Risser grade (from the full spine x-ray) confers an advantage toward meeting this goal.

**Methods**

We included untreated subjects from the BrAIST database who met current bracing indications and reached a study endpoint. Logistic regression models were created to predict the endpoint of progression to 50¡ prior to skeletal maturity (failure). Two models were developed, one using the Risser grade and the other using the SMS. Other candidate variables included age, sex, maximum Cobb angle, kyphosis, and the SRS curve classification. The minimum probability of failure at which patients should be considered high risk, implying need for a brace, was selected with the goal of keeping the false negative fraction (i.e. under-treatment) at <10% while maximizing overall accuracy (i.e. appropriate treatment).

**Results and discussion**

Data from 86 subjects were included. The Risser model included Cobb angle and sex (R2 = 0.58). In the SMS model, the only other significant predictor was the Cobb angle (R2 = 0.56). To maintain a false negative fraction of <10%, the optimal probability of failure cutoff from the Risser model was ^3^0.25, and ^3^0.30 from the SMS model. Therefore, the models suggest the following indications for bracing: Using Risser: Both sexes at Risser 0, with Cobb angles of 23 or greater. If the Risser grade is 1 or 2, only males with Cobb angles greater than 30 degrees. Using SMS: Both sexes at SMS 1-2, with Cobb angles of 20 or greater, and those at SMS 3, with Cobb angles of 25 or greater. The Risser rule would result in bracing 62% of the sample, compared 59% using the SMS rule. The SMS rule resulted in a 9% false negative fraction with an 80% overall accurate fraction compared to 10% and 76% respectively for the Risser rule. When comparing the maturity indicators, use of the SMS resulted in both slightly lower false negative rates and higher overall accuracy in predicting which patients will have significant curve progression, without the need for separate indications for male and female patients.

**Conclusion and significance**

Applying either of these rules in practice could result in the reduction of the number of patients currently indicated for treatment by approximately 40%. Clinicians need to consider whether these results warrant adding a hand film to the PA and lateral films typically ordered at the initial visit.

## O69 Evaluation of angle trunk rotation (ATR) measurements to improve quality and safety in the office management of adolescent idiopathic scoliosis

### Jill E. Larson^1^, Maximilian A. Meyer^1^, Barrett Boody^1^, John F. Sarwark^2^

#### ^1^Northwestern University, Chicago, IL, USA; ^2^Ann & Robert H. Lurie Children's Hospital of Chicago, Chicago, IL, USA

##### **Correspondence:** John F. Sarwark

**Introduction**

The evaluation, management and follow-up of adolescent idiopathic scoliosis (AIS) occur frequently within clinical practice. Curve status can be assessed with Scoliometer measurements of angle trunk rotation (ATR), which are reliable and reproducible to within 3 degrees and correlate generally with radiographic Cobb angles. This study assessed the longitudinal efficacy, safety and cost savings of integrating ATR measurements to monitor curve status and progression in AIS, and suggests a quality-based management strategy.

**Objectives**

To improve the safety and quality of scoliosis follow-up evaluations.

**Methods**

A retrospective review of medical records between 2004 and 2014 included patients with AIS between 10-17 years and excluded those with Cobb angle > 52 degrees at presentation. Data included sex, menarchal status, ATR measurements, radiographic Cobb angle and Risser stage. Two cohorts were analyzed: Group PRE (pre-menarchal females and males with Risser <5) and POST (post-menarchal females and males with Risser 5). "Unstable" was defined as patients with ≥4 degrees of change from initial to final ATR measurement. The cost of a single PA thoracolumbar radiograph ($36.27) was defined by the 2015 CMS fee schedule. Safety was defined based on the effective radiation dose avoided (0.14 millisieverts/radiograph).

**Results and discussion**

A total of 60 children were included with 46 (76.7%) presenting pre-menarchal (*n* = 42) or males with Risser <5 (*n* = 4) and 14 presenting post-menarchal. There were no unstable curve patterns in the POST group. The use of ATR measurements provided a cost benefit in both the PRE Stable and Unstable cohorts, by avoiding radiographs with an average savings of $161.76 and $137.83 respectively. Similarly, within POST, there was an average cost savings of $105.18 per patient. The safety benefit of using ATR measurements included avoiding an average of 0.62, 0.53 and 0.4 millisieverts of radiation in the PRE Stable, PRE Unstable and POST groups respectively.

**Conclusion and significance**

An evaluation strategy that includes ATR measurements provides for a reliable, cost-effective and safety advantage in the monitoring of curve progression in both skeletally mature and immature patients with AIS. The study is limited by its retrospective methods; however, the findings suggest that stable ATR measurements (without radiographs) are a safe and cost effective alternative to serial radiographs in the clinical monitoring of AIS.

Recent evidence from 25 years of scoliosis treatment in Denmark noted a cancer rate 17 times that of an age-matched population. Thus, reducing radiation exposure during scoliosis monitoring by using ATR measurements has important clinical significance for cancer risk reduction.

## O70 Minimal important differences in Scoliosis Research Society-22r, spinal appearance questionnaire, Cobb angle, and Biering-Sorensen back muscle endurance test following a six-month Schroth exercises intervention in adolescents with idiopathic scoliosis

### Sanja Schreiber^1^, Eric C. Parent^1^, Douglas M. Hedden^1,2^, Douglas L. Hill^1,2^

#### ^1^University of Alberta, Edmonton, Canada; ^2^Alberta Health Services, Edmonton, Canada

##### **Correspondence:** Sanja Schreiber

**Introduction**

Reporting of whether participants reached the minimal important difference (MID) should complement statistical tests in clinical studies. The MID is unknown for most commonly used outcomes in adolescents with idiopathic scoliosis (AIS) treated conservatively.

**Objectives**

To establish the MID of the Scoliosis Research Society 22r (SRS-22r), Spinal Appearance Questionnaire (SAQ), Cobb angle and Biering-Sorensen back muscle endurance test (BST) in AIS following a 6-month Schroth intervention added to standard of care (observation or bracing).

**Methods**

This was a secondary analysis of a randomized controlled trial (RCT) (NCT01610908). Fifty patients with AIS, aged 10-18, with curves 10¡-45¡, with or without brace, and all maturity levels were included. The intervention consisted of a 30-minute daily home program of Schroth exercises and weekly 1 h-long supervised sessions. Exercises were adjusted weekly during supervised sessions according to an algorithm. MID were determined using anchor- and distribution-based methods. Anchor-based MID was defined as the mean pretreatment/follow-up difference in the outcomes in the group of patients who reported at least +2 (a little bit of improvement) on a 15-point Global Rating of Change (GRC). Distribution-based MID included standard error of measurement (SEM) and minimal detectable difference (MDD95).

**Results and discussion**

The mean age was 13.4 ± 1.6 years and the mean Cobb angle was 28.5 ± 8.8o. After 6-months in the trial, 20 participants reported at least +2 change on the GRC. All, but one of the questionnaires' anchor-based cut-offs were below their SEM, with MDD95 producing change values higher than seen in this population. Most questionnaires changes scores did not correlate with the anchor, suggesting these outcomes did not reflect perceived changes. Therefore, based on the SEM, the questionnaires' MID were: SRS-22r pain 0.12, self-image 0.20, function 0.11, and total 0.13; SAQ general, curve 0.41, prominence 0.32, trunk-shift 0.24, waist 0.60, shoulders 0.45, and chest 0.79. Anchor-based MIDs for the Cobb angle and BST were larger than their SEMs, but smaller than the MDD95. MID for the largest Cobb angles was 3.4¡ and for the sum of curves was 8.7¡ based on their MDD95. Anchor-based MID for the BST was 36.15 sec representing a more realistic change than the expectation corresponding to MDD95 (62.79 sec).

**Conclusion and significance**

This is the first study to assess the MIDs for the Cobb angles, Biering-Sorensen test and SAQ scores in patients with AIS. The SRS-22r and the SAQ questionnaires have limited responsiveness in patients with AIS treated conservatively. The anchor-based MID for the Cobb angle was smaller than the typically used threshold of 5 degrees. Until better quality of life and perceived appearance tools for assessing change in this group become available, using distribution-based is recommended to monitor therapeutic effects understanding that results might not convey the patients’ perspective.

## P1 CAD/CAM assisted bracing for children with infantile or juvenile scoliosis

### John Thometz^1^, XueCheng Liu^1^, Robert Rizza^2^

#### ^1^Children's Hospital of Wisconsin, Medical College of Wisconsin, Milwaukee, WI, USA; ^2^Milwaukee School of Engineering, Milwaukee, WI, USA

##### **Correspondence:** John Thometz

**Introduction**

Computer-Aided Design (CAD)/ Computer Aided Milling (CAM) method has been accepted in the orthotic industry for adolescent scoliosis. There are few reports of brace treatment in children with infantile (IS) or juvenile scoliosis (JS) using CAD/CAM. This presentation describes our experience. Also there is a paucity of reports to discuss this technique in the manufacture of orthoses for those populations.

**Objectives**

The goals of this study were: 1) to utilize CAD/CAM methodology to create a brace from the spine when placed in the corrected position for children with the early onset of scoliosis (EOS); 2) to investigate changes of Cobb angles in AP view X-ray in or out of CAM customized brace in 3, 6, 12 months.

**Methods**

This study was retrospectively case controlled and was approved by the IRB. Patients with EOS received CAD/CAM bracing after series of casting. We report eight patients (5 boys and 3 girls) who have been followed more than 12 months. The average age is 4 years and 5 month-old (range from 18 months-old to 85 months-old). Spine correction was performed using a stockinette to manipulate the curvature in the coronal and transversal plane, simultaneously longitudinal traction applied. The scanning can be done in the clinic or operating room while the patient was under general anesthesia for younger patients. The trunk was scanned and data was sent for CAD and CAM, where the engineer designed and modified the asymmetric brace using the software. The braces were manufactured within 5 days. All children were radiographically evaluated before the use of brace, in the brace, and out of brace at 3, 6, and 12 months. The compliance for the use of brace was documented. A descriptive data analysis was applied.

**Results and discussion**

Eight children had orthotic treatment for 12 months or more. All parents reported that children continually wear the brace for 12 months. The Cobb angles were reduced from pre- brace (38.1 °) to in brace (26.8°). The curves were at 35.7 ° at 12 months out of brace.

Corrective manipulation with the CAD/CAM method helps control EOS and results in approximately 30% reduction of Cobb angle from out of brace to in brace. Cobb angle remains stable at follow-up. The brace fit helps with patient acceptance.

**Conclusion and significance**

Not only does CAD/CAM based method shorten the process time of orthotics, but also provides a better fit and correction in the treatment of EOS. This asymmetric brace provides an alternative management for children with EOS who were not tolerant to the casting or TLSO.

## P2 Serial EDF casting in infantile idiopathic scoliosis

### Channing Tassone^1,2^, XueCheng Liu^1,2^, Benjamin Gundlach^2^, Sergey Tarima^3^

#### ^1^Deptartment of Orthopaedic Surgery, Children's Hospital of Wisconsin, Milwaukee, WI, USA; ^2^Medical College of Wisconsin, Milwaukee, WI, USA; ^3^Division of Biostatistics, Institute for Health and Society Education, University of Kentucky, Lexignton, KY, USA

##### **Correspondence:** Channing Tassone

**Introduction**

Elongation-derotation-flexion (EDF) casting has been considered as an effective approach to treating early-onset-scoliosis (EOS). EDF casting provides three-dimensional corrective forces to the deformed spine. Given the evidence of the effects of EDF in the literature, it is a potential tool to drastically improve the skeletal deformity in these children with infantile scoliosis. Preliminary studies have shown that the earlier treatment is begun, the better the potential outcomes.

**Objectives**

Aims of this current study were: 1) to evaluate changes of Cobb angles before and after 6 month and 12 months using Mehta modification of Cotrel-Morel casting technique; 2) to assess thoracic height at 6 month and 12 months post-EDF as compared to the baseline.

**Methods**

This was a retrospective study. Nine children with infantile idiopathic scoliosis were treated by EDF serial casting with anterior and posterior windows and followed for at least 12 months. All of them had x-ray measurements at 0, 6 months, and 12 months after the application of EDF. Casts were changed every 2-4 months. Clinical data was collected, including age, Cobb angles in AP view, thoracic height, number of casts, complications, and time at the treatment. Paired t-test was performed to compare these clinical data between 0 and 6 months or between 0 and 12 months.

**Results and discussion**

Age at initial EDF was 17.5 ± 5 months (9-24). Number of EDF was 6.8 ± 2.1 (3-10). Age from diagnosis to treatment was 4.1 ± 4.2 months (1-13). Pre-EDF Cobb angle was 44.4° ±14.2°. There was a significant reduction of Cobb angle at 6 months post-EDF (35.1° ± 15.1°) (*p* = 0.048) and reduction of Cobb angle at 12 months post-EDF (33.8° ± 22.4°) (*p* = 0.13). The thoracic height was significantly increased from 12.8 ± 0.9 cm at initial EDF to 14.6 ± 1.3 cm at 6 months post-EDF (*P* = 0.002) as well as to 15.3 ± 1.6 cm at 12 months post-EDF (*p* = 0.002). EDF serial casting is effective in the treatment of infantile idiopathic scoliosis with significant improvement of Cobb angles coupled with continuous growth of the trunk height.

**Conclusion and significance**

EDF casting can cure infantile scoliosis thus making any surgery unnecessary. Even if no cure is achieved it can be successful in delaying surgical intervention for severe infantile idiopathic scoliosis.

## P3 The effect of the SpineCor® Dynamic Corrective Brace on coronal balance in patients with Risser value greater/equal to 3

### Alison Grant^1^, Raman Kalyan^2^, Waleed Hekal^2^, Cheryl Honeyman^2^, Tim Cook^1^, Scott Murray^2^

#### ^1^The Spine Corporation, Millennium House, Foxwood Road, Chesterfield, S41 9RF, UK; ^2^James Cook University Hospitals, Marton Road, Middlesbrough, TS4 3BW, UK

##### **Correspondence:** Alison Grant

**Introduction**

An evaluation was undertaken to assess the effect of the SpineCor® Dynamic Corrective brace on the Coronal Balance in a group of patients with thoracolumbar curves and with a Risser value ≥3. The SpineCor® Dynamic Corrective brace was used because of the potential to produce postural changes in the patient integrating the whole neuro-muscular system. The degree of skeletal maturity was an important element of the evaluation because it is assumed that with skeletally mature young patients little of no change can be introduced. The effect on the Coronal Balance of this patient group was of particular interest because in adulthood pain is associated in thoracolumbar curves whose Coronal Balance is significant.

**Objective**

To assess the effect on the Coronal Balance using the SpineCor® Dynamic Corrective Brace in a group of patients with thoracolumbar curve and a Risser value ≥ 3.

**Method**

The evaluation included patients who were all diagnosed with thoracolumbar curve and then referred for bracing. Criteria for inclusion were 1) SpineCor classification as a thoracolumbar scoliosis 2) have had no previous treatment 3) at the onset of treatment to have a Risser value ≥ 3. Over a one-year period out of a possible 20 patients, six patients fell into the criteria. Of this group, five patients have completed and one is still under treatment. The patients were braced by an Orthotist for a minimum of 18 months/or until Risser 4 using the SpineCor® Dynamic Corrective Brace. Each patient was reviewed as per the SpineCor protocol and had x-rays taken in brace (WB), every six months. At the end of treatment, an x-ray was taken without the brace (WOB) and the Cobb measurement was compared to the previous in brace x-ray. If the WOB Cobb measurements were within 5° of the last WB x-ray, weaning from the brace commenced.

**Results and discussion**

All the patients were female with average of 15 years 3 months. All the patients were Risser ≥ 3. The Cobb angle pre-treatment ranged from 22° to 30° with a mean of 27°; post treatment the range was from 9° to 34° with a mean of 20°. The mean Coronal Balance at T1 changed from 16.2 mm pre-treatment to 3.6 mm post treatment and at T12 this changed from 25.4 mm pre to 14.2 mm post treatment. Out of the five patients all a reduction in their Coronal Balance measurements.

**Conclusion and significance**

In adulthood, pain has been associated with patients whose Coronal Balance is significant and this can be a particular issue in patients with thoracolumbar curves. The evaluation looked at this patient group due to the potential to progress and develop pain in adulthood. The results, even though a small sample, does show that the SpineCor® Dynamic Corrective brace is effective in improving the coronal balance and could have a positive role to play in the more mature young patients where there is a potential for the Coronal Balance to increase over time.

## P4 Brace associated to specific exercises is able to improve spondylolisthesis in growing patients

### Morena Pitruzzella^1^, Sabrina Donzelli^1^, Fabio Zaina^1^, Stefano Negrini^1,2,3^

#### ^1^ISICO Italian Scientific Spine Institute, Milan Italy; ^2^University of Brescia, Brescia, Italy; ^3^Don Gnocchi Foundation, Milan, Italy

##### **Correspondence:** Sabrina Donzelli

**Introduction**

The actual evidence concerning effective treatment for spondylolisthesis is really sparse and relevant data able to help clinicians for clinical decision making are lacking. This is why a common path among experts in spinal disorders has not been defined yet, and the best approach is still to be discovered. Even though it is a quite rare condition, with an incidence comprised between 4% and 6% in growing subjects, it can be also associated to other deformities of the growing spine like scoliosis and hyperkyphosis, thus affecting the clinical approach. Spondylolisthesis is frequently discovered occasionally; in other cases, its first symptom is back pain. It has been demonstrated a risk of progression during growth, this is why a conservative treatment is recommended by some authors. To better understand how to manage with these kind of diseases, observational studies are required.

**Objectives**

The main aim of the present study was to evaluate the short term effects of conservative treatment (brace and exercises) in a population of growing subjects affected by spondylolisthesis.

**Methods**

Participants: The selected population came from a prospective collection of clinical data, the included patients fulfilled the following inclusion criteria: spondylolisthesis as main diagnosis, at least one year of follow-up, with available clinical data of at least three visits; a minimum of two Lateral X-rays at start and after one year; age below 18 years. Outcomes considered: the percentage of the olysthesis according to the Meyerding Classification, and the SRS-22 mean score for pain domains, were compared between start and after one year of treatment. Statistical analysis: considering the normal distribution of data a paired double-tailed t-test was performed, with alpha set at 0.05.

**Results and discussion**

In the sample considered, 49 patients were treated with braces (23 females, 26 males) and 10 (6 females, 4 males) treated with specific exercises only. The mean age was 12.3 (SD = 2.9). Among brace treated subjects the 49% improved after one year of therapy, 47% were stable and only the 4% worsened more than 5%. The mean percentage of the olysthesis at short term follow-up was 14.3 and resulted significantly improved from start (Mean at start 19.3 SE 0.99 SD 6.92, CI 95% 17.3-21.3; Mean percentage at short term 14.6 SE 0.88 SD 6.19 CI 95% 12.8-16-3; *p* < 0.0000). Among not brace patients the differences between start and after one year of treatment did not resulted statistically significant (*p* = 0.41). For what concern the pain domain average scores at the SRS -22 any statistically significant difference was found (*p* = 0.62).

**Conclusion and significance**

This study shows that the combination of activity restriction, specific exercise and bracing, have a high rate of improvement or stabilization of the spondylolisthesis at one years of follow-up, in growing subjects. Additional studies are needed to support these results and to clarify the controversy regarding the most effective therapy for these patients.

## P5 Cases of bracing infantile scoliosis with the new Lyon brace: ARTbrace

### Jean-Claude de Mauroy^1^, Frédéric Barral^2^, Sophie Pourret^2^

#### ^1^Clinique du Parc, Lyon, France; ^2^Lecante Group, Lyon, France

##### **Correspondence:** Jean-Claude de Mauroy

**Introduction**

The Min Mehta's serial casting is usually used for infantile scoliosis. The ARTbrace was created to replace the plaster cast in the Lyon management for AIS. It was therefore tempting to use it to replace the serial casting for infantile scoliosis. In two cases, traditional methods found themselves in failure and we were asked to realize an infantile ARTbrace.

**Objectives**

Infantile scoliosis is rare and studying each case is most useful and can help other teams.

**Methods**

The first problem has been the miniaturization of the brace with use of a 3 mm polycarbonate. The posterior metal bar was reduced in proportion so as the anterior closures. The second problem is the impossibility of achieving the regional moulding for children under 3 years which cannot maintain asymmetric postures. The problem is solved by using the mirror technique. The first moulding is realized in passive axial elongation, meaning reducing the curvature by pulling the child by the upper limbs. During the superimposing step, we invert the image, creating a reverse torsion of the initial scoliosis. Thus a major expansion in the concavity is performed for asymmetrical correction of scoliosis and breathing.

**Results and discussion**

The case of Olympe: Early Onset Scoliosis was discovered at the age of 2 years with a right thoracolumbar curve. At the age of 3 years the curve is 30°. Olympe was not compliant with the first Milwaukee that was quickly replaced by an asymmetrical polyethylene TLSO well-worn during 3 years. Despite bracing, the scoliosis is still progressing to 42° without brace and in-brace correction is 25°. At 8 years, it was possible to achieve the classic regional moulding in 3 steps. The in-brace correction is complete at 0°. At six month follow-up, the angulation without brace is 17°, a little better than under the TLSO. Olympe prefers the polycarbonate. The case of Adele: Adele 2 1/2 years, lives in a North West town of France. At the age of three months, her left infantile scoliosis is discovered. Her Scoliosis is highly progressive because the curve T6-T12 reaches 95° on May 31, 2015. A serial casting from May to July was realized, and then a TLSO brace adapted. MD and CPO are doing their best, but the correction is limited to 60°. The CAD/CAM Mirror technique was used. The in-brace angulation is reduced at 36° (62%), the child is well balanced in the frontal plane and the kyphosis is corrected in the sagittal plane. Discussion: For the infantile scoliosis till now, there were not many alternatives to sequential and repetitive surgery. The 3 mm polycarbonate seems to be able to replace the Min Mehta's serial casting and the polyethylene TLSO.

**Conclusion and significance**

By comparing the results obtained in the same infantile scoliosis by a conventional TLSO and the ART, it is possible to better appreciate the technology differences. In both cases, the results seem to be better for the 3 mm polycarbonate.

Informed consent has been obtained from the patient for publication.

## P6 Cases of bracing adult scoliosis with the new Lyon brace: ARTbrace

### Jean-Claude de Mauroy^1^, Frédéric Barral^2^, Sophie Pourret^2^

#### ^1^Clinique du Parc, Lyon, France; ^2^Lecante Group, Lyon, France

##### **Correspondence:** Jean-Claude de Mauroy

**Introduction**

Scoliosis is a major demographic health issue in the adult population with pain, imbalance and angular curve progression. In a series of 158 adult scoliosis treated by classical polyethylene brace and reviewed on average 8 years after the start of treatment, it appears that current braces fail to stop the kyphotic evolution of adult scoliosis and justifies the improvement of existing braces.

**Objectives**

As the high rigidity of ART 4 mm polycarbonate is better tolerated by children than the conventional polyethylene; it was logical to use the new concepts of "baby lift" and overall untwisting for adults. The first encouraging results justify this presentation especially as there is to date no other solution.

**Methods**

As with adolescent scoliosis, we use the regional moulding. The third mould is made in maximal inspiration, because the expansion in the concavity is lower than for children. The visual control of the instantaneous 3D scan allows a perfect alignment in the frontal and sagittal plane. The superposition of the three mouldings is carried out by the specific OrtenShape software. The usual wearing protocol of the brace is of 4 hours a day. As adult patients are evolving in kyphosis, the posterior axillary support is lower than for children. Similarly, the anterior abdominal expansion due to the restoration of lordosis improves tolerance.

**Results and discussion**

An ARTbrace was performed in 32 adult patients from February to November, 2015. Although there are difficulties for the camptocormia, all patients appreciate the anterior closing by the ratcheting buckle of the ART. One of our patients, passing from polyethylene to polycarbonate, sums up the situation; "Before, I wore the brace, now the brace wears me".

Our first case is a lady born in April 1931. She has a thoraco-lumbar scoliosis (T11-L3 52°) and especially a high thoracic kyphosis of 88° with significant walking difficulties. The result is: kyphosis in-brace correction at 48° and scoliosis at 38°. Unfortunately, the brace is worn less than 4 hours per day as she has leukaemia. Our second case is an old patient treated by the former polyethylene bivalve brace with sterno clavicular support. She presents a scoliosis T5-T12 80° / T12-L4 60°. Since an accident in 2013, the former brace is no longer suitable. A hip flexion and a pelvic retroversion are compensating partially the high thoracic kyphosis. ART adult is completed in March 2015. From the beginning, twisting sensation disappears, less lumbar pain, she is able to climb three floors without breathlessness. At the 6-month follow up, the brace is worn every morning, is very well tolerated and the high thoracic kyphosis is improving at 45°.

**Conclusion and significance**

The adult ARTbrace has the advantage of correcting scoliosis as well as kyphosis. The lateral thoracic support is better tolerated than the sternoclavicular support of a sagittal 3 points system. The anterior closure is a benefit to patients. The absence of abdominal compression limits the vesical and digestive complications.

## P7 RIGO system Cheneau brace is the new standard in bracing: In-brace correction is 45% better than TLSO

### Kelly Grimes, Nicholas Feinberg, Jennifer Hope, Hagit Berdishevsky, Prachi Bakarania, Hiroko Matsumoto, Hasani Swindell, Julie Yoshimachi, David Roye, Michael Vitale

#### Columbia University Medical Center/Morgan Stanley Children's Hospital, New York, NY, USA

##### **Correspondence:** Michael Vitale

**Introduction**

Bracing is the mainstay of the conservative treatment of AIS. In-brace correction is cited in the literature as a key prognostic indicator of the success of bracing treatment.

**Objective**

The purpose of our study was to compare immediate in-brace major coronal curve correction at six weeks between patients who received a Rigo System Cheneau (RSC) brace and a thoracolumbar sacral orthosis brace (TLSO) brace.

**Methods**

This is a retrospective cohort study of patients with idiopathic scoliosis who underwent brace treatment between 2013-2015 with a major Cobb angle of 25^0^ and greater, and Risser score of 0 or a Sanders score of 4 or less. The choice of the RSC brace or the TLSO brace prescription was based on a host of factors including patient preference, provider preference, and insurance coverage. The percent in-brace correction of major coronal curve at 6 weeks was calculated as an outcome.

**Results and discussion**

Twenty seven patients who received RSC bracing and 25 patients who received TLSO were identified. Baseline degree of coronal curvature, age, Risser scores or Sanders scores, and gender were similar. The RSC brace achieved significantly greater in-brace correction (49%) compared to the TLSO brace (27%) with mean difference of 22% (95% CI: 6%-38%, *p* = 0.010).

**Conclusion and significance**

Compared to the traditional TLSO brace, the RSC brace is associated with increased in-brace spinal curvature correction at 6 weeks. The initial in-brace correction of a patient’s brace is vital to the efficacy and success of this non-surgical treatment. Compared to a TLSO brace, the RSC brace is associated with dramatically increased in-brace spinal curvature correction at 6 weeks. At our institution, we continue to monitor these patients for curve-progression every 6 months until skeletal maturity or progression to surgery.

## P8 Critical appraisal for existing clinical guidelines on the detection and conservative care for adolescent idiopathic scoliosis (AIS) using AGREE II

### Julie Touchette^1^, Anissa St-Jean^1^, Danica Brousseau^1^, Louise Marcotte^2^, Jean Théroux^3,4,5^, Chantal Doucet^1^

#### ^1^Université du Québec à Trois-Rivières, Trois-Rivières, Québec, Canada; ^2^Private Practice, Montréal, Canada; ^3^Faculty of Medecine, University of Montreal, Montreal, Québec, Canada; ^4^Research Center, Sainte-Justine University Hospital Center, Montreal, Quebec, Canada; ^5^Murdoch University, School of Health Profession, Murdoch, WA, Australia

##### **Correspondence:** Chantal Doucet

**Introduction**

A plethora of evidence-based (EB) guidelines for the surgical treatment of AIS exist. However, the scientific evidence regarding detection and the clinical management pertaining to conservative care for AIS are sparse. Adolescents seeking care for musculoskeletal conditions including scoliosis, mainly for aesthetic reasons and back pain, are most likely seen by health care professionals (HCP) including chiropractors and account for 10 and 37% of office visits. HCP make clinical decisions based on the most reliable data available. Consequently, recommendations made through clinical practice guidelines (CPG) are designed to improve health care delivery and outcomes for the patient.

**Objectives**

The aim of this study is twofold: to appraise the methodological quality and to determine the applicability of the existing clinical practice guidelines (CPG) on conservative care for AIS within the chiropractic profession.

**Methods**

CPG were collected using electronic databases covering 2005-2015: Academy Complete Search, Medline, Cinahl and Grey literature in French and English languages. Only the CPG concerned with the detection and conservative care for AIS were included. Among the three guidelines that were initially collected, only one (SOSORT 2011) met the criteria, as the others were built as practitioner’s guides. A panel of five experts, selected through a purposive sampling method, used the AGREE II tool, which is divided into 6 domains (D1 scope and purpose, D2 stakeholder involvement, D3 rigour, D4 clarity, D5 applicability and D6 editorial independence), to assess the guideline methodological quality.

**Results and discussion**

The experts completed the AGREE II tool. The percentage score of the different CPG evaluations for all domains ranged from 43 to 88%. Three domains (D1, D4 and D6) scored above 85%, being of highest quality. Two domains (D2 and D3) scored an average of 75% and domain (D5) being the lowest quality according to the expert panel. Based on its overall quality, the SOSORT guideline can be recommended for practice, but would require some adaptation.

**Conclusion and significance**

This overview stand on AGREE II scoring showed the SOSORT guideline 2011 recommendations are helpful for the detection and diagnosis of AIS. Furthermore, they can be useful for HCP in the conservative treatment of AIS, although more studies are required to confirm the outcomes and applicability for the chiropractic practice.

## P9 Contemporary clinical application of computer-aided design and computer-aided manufacture (CAD/CAM) technology to orthotic management of adolescent idiopathic scoliosis

### Yangmin Lin, Man Sang Wong

#### Interdisciplinary Division of Biomedical Engineering, The Hong Kong Polytechnic University, Hong Kong, China

##### **Correspondence:** Yangmin Lin

**Introduction**

Adolescent idiopathic scoliosis (AIS) is a three-dimensional (3D) spinal deformity with lateral curvature and vertebral rotation that may progress if left untreated. Spinal orthotic treatment is generally applied to control the progression of deformity. Compared to the conventional manual method (CMM), using CAD/CAM method (CCM) in design and manufacture of spinal orthosis is believed to offer additional advantages. Nevertheless, the clinical application of CCM has not been widely studied.

**Objectives**

This study aimed to review various studies using CAD/CAM systems in design and manufacture of spinal orthosis, and their relevant evidence on treatment effectiveness for AIS.

**Methods**

This review searched the keywords, "scoliosis", "orthosis", "orthoses", "spinal orthosis", "AIS", "orthotic", along with "CAD/CAM", "computer-aided", "computer", in the databases of AbleData, Academic Search Premier, CINAHL, CIRRIE, RECAL Legacy, Compendex, EMBASE, MEDLINE/PubMed, Global Health and Web of Science, IBSS, Native Health Databases, REHABDATA, and Scopus. All the relevant abstracts were screened, and those studies related to design and fabrication of spinal orthosis for AIS using CMM and CCM were included.

**Results and discussion**

Nine relevant articles were found. In the comparison between CMM and CCM for spinal orthosis, using CCM could offer more benefits such as simpler and time saving, digital documentation, more design flexibility and comparable cost as CMM. The studies suggested that spinal orthoses fabricated by CCM are effective in treating AIS at immediate in-brace correction, short-term curve reduction or progression ^2^5¡ after orthotic management, and some reported similar treatment outcome as CMM. It was found difficult to achieve a large scale of randomized control trial and some drawbacks in study design such as small sample size, different inclusion/exclusion criteria and various types of spinal orthosis. This would be difficult to compare and draw solid conclusion. Future studies should consider the patient's compliance as well as time response to orthotic treatment - at least 2 hours after putting on or taking off spinal orthosis before conducting assessment on the effect of spinal orthosis. In the application of CCM, some limitations should be solved such as the health and safety issue due to the polyurethane dust and long learning curve. Additive manufacture of orthosis is getting more mature that may lead the current CCM for spinal orthosis to a higher level of advancement.

**Conclusion and significance**

CCM could achieve technical and clinical benefits but some drawbacks were noted. To enhance CCM in clinical application for betterment of both the patients and clinicians, more clinical researches to test new fabrication technologies would help to improve the current clinical practices.

## P10 Dynamic strength, flexibility and symmetry of subjects with adolescent idiopathic scoliosis

### John MacMahon^1^, Edward MacMahon^1^, Jeremy Boyette^1^, Luke Stikeleather^2^

#### ^1^Moyarta 2, LLC, The Plains, VA, USA; ^2^National Scoliosis Center, Fairfax, VA, USA

##### **Correspondence:** John MacMahon

**Introduction**

There is information on passive flexibility in subjects, but limited information on the dynamic strength, flexibility and symmetry of adolescents with idiopathic scoliosis (AIS).

**Objectives**

We have quantified the strength, flexibility and symmetry of subjects with AIS to test the hypothesis of asymmetric strength as a present underlying contributor to the development of adolescent idiopathic scoliosis.

**Methods**

Using a new piece of exercise equipment with dynamic visual and force feedback, we quantified subject generated forces and spinal geometry during a series of exercises. During these exercises, subjects actively recruited their shoulder, back and arm muscles, resulting in changes in spinal alignment. Three exercises were performed with a minimum of 5 repetitions for each. The first exercise used both arms for bi-lateral action (Parallel) and the next two exercises worked one side at a time for Leverage and Compression Exercises. Force quantification was achieved with bi-lateral load sensors and geometric quantification used real-time image tracking of 3 markers placed on the subject's back. Forces were normalized to % bodyweight (%BW). Symmetry score is defined as the following: Left Force (%BW)/Right Force (%BW) a score of 1.00 being perfect symmetry. Pair-wise Student's t-tests were used to test the population's symmetry score for significance.

**Results and discussion**

We quantified the normalized strength, flexibility and symmetry of 10 subjects with AIS. In the Parallel Exercise, subjects were able to average 45%BW. Simultaneous and independent measurements of left and right force generation revealed an average symmetry score of 0.98. In the one-sided Leverage and Compression Exercises, subjects generated forces of between 29%BW - 32%BW, with symmetry scores of 0.98 and 1.05 respectively. (*p* > 0.05 for all t-tests). Flexibility was measured by Range of Motion within each repetition and averaged over each exercise. It was 3.0 degrees, for the Parallel Exercises and from 3.0 - 4.6 degrees in the Leverage and Compression Exercises. Using this new piece of exercise equipment, we have achieved quantification of dynamic strength, flexibility and symmetry in subjects with AIS as well as providing them real-time bio-mechanical and visual feedback.

**Conclusion and significance**

We were able to successfully quantify strength, flexibility and symmetry in a series of 10 subjects with AIS. The symmetry score in this population was within 2%-5% of perfect in all three exercises (Parallel, Leverage and Compression) discounting the hypothesis of asymmetric strength as contributing factor in this small population. A dedicated program of these dynamic exercises may be a provocative tool to objectively quantify improvements in any exercise sequence. Incorporation of such a tool may present a synergy of conservative management by bracing with the small but growing objective evidence of dedicated exercises for AIS.

## P11 Preliminary results of Schroth physiotherapy in a female patient with cerebral palsy and neuromuscular scoliosis: case study

### Andrea Lebel^1^, Victoria Ashley Lebel^2^

#### ^1^Ottawa & District Physiotherapy Clinic and Scoliosis Physiotherapy and Posture Centre, Ottawa, Canada; ^2^Saba University School of Medicine, Devens, MA, USA

##### **Correspondence:** Andrea Lebel

**Introduction**

Cerebral palsy (CP) is a neurological disorder that affects muscle tone, movement, and motor skills. Children with CP have varying degrees of physical disability and an increased risk of developing scoliosis. Although physiotherapy is an important part of CP treatment, Schroth physiotherapy (PT) was never initially designed to treat neuromuscular scoliosis in these patients.

**Objectives**

The main objective of this preliminary case study is to demonstrate overall physical improvements in a patient with CP and neuromuscular scoliosis treated with Schroth PT.

**Methods**

A 20-year-old female with neuromuscular scoliosis and GMFCS Level-I CP with hemiparesis was diagnosed with scoliosis at age 11. She was initially treated with general PT exercises and massages and at age 15 began weekly chiropractic treatment. A radiograph ordered by the chiropractor showed a 50° Cobb angle right thoracic scoliosis. After 5 years of chiropractic treatment, the patient decided to try a new method of conservative scoliosis management and began Schroth PT in August 2015 at age 20. The patient received weekly 1-hour individual Schroth PT sessions with the physiotherapist beginning in August 2015. Angle of trunk rotation (ATR), sagittal profile, and vital capacity (VC) measurements were recorded at the initial PT assessment on August 12, 2015 and 3 months later on November 27, 2015. The ATR was measured using a scoliometer. The sagittal profile was measured using a digital inclinometer and measured at 4 different levels: C6-7, T1-2, T12-L1, and S1-2. Vital capacity was measured using a spirometer. Three spirometry measurements were taken and the highest value was reported. Balance and gait was also assessed. Video and digital photographs were taken to follow patient progress over the course of Schroth PT. The SRS-22 questionnaire was also used to monitor functional activity, pain, self-image, and mental health status of the patient.

**Results and discussion**

ATR decreased by 4° from 24° at the thoracic apical region on initial assessment to 20° 3 months after beginning Schroth PT. The sagittal profile for C6-7, T1-2, T12-L1, and S1-2 were 11°, 2°, 2°, and 5°, respectively, on initial assessment. After 3 months of Schroth PT, the sagittal profile for C6-7, T1-2, T12-L1, and S1-2 was 12°, 5°, 2°, and 15°, respectively. Vital capacity increased by 550 mL from 1600 mL on initial assessment to 2150 mL after 3 months of Schroth PT. Improvements were also observed in upper and lower extremity range of motion, gait, and static and dynamic balance. Over the course of Schroth PT, the mean SRS-22 scores remained >4.

**Conclusion and significance**

In this preliminary case study of a 20-year-old female with neuromuscular scoliosis and CP, improvements were objectively observed using ATR, sagittal profile, and VC measurements over the course of 3 months of Schroth PT. Schroth PT and possibly other types of physiotherapy scoliosis-specific exercises (PSSE) can prove beneficial in improving the quality of life and posture in CP patients with neuromuscular scoliosis.

Informed consent has been obtained from the patient for publication.

## P12 The use of a modified Schroth program helped reduce scoliosis curve in a short term follow-up in a young boy with spinal muscle atrophy TYPE III - a case report

### Chintan A. Pancholi-Parekh

#### Pediatric Rehabilitation New York (PRNY), Totowa, NJ, USA

**Introduction**

Spinal Muscular Atrophy (SMA) is a progressive neuromuscular disease. It presents in infancy and childhood. Neuromuscular scoliosis is a common condition that impacts patients of this population. The primary treatments for neuromuscular scoliosis have been occasional bracing (TLSO) and/or surgical intervention. Schroth based scoliosis specific exercise is a conservative approach to idiopathic scoliosis that has been used clinically for decades in Europe. This method has shown some effectiveness in the reduction of Cobb angle in patients with Adolescent Idiopathic Scoliosis (AIS). Based on one case clinical result, it is proposed that the utilization of SSE with some modification, can be beneficial in the halting or short term regression of the Cobb angle in neuromuscular scoliosis.

**Objectives**

The purpose of this paper is to report the treatment plan and the observations of the effects of modified Schroth based Scoliosis Specific exercises on one patient with SMA and scoliosis.

**Methods**

Subject: An 8-year old boy with SMA Type III underwent a 6 month modified scoliosis specific exercise program with a faded follow up program for another 6 months. The patient initiated PT with a Schroth certified PT in May 2014. From June 2014 to August 2014 -9 direct services sessions and mom videotaped sessions for HEP carryover. HEP was recommended 4-5 x/ week 20-30 min. Three follow up sessions occurred by the end of October and 3 more by April 2015. Re- evaluation was done in April 2015. X Rays were obtained in May 2014, Oct 2014, and April 2015. From evaluation to re-evaluation, the patient grew 4 inches. Treatment sessions focused on pelvic corrections, auto elongation (kneel and reach, stand and reach progressed, sit and reach), passive pad placement in supine position and side lying. Only active corrections were used with predominantly elongation, expansion, asymmetrical sagittal straightening, and normal/ deep breathing while maintaining alignment. The program primarily focused on core strengthening with isometric exercises. The physical therapist consultation was used with school PT to improve transitions (i.e. from sit to stand) to prevent collapse into the major lumbar concavity. Adjunctive treatment: Overnight overcorrecting brace to address a lumbar curve.

**Results and discussion**

The Cobb angles measured were as follows: April 2014- thoracic: 19° and Lumbar: 30°, Oct 2014- thoracic: 5° and Lumbar: 20 ° and April 2015- thoracic: 20° and Lumbar: 31°.

**Conclusion and significance**

A modified Schroth program, in conjunction with bracing, was helpful to initially decrease the curve, but with a major growth spurt during the 6 month follow up, the curves did progress to what the Cobb was originally within +/- 3 degrees range. Based on studies, the general anticipated progression of the curve for SMA patients is ~ 12 °/ year and if looking at the x-rays taken at the year mark, it can be reported that in this case, even with major growth, the scoliosis progression is within the +/- 3 degrees’ error margin.

Informed consent has been obtained from the patient for publication.

## P13 The effectiveness of Scroth based physical therapy and a modified pilates program on an adult with idiopathic scoliosis: a case report

### Lise Stolze

#### Stolze Therapies, Denver, CO, USA

**Introduction**

The adult with Idiopathic Scoliosis (IS) often chooses Pilates as a fitness option due to its emphasis on mind-body awareness and its reputation as a therapeutic modality. Physiotherapeutic Scoliosis Specific Exercise (PSSE) has been recognized by the Scoliosis Research Society as a viable conservative approach to the treatment of Scoliosis. The adult patient with IS may benefit from the integration of these exercise systems.

**Objectives**

The purpose of this study was to explore the effects of Physiotherapeutic Scoliosis Specific Exercises (PSSE) based on the Schroth Method in conjunction with a modified Pilates program on an adult with Idiopathic Scoliosis (IS).

**Methods**

The subject is a 42-year-old woman with IS who experiences pain in her thoraco-lumbar convexity limiting her activities of daily living and recreation. The subject volunteered to enter a 12-week study utilizing PSSE based on the Schroth Method in conjunction with a Pilates program, modified for her scoliosis. The subject has signed an informed consent for publication. Sessions took place 2x per week and lasted 60 minutes. The treating therapist is a Schroth trained physical therapist through the Barcelona Scoliosis Physical Therapy School and Schroth 3 Dimensional Method. She is a certified Pilates instructor through the Pilates Method Alliance and educator for Polestar Pilates. A full spine X-ray was obtained confirming a double curve: left lumbar Cobb angle 30 degrees and right thoracic Cobb angle19 degrees. The subject received the following pre-tests and post-tests at 12 weeks: Scoliosis Special Test: Angle of Trunk Rotation (ATR) using Scoliometer. Functional Tests: Chest Wall Expansion, Diaphragmatic Excursion, Forced Vital Capacity (FVC), Timed Single Limb Stance. Subjective Tests: Quality of Life score using SRS 22r Questionnaire; Pain score using the Visual Analog Scale (VAS). Strength/Endurance Test: Side Support Test. ROM: Shoulder Flexion AROM (Supine); Hip Passive Rotation ROM Test (Prone).

**Results and discussion**

Post test results at 12 weeks showed improvement in the following areas: ATR: -2 degrees at thoracic curve apex and -3 degrees at Lumbar curve apex; Chest Wall Expansion: (subaxillary +1.5 cm, xyphoid +2 cm); Diaphragmatic Excursion: +1.5 cm; FVC +.4 liters; Timed Single Leg Stance left +19 seconds; VAS: -2 points; SRS 22r Questionnaire: +2 points. ROM and strength/endurance tests were unchanged. PSSE based on the Schroth Method in conjunction with a modified Pilates program appears to reduce pain in an adult with IS while improving balance, respiration and overall quality of life parameters.

**Conclusion and significance**

Research on physical therapy using PSSE has demonstrated positive results in adolescent and adult patients with IS. Since adult patients with IS frequently choose a Pilates exercise program as their fitness option, more research is needed to study the effects of a modified Pilates program in conjunction with PSSE as part of a comprehensive approach to the adult patient with IS.

## P14 Spine stabilization exercises improves shoulder and pelvic symmetry in persons with adolescent idiopathic scoliosis

### Marissa Selthafner^1^, Kaitlin Hong^1^, XueCheng Liu^2^, John Thometz^2^, Channing Tassone^2^

#### ^1^Department of Physical Therapy, Children's Hospital of Wisconsin, Milwaukee, USA; ^2^Department of Orthopaedic Surgery, Children's Hospital of Wisconsin, Medical College of Wisconsin, Milwaukee, USA

##### **Correspondence:** Marissa Selthafner

**Introduction**

There is a paucity of published research investigating the role of Physical Therapy interventions for treatment of Adolescent Idiopathic Scoliosis (AIS). Physical Therapists have historically utilized therapeutic exercise as a foundation of their treatment approach for this population. Spine stabilization exercises have been considered a potentially effective treatment option to prevent curve progression, pain and improve respiratory function. However, only a few clinical studies investigate how these same exercises also affect aesthetics, including shoulder and pelvic asymmetry, and rib hump.

**Objectives**

To observe changes in shoulder and pelvic symmetry after 8 weeks of targeted spinal strengthening exercises in patients with adolescent idiopathic scoliosis.

**Methods**

The subject was a 13-year-old female recently diagnosed with Adolescent Idiopathic Scoliosis, Risser Stage 3. She was seen for 8 visits and issued a set of spine stabilization exercises to be performed at least 1x/day. Once the subject was able to demonstrate proper mechanics at 4 weeks, scoliosis-specific postures/exercises were implemented. At the completion of 8 weeks, she was instructed to continue her HEP at least 5 times a week until she reached spine maturity, Risser Stage 5.

**Results and discussion**

3-D Topography was used to measure shoulder height (POTSI score index), rib hump or axial rotation (Suzuki sum index), and pelvic obliquity (measured in degrees). Her POTSI score index improved by 50%, the Suzuki sum index decreased from 12 to 9 (normal range is 0-10), and her initial 3.5 degrees of pelvic obliquity resolved.

**Conclusion and significance**

Improvements in shoulder and pelvic symmetry were observed in this patient who received spine and scoliosis specific exercises over a ten week course. A Boston Brace was also prescribed, but the family deferred this option due to limitations associated with pre-morbid conditions. It is unclear if concomitant brace wear would have made a significant difference in the coronal plane and overall curve management. This case study highlights the importance of aesthetics in addition to curve management. A physical therapy exercise protocol to improve shoulder, rib cage, and pelvic asymmetry should be designed and validated in the future.

Informed consent has been obtained from the patient for publication.

## P15 Test/Retest reliability of surface topography to quantify global spinal posture in middle aged and older adults

### Pamela R. Morrison^1^, Timothy A. Hanke^2^, Patrick Knott^3^, Nathaniel D. Krumdick^2^

#### ^1^Morrison Physical Therapy, Lake Forest, Illinois, USA; ^2^Midwestern University, Downers Grove, Illinois, USA; ^3^Rosalind Franklin University, North Chicago, Illinois, USA

##### **Correspondence:** Pamela R. Morrison

**Introduction**

There is an increased demand for non-invasive spinal postural measurement tools to quantify physical therapy assessments and to monitor adult spinal pathologies. Existing clinical assessment tools for spinal posture have limitations due to unreliable palpation skills and are selective to regions of the spine and do not provide global assessment of spinal posture. Radiographs for monitoring spinal posture expose individuals to ionizing radiation known to have cumulative carcinogenic effects. Technology using surface topography may provide safe, easy to use, and quantifiable data for documenting standing posture. Reliability data exists on adolescents with idiopathic scoliosis and non-scoliotic adolescents. However, there is limited clinical research on the use of surface topography in an adult patient population.

**Objectives**

To determine reliability coefficients and explore the amount of variability of selected surface topography parameters in an adult patient population (40-70 years).

**Methods**

Test/retest reliability with one tester using middle aged and older adults. Ten volunteers were examined using surface topography by one examiner. A sample of convenience was chosen from an existing patient database from an outpatient physical therapy clinic. Inclusion criteria: male and female adults within the ages of 40 to 70 years of age, must be out of direct patient care for a minimum of 30 days. Exclusion criteria: recent musculoskeletal surgery, neurological disorders affecting standing balance, congenital spinal deformities requiring previous spinal surgery, medication affecting balance, ankylosing spondylitis, subjects with limb amputations, acute herniated cervical, thoracic, or lumbar discs, cardiac, chest or abdominal surgery, injuries to upper quadrant including rotator cuff injuries or clavicle injuries, and acute respiratory infections with cough. Three surface topography scans were performed on two occasions, one week apart. Five spinal parameters were examined: trunk length, kyphotic angle, lordotic angle, sagittal imbalance, and pelvic inclination. Data was analyzed using ANOVA for repeated measures. Mean, standard deviation, and range of scores were determined for all dependent variables. Calculations included Cronbach alpha and intra-class correlation coefficient (ICC) Model 2, k utilizing SPSS statistical software.

**Results and discussion**

Cronbach alpha values for the five spinal parameters are between 0.944 and 0.991. Test/retest reliability for trunk length, sagittal imbalance, pelvic inclination, kyphotic angle, and lordotic angle ICC values range from 0.939 to 0.991.

**Conclusion and significance**

The test/retest reliability of surface topography for five select spinal parameters showed excellent results on a sample of adults in a clinical setting. Surface topography is a reliable measurement tool in ambulatory, middle aged and older adults. Future studies should evaluate reliability and validity of measurements on patients with specific spinal pathologies.

## P16 Is there a measurable relationship between physical activity and back surface topography in young adults

### Nachiappan Chockalingam, Thomas Shannon, Ryan Davenhill, Robert Needham, Vinay Jasani, El-Nasri Ahmed

#### Faculty of Health Sciences, Staffordshire University, Stoke-on-Trent, ST4 2DF UK

##### **Correspondence:** Nachiappan Chockalingam

**Introduction**

Understanding the surface topography of the human back is an important factor in the assessment of spinal disorders. Several previous investigations have described the use of back surface topography not only within the examination of spinal conditions and but also for the longitudinal assessment of clinical interventions. Whilst cosmetic issues might influence the clinical management of conditions like idiopathic scoliosis, a further understanding of the back surface topography in normal young adults might help in making clinical decisions in numerous conditions which involves underlying skeletal deformity. It will also help in designing patient specific, effective interventions in physical medicine.

**Objectives**

The primary objective of this observational study is to investigate the relationship between physical activity and the surface topography of the back and spine with a view to create a normative database.

**Methods**

After necessary ethical approval, a group of 120 university students were recruited to participate in this study. This sample consisted of both males and females between the age of 17 and 20 who were actively engaged in a variety of sport. After recording anthropometric data and the details of their preferred sport along with the frequency of training/ participation in the preferred sport, we recorded the surface topography using a newly developed system. This system made use of the commercially available Microsoft Kinect© and a bespoke software code. We examined the left - right symmetry along with the curvature of the spine. We then correlated this information to the type and frequency of physical activity of the participants.

**Results and discussion**

Results clearly indicate the differences in the back surface topography amongst various groups. Whilst the primary objective of this study is to see if there is any measurable and meaningful relationship between the physical activity and surface differences in the human back, the reported results is the first step to develop a normative database of the topography within young adults. These results will help to design further structured studies in this area and will help in the assessment of physical medicine interventions in various types of clinical conditions involving the spine and back.

**Conclusion and significance**

There is a relationship between back surface topography and physical activity amongst young adults. Further structured studies are warranted to document the clinical usefulness of the findings.

## P17 Current knowledge and skills related to early detection and the management of adolescent idiopathic scoliosis (AIS) in conservative care following a series of workshops among undergraduate chiropractic students at a Canadian chiropractic program

### Anissa St-Jean^1^, Julie Touchette^1^, Shawn Drake^2^, Danica Brousseau^1^, Louise Marcotte^3^, Jean Théroux^4,5,6^, Chantal Doucet^1^

#### ^1^Université du Québec à Trois-Rivières, Trois-Rivières, Québec, Canada; ^2^Arkansas State University, Jonesboro, AR, USA; ^3^Private Practice, Montréal, Canada; ^4^Faculty of Medecine, University of Montreal, Montreal, Québec, Canada; ^5^Research Center, Sainte-Justine University Hospital Center, Montreal, Quebec, Canada; ^6^Murdoch University, School of Health Profession, Murdoch, WA, Australia

##### **Correspondence:** Chantal Doucet

**Introduction**

Numerous research suggest that current knowledge and skills may be suboptimal among medicine and physiotherapy students in Europe and the USA regarding the clinical management of AIS when considering the peer reviewed SOSORT Guidelines.

**Objectives**

To determine the current level of knowledge and skills about AIS detection and clinical management among senior interns trained in a chiropractic program at the Université du Québec à Trois-Rivières (UQTR) outpatient clinic immediately prior to and following a series of specific workshops.

**Methods**

An existing 14-question survey was adapted and transculturally validated by the research committee. Two clinical vignettes were also prepared by the research team. The survey was pilot-tested prior to distribution. A sample (*n* = 39) of senior interns took the online survey pre- and post-workshops, first concerning current knowledge on the detection and management of AIS. In addition, the senior interns were asked to complete the two clinical vignettes pertaining to two different stages of AIS. The data were collected and secured in a database using SurveyMonkey. The categorical variables were compared by using the McNemar test as well as the Sign rank test for the comparison of the medians.

**Results and discussion**

A response rate of 95% (*n* = 37/39) was obtained and three participants were excluded having not completed either the pre- or post-workshop 14-question survey or the vignettes. Questions concerning early detection (*p* ≤ 0.003), clinical pearls on the systematic review (*p* ≤ 0.002), and knowledge on evidence-based use of a scoliometer (*p* ≤ 0.001) were all statistically significant between pre- and post-workshop surveys. It appears that those elements were the most positively impacted by the workshops. However, questions on the existence of guidelines and prevalence of AIS in the general population were not influenced by the workshops.

**Conclusion and significance**

Although the results demonstrate significant improvement in certain aspects of knowledge and skills, a persistent gap remains between the curriculum and the specific training with regards to other elements concerning AIS detection and clinical management related to the SOSORT peer-reviewed Guidelines among the seniors interns enrolled in the chiropractic program at UQTR.

## P18 The role of scintigraphy (SPECT) in the diagnosis and treatment of spondylolysis and pre-spondylolyisis

### Angelo Gabriele Aulisa^1^, Vincenzo Guzzanti^1^, Marco Gordano^1^, Giuseppe Mastantuoni^1^, Lorenzo Aulisa^2^

#### ^1^U.O.C. of Orthopedics and Traumatology, Children's Hospital Bambino Gesù, Institute of Scientific Research, P.zza S. Onofrio 4, Rome, 00165, Italy; ^2^Department of Orthopedics, University Hospital "Agostino Gemelli", Catholic University of the Sacred Heart School of Medicine, Rome, 00168, Italy

##### **Correspondence:** Angelo Gabriele Aulisa

**Introduction**

Spondylolysis is a bone defect in one or both sides of the narrow regions of the neural arch, the pars interarticularis.

The pre-spondylolysis and spondylolysis are one of the most common causes of low back pain in children and adolescents and It is not uncommon to observe a spontaneous repair of lysis. The diagnosis of spondylolysis is classically based on lumbar spine X-rays by frontal, lateral and oblique views, T.C. and R.M.N. These exams allow to make the diagnosis in spondylolysis and providing anatomical details of the injury. Nevertheless, in many cases, particularly in pre-spondylolysis, there could be no radiographic evidence of the lesion. In these cases, scintigraphy (TC 99 and SPECT) permits early detection of these pathologies even when radiographs are negative.

**Objectives**

The aim of the study is to confirm the efficacy of scintigraphy in the diagnosis of spondylolysis and pre-spondylolyisis and, in particolare, evaluate its role in the conservative treatment.

**Methods**

Included in this study were 19 patients with low back pain (age between 12 and 18) 17 of them showed radiographic evidence of spondylolysis. All children have been investigated with x-ray by frontal, lateral and oblique views, bone scintigraphy after the injection of (99 m)Tc-MDP with calculated doses according to their body weights and SPECT in left and right oblique views. In all cases was prescribed conservative treatment (Rigid Brace for three months). After four months of treatment, in the cases positive to scintigraphy were repeated all the exams while in the cases negative at the first exam was repeated only x-ray.

**Results and discussion**

The analysis of the series were suggestive of: - 4 cases of bilateral spondylolysis on X rays but negative scintigraphically; - 5 cases of bilateral spondylolysis on X rays with scintigraphy positive during early (blood pool) and late captation; - 3 cases of unilateral spondylolysis on X rays with scintigraphy positive both to the level of lysis that controlaterally; - 4 cases of unilateral spondylolysis on X-ray with scintigraphy positive at the level of lysis and negative contralaterally; - 2 cases of unilateral pre-spondylolysis negative on X-ray but positive scintigraphically at level of lysis; - 1 case of false positive at scintigraphy (SPECT showed an increased uptake of the vertebral body). In the 4 cases with negative scintigraphy the brace treatment was effective to remove the pain but not to repair the lysis. In 13 cases the scintigraphy became negative to control and X rays showed the recovery of the lysis. In 2 cases the situation unchanged.

**Conclusion and significance**

The Scintigraphy allows you to: - guide the diagnosis (removing false positive and showing lysis before the X-ray); - evaluate the metabolic activity (discriminating injuries that conserve the capability of repair from those chronic "nonunion"); - give indication for treatment (no uptake indication for surgical treatment); - check the results of conservative treatment.

## P19 Trunkal changes in children with mild leg length discrepancy: a surface topography study

### Michail Chandrinos^1^, Theodoros B. Grivas^1^, Vasileios Kechagias^2^

#### ^1^Department of Orthopaedics and Traumatology, "Tzaneio" General Hospital of Piraeus, Piraeus, Greece; ^2^Orthopaedic Surgeon, Athens, Greece

##### **Correspondence:** Theodoros B. Grivas

**Introduction**

An asymmetry in legs' length results in leg length discrepancy (LLD). LLD in a child may result in altered gait and lumbar scoliosis in spine. In the available literature however there is a paucity of assessment of trunkal changes due to mild LLD.

**Objectives**

This report aims to assess trunkal changes due to mild LLD using the surface topography (ST).

**Methods**

The patients: Twenty children, attending the Scoliosis Clinic of the department, 7 boys, 13 girls, 9-15 years of age, range 7,5 - 15, mean 15,5 years, suffering mild LLD were assessed. The LLD was 0.5 to 2 cm, mean 1.2 cm. There was not any traumatic LLD. IRB approval and parental consent for the examination was obtained. The apparatus & the 4-D Formetric (4DF) parameters The Diers 4DF analysis system was used for the measurements and the reading resulted from the 4DF computation system. In coronal plane: The coronal imbalance, the pelvic obliquity, the lateral deviation and the 4DF-scoliosis angle were assessed. In sagittal plane: The sagittal imbalance, the 4DF kyphotic angle, the kyphotic apex, the 4DF lordotic angle, the lordotic apex, the pelvic tilt and the trunk inclination. In the transverse plane: The pelvis rotation, the pelvic torsion, the surface rotation and the 4DF vertebral rotation to the right (deemed +) & 4DF vertebral rotation to the left (deemed -), were assessed. The legs length was measured by a measuring tape with 0,1 cm increments, from the anterior superior iliac spine to the medial malleolus. The LLD reliability study showed intra-rater error 0,08 cm and the inter-rater 0,1 cm respectively. Statistical analysis (SA): The SPSS v.22.0 was used.

**Results and discussion**

The coronal imbalance was 0°-46° mean 11.4°, the pelvic obliquity 0°-15° mean 6.6°, the lateral deviation 0.7 - 12.9 mean 6.44 mm and the 4DF scoliosis angle was 5°-59° mean 17.5°. The sagittal imbalance was -1°-11° mean 4.3°, the kyphotic angle 31°-61° mean 41.5°, the kyphotic apex -236 -85 mean -151 mm, the lordotic angle 15°-51° mean 35.35°, the lordotic apex -412 to -264 mean -324.2 mm, the pelvic tilt 0 - 15 mean 6.6 mm and the trunk inclination 0.1°-11.5° mean 4.5°. The pelvis rotation was 0°-7° mean 2.5°, the pelvic torsion 0°-6° mean 2.35°, the surface rotation 1.1°-14.1° mean 50.6°, the vertebral rotation (+) 0°-15° mean 5.1°, and the vertebral rotation (-) 1°-36° mean 7,7°. The SA revealed that the deviation from zero was statistically significant for the above analyzed parameters. The available information on the effects of LLD on trunkal parameters using ST applies only in simulated LLD and not in children suffering real LLD.

**Conclusion and significance**

This report presents the effects of mild LLD on trunkal changes in the 3 cardinal planes during growth. These changes undoubtedly affect not only the children standing trunkal posture but the gait's economy as well. This report may be a useful basis for further understanding of trunkal changes due to LLD.

## P20 Spine horizontal presentation - another technique for scoliosis top view

### Paweł Głowka^1^, Dominik Gaweł^2^, Bartosz Kasprzak^3^, Michał Nowak^2^, Marek Morzyński^2^, Tomasz Kotwicki^1^

#### ^1^Spine Disorders and Pediatric Orthopedics Department, University of Medical Sciences, Poznan, Poland; ^2^Division of Virtual Engineering, University of Technology, Poznan, Poland; ^3^Department of Biophysics, University of Medical Sciences, Poznan, Poland

##### **Correspondence:** Paweł Głowka

**Introduction**

Morphological analysis of scoliotic spine is commonly based on two-dimensional X-rays: coronal and sagittal. 3-dimensional character of scoliosis raised necessity for analyzing scoliosis in three planes. Two presentations called "true da Vinci projection" and "da Vinci representation", showing the transverse plane deformity seen from the cephalad side, were proposed. However, they need special software and the EOS system which is still expensive and difficult to access. In this study, an alternative way to obtain the horizontal presentation of spine ("da Vinci projection"), named Spine Horizontal Presentation (SHP) is presented.

**Objectives**

The main aim of this study was to check the reproducibility and reliability of defining the localization of central points of vertebrae in space, based on two dimensional X-rays: coronal and sagittal. The second aim was to prepare a user friendly graphical spine presentation - Spine Horizontal Presentation.

**Methods**

Eighty four vertebrae (five CT from Th1 to L5) of patients hospitalized for scoliosis surgery were analyzed. Due to different positions during X-Ray (standing) and CT (supine), the corresponding measurements cannot be directly compared. As a solution, the software creating Digital Reconstructed Radiographs (DRRs) from CT scans was developed to replace regular X-rays with DRRs. Based on the measurements performed on DRRs, the coordinates of all vertebral bodies central points were defined. Next, the geometrical centers of vertebral bodies were determined on CT scans. The reproducibility of measurements was tested with Intraclass Correlation Coefficient (ICC), using *p* = 0.05.

**Results and discussion**

Both the intra-observer reproducibility and inter-observer reliability for vertebral body central point's coordinates (x, y, z) were high for the results obtained based on DRRs, CT scans as well as for comparison DRR vs CT. DRR: ICC for intra-observer reproducibility x ICC = 0.9294, y ICC = 0.9949, z ICC = 0.9963; ICC for inter-observer reliability x ICC = 0.9004, y ICC = 0.9946, z ICC = 0.9969. CT: ICC for intra-observer reproducibility x ICC = 0.9864, y ICC = 0.9973, z ICC = 0.9983; ICC for inter-observer reliability x ICC = 0.9815, y ICC = 0.9960, z ICC = 0.9975. There was no significant difference between CT and DRR for each of the three coordinates x, y, z (t Student test for paired samples, p > 0.05). The coordinates of vertebral central points marked on the plot presented the projection of the spine in the horizontal plane, Spine Horizontal Presentation.

**Conclusion and significance**

A method of determining geometrical central points of vertebral body has been developed. The SHP is a user-friendly presentation, analogue to "true da Vinci projection", showing the relationship of scoliotic vertebrae in transverse plane.

## P21 3D analysis of children asymptomatic spine

### Julie Deceuninck^1^, Jean-Claude Bernard^2^, Cyril Lecante^3^, Eric Berthonnaud^1,4,5^

#### ^1^Laboratoire de Physiologie de l'Exercice (EA4338), Université Jean Monnet, Saint-Etienne, France; ^2^Centre des Massues-Croix Rouge Française, 92 rue Edmond Locard, 69005 Lyon, France; ^3^Ets Lecante, 125 rue Bataille 69008 Lyon, France; ^4^L'Hôpital Nord Ouest Villefranche/Saône, BP 436, 69655 Villefranche/Saône, France; ^5^Group of Applied Research in Orthopedic (GARO), Villefranche/Saône, France

##### **Correspondence:** Julie Deceuninck

**Introduction**

Spine can be represented as a 3D geometrical structure according to the model defined by Berthonnaud et al. from two-dimensional face and profile radiographs.

**Objectives**

The 3D reconstruction can be used for the asymptomatic spine. Usual pelvic and spinal parameters are so measured in 3D and new pelvic parameters as the pelvic tilt (T) or the pelvic asymmetry (B) can be considered.

**Methods**

A cohort of 58 asymptomatic children was retrospectively studied. Every subject benefited from strictly orthogonal face and profile radiographs of spine on a turntable. This 3D approach of asymptomatic spine and pelvis is used to characterize the postural balance of the subject while standing. Age varied between 5 and 17 years old (mean age 12 years). Pelvic and spinal parameters were grouped in 11 classes, with all values drawing a Gauss curve. Sagittal curves were assimilated to planes, the number of which can vary between the subjects, and measured in Cobb degrees on the plane; plane rotation was defined, as well as the number of included vertebrae. This analysis method is then applied for scoliotic deformities.

**Results and discussion**

Pelvic incidence was lower than 28.4¡ in class 1 and higher than 68.7¡ in class 11. Pelvic version was lower than 4.7¡ in class 1 and over -21.6¡ in class 11. Pelvic tilt (T) was measured in degrees in order to get rid of the bias of the distance between the subject and the source of x-rays: it was over 2.5¡ right in class 1 and under -2.5¡ left in class 5. Pelvic asymmetry (B) which represents the position of the sacrum compared to hips from top view, was lower than -7¡ right in class 1 and over 7¡ left in class 5. The number of planes could vary between 3 to 5, and plane rotation could vary of less than 10¡ in class 1 and over 85¡ in class 7. For the lumbar curve (C1), angulation on the plane varied between less than 21.2¡ in class 1 to more than 60.5¡ in class 11, with 4 to 6 vertebrae included in the plane.

**Conclusion and significance**

This study shows how it is important to measure pelvic and spinal parameters in 3D and how important are the variations in healthy children. Extreme values found in our asymptomatic group can blend into values that can be found in patients with spinal pathology. It is essential to apply this kind of analysis to understand spinal deformities’ mechanism and results of orthopaedic treatments.

## P22 Preliminary results of the implementation of a new software-based clinical photographic posture assessment tool (CPPAT) in the clinic: Translating knowledge into practice

### Carole Fortin^1,2^, Jean-François Aubin-Fournier^2^, Josette Bettany-Saltikov^3^, Eric C. Parent^4^, Debbie Ehrmann Feldman^1^, Jean-Claude Bernard^5^

#### ^1^Université de Montréal, Montréal, Canada; ^2^CHU Sainte-Justine, Montréal, Canada; ^3^Teesside University, Middlesbrough, United Kingdom; ^4^University of Alberta, Edmonton, Canada; ^5^Centre Médico-Chirurgical des Massues, Lyon, France

##### **Correspondence:** Carole Fortin

**Introduction**

Implementation of research results into clinical practice is essential to improve health services. Presently, there is a lack of evidence regarding the effectiveness of physiotherapy interventions on posture. This may be due to the lack of evidence-based clinical methods to quantitatively assess posture. This study examines the implementation of CPPAT in different clinical settings.

**Objectives**

1. To implement CPPAT for standardizing posture assessment in clinical practice. 2. To evaluate the success of the implementation by determining the practical usefulness and facilitators/barriers for its adoption.

**Methods**

We recruited 35 clinicians working in public or private institutions in Canada (Montréal, Québec, Edmonton), France (Lyon) and United Kingdom (London, Middlesbrough, Chesterfield). Inclusion criteria were clinicians assessing posture of persons with spinal disorders or posture impairments within their clinical routine and having access to a photographic set-up. Participants received a 4-5 hours training session for photographic acquisitions and data processing with the software program. To complete the training, participants had to use the software to assess the posture of 3 eligible patients (with spinal disorders or posture impairments). Following the training period, participants were asked to record how many cases were assessed with the tool and time spent for marker placement/acquisition of photographs and for data post-processing during a period of three months. After the trial period, participants completed a validated questionnaire. Domains assessed included Perceived ease of use (six items), Perceived usefulness (six items) and Intention to use (six items). Participants also commented on the advantages/disadvantages of the CPPAT and factors facilitating/inhibiting use of the tool.

**Results and discussion**

Preliminary results are reported for eight participants from Québec with half of them working in public institutions. Fifty percent of the participants found it easy to learn or interact with the tool. Seven of the eight (88%) participants indicated that the tool would be useful to assess posture more accurately and objectively and to provide better evidence on their posture exams. Only two (25%) noted that the tool would increase their treatment performance/effectiveness. Fifty percent intended to continue using the tool. The principal facilitator was usefulness to quantify and provide evidence for posture assessment whereas the principal barriers were time required to do the complete analysis of the posture indices and the skill required with the software program.

**Conclusion and significance**

The preliminary results on implementation in clinical practice indicate that the CPPAT is well perceived by clinicians and seen as useful if modifications were made to ease the use of the tool. The CPPAT should contribute to clinical practice by facilitating the quantitative analysis of posture. Complete analysis of our cohort and of facilitators/barriers will help inform the promotion of CPPAT into clinical practice.

## P23 A critical role of abnormal leptin bioavailability in the etiology of adolescent idiopathic scoliosis

### Zhen Liu^1,2^, Wen Zhang^1,2^, Zongshan Hu^1,2^, Weiguo Zhu^1,2^, Mengran Jin^1,2^, Xiao Han^1,2^, Yong Qiu^1,2^, Jack C.Y. Cheng^2,3^, Zezhang Zhu^1,2^

#### ^1^Department of Spine Surgery, Drum Tower Hospital of Nanjing University Medical School, Nanjing, China; ^2^Joint Scoliosis Research Center of the Chinese University of Hong Kong and Nanjing University, Hong Kong, China; ^3^Department of Orthopedics and Traumatology, Chinese University of Hong Kong, Hong Kong, China

##### **Correspondence:** Zezhang Zhu

**Introduction**

There currently exists no consensus whether the level of leptin, sOB-R, adiponectin are altered in adolescent idiopathic scoliosis (AIS) patients, although previous studies claim lower level of leptin in AIS patients than the healthy volunteers.

**Objectives**

To validate the differences of the level of leptin, sOB-R and adiponectin between AIS patients and healthy controls in a large sample size.

**Methods**

Three hundred twenty-four AIS patients and 286 healthy adolescent volunteers were recruited in this study. Anthropometric parameters including age, body height, arm span, body weight, BMI were measured and biochemical parameters including level of leptin, sOB-R and adiponectin were assayed by ELISA in both AIS group and controls. The Cobb angle was then investigated in the AIS group. The anthropometric data and level of biochemical parameters were compared between the AIS group and the controls. The correlation between the biochemical parameters and the age, body weight, height, BMI were analyzed in AIS patients and healthy volunteers, together with the relationship between the scoliosis curve magnitude and biochemical parameters above.

**Results and discussion**

The AIS group was older, with greater body weight, height and BMI than the healthy volunteers. With the use of independent T test combined with multivariate regression analysis, the level of sOB-R was determined to be significantly higher than normal controls (31.6 ± 7.5 ng/ml Vs. 20.6 ± 6.8 μg/mL, *P* <0.001), while the level of leptin and adiponectin between the two cohorts did not show significant differences. In addition, the severity of scoliosis curve was not observed to correlate with the level of the assayed biochemical parameters.

**Conclusion and significance**

This validation study confirms previous findings that the level of soluble leptin receptor is higher in AIS patients than controls, while the concentration of leptin and adiponectin remains unchanged in AIS patients. However further study on the in-depth mechanism of leptin bioavailability in AIS patients is warranted.

## P24 Is the sympathetic nervous system considered as etiopathogenesis of idiopathic scoliosis?

### Zhen Liu^1^, Jing Guo^1^, Tao Wu^1^, Bangping Qian^1^, Zezhang Zhu^1^, Feng Zhu^1^, Jian Jiang^1^, Yong Qiu^1^

#### Spine Surgery, the Affiliated Drum Tower Hospital of Nanjing University Medical School, Nanjing, China

“This abstract has not been included here as it has already been published.”

## P25 The effect of different doses of melatonin on the incidence of scoliosis in bipedal C57BL/6 J mice model

### Xiao Han^1,2^, Zhen Liu^1,2^, Hao Liu^1,2^, Yong Qiu^1,2^, Jing Guo^1,2^, Huang Yan^1,2^, Xu Sun^1,2^, Jack C.Y. Cheng^2,3^, Zezhang Zhu^1,2^

#### ^1^Spine Surgery, Drum Tower Hospital of Nanjing University Medical School, Nanjing, China; ^2^The Joint Scoliosis Research Center of Nanjing University and the Chinese University of Hong Kong, Nanjing, China; ^3^Department of Orthopaedics and Traumatology, The Chinese University of Hong Kong, Hong Kong, China

##### **Correspondence:** Zezhang Zhu

**Introduction**

The lack of endogenous melatonin is one of the most important mechanism for adolescent idiopathic scoliosis. Melatonin-deficient bipedal C57BL/6 J mice was reported to high incidence of scoliosis, meanwhile intraperitoneal injection of melatonin was demonstrated to prevent the development of scoliosis. However, there is a paucity of knowledge concerning the correlation between the effect of intraperitoneal injection of melatonin and its dose.

**Objectives**

To investigate the effect of different doses of melatonin injection on the incidence of scoliosis in bipedal C57BL/6 J mice.

**Methods**

Amputation of forelimbs and tail was performed on 36 female C57BL/6 J mice at the age of 3 weeks. The mice were randomly divided into 3 Groups: Group A consisted of 12 mice serving as controls; Group B consisted of 12 mice receiving intraperitoneal melatonin (4 mg/kg BW) at 19:00 hr daily; and Group C involved 12 mice receiving intraperitoneal injection with melatonin (8 mg/kg BW) at 19:00 hr daily. Radiographs were obtained at 20th week to determine the presence of spinal deformity. The incidence of scoliosis was compared among three groups.

**Results and discussion**

Bipedal ambulation for 20 weeks in C57BL/6 J mice induced scoliosis at a rate of 38.9%. Scoliosis developed in 9 mice of Group A (75%), in 3 mice of Group B (25%), and in 2 mice of Group C (16.7%). A higher incidence of scoliosis was observed in Group A. However, there was no significant difference concerning the incidence between Group B and Group C (*p* > 0.05).

**Conclusion and significance**

Melatonin deficiency in C57BL/6 J bipedal played a crucial role for the development of scoliosis, and intraperitoneal injection with melatonin could prevent the development of scoliosis in this model. However, this effect is not affected by the dose of injected melatonin.

## P26 What do we really know about natural history of idiopathic scoliosis during growth? Results of a systematic review

### Francesca Di Felice^1^, Fabio Zaina^1^, Morena Pitruzzella^1^, Sabrina Donzelli^1^, Stefano Negrini^2,3^

#### ^1^ISICO (Italian Scientific Spine Institute), Milan, Italy; ^2^Department of Clinical and Experimental Sciences, University of Brescia, Brescia, Italy; ^3^IRCCS Fondazione Don Gnocchi, Milan, Italy

##### **Correspondence:** Francesca Di Felice

**Introduction**

The real risk of progression of idiopathic scoliosis is considered to vary during different growing phases, but we don't have solid knowledge. Some old papers rates have been for years considered the most relevant description of progression risk of scoliosis during growth, but more recent data suggest the natural history to be even more aggressive. To our knowledge there is no systematic review in this field.

**Objectives**

The aim of this study is to provide a systematic review of current literature about natural history of scoliosis during growth in order to provide details about the risk of progression.

**Methods**

We perform a systematic review of papers describing the natural history of IS during growth and its progression during growth. We searched the MEDLINE, EMBASE and SCOPUS databases up to November 2015. We also screened reference lists of the eligible studies and narrative reviews. Eligible studies were prospective or retrospective studies that enrolled patients with infantile (IIS), juvenile (JIS) or adolescent IS (AIS) followed up without any treatment from the time of detection. We used standard methodological procedures expected by guidelines for systematic reviews.

**Results and discussion**

From the 1663 citations screened, we assessed 48 full-text articles and included 16 of these (3480 participants). Due to relevant differences among the studies, it was not possible to perform a meta-analysis. Taking separately into account studies regarding the infantile, the juvenile and the adolescent scoliosis, we could find that they are heterogeneous with regards to the most of study characteristics and outcomes. Forty-eight percent of patients affected by IIS showed progression while 52% had spontaneous resolution; the rates of progression varied from 5 to 80%. A curve progression > 5¡ Cobb was noticed in 22% in a mixed group of patients affected by JIS or AIS. Fifty-six percent of patients affected by AIS had a progression of > 5¡. Fifty-two percent of patients from one study had a progression and concluded growth with more than 50¡. Some authors reported the rapidity rate of scoliosis progression, which ranged from 2.2¡ to 9.6¡ Cobb per year. The most of the studies have shown to have confounding factors related to some kind of conservative treatment administered at some point of the follow up period, so a lot of patients were not unconditionally followed until skeletal maturity.

**Conclusion and significance**

Just a few studies represent the real natural history of scoliosis without any confounding factors. The definition of progression varied, and data outcome described differed, preventing from a metanalysis. What was clear from almost all the studies is the risk of progression of the Cobb angle during growth, even if the rate of scoliosis progression is extremely variable among studies. This heterogeneity has implication in fields of clinical practice and research.

## P27 A comparison of two kinematic protocols for thorax segment motion assessment during gait

### Robert A. Needham, Panagiotis Chatzistergos, Nachiappan Chockalingam

#### Staffordshire University, Stoke-on-Trent, UK

##### **Correspondence:** Robert A. Needham

**Introduction**

Optoelectronic motion capture details the complexity of human gait. Markers applied to the skin overlying the spinous processes of the vertebrae provide a non-invasive approach to measure dynamic movement of the spine. In clinical gait analysis, the conventional gait model considers the thorax as a rigid segment that represents movement of the entire trunk. To represent three-dimensional (3D) movement of the thorax markers are placed on the spinous processes and sternum. Markers on the superior and inferior aspect of the sternum can have practical concerns for assessment of female participants, particularly those of a younger age. An alternative approach is to use a 3D cluster that can be applied over a spinous process. However, a comparison between these modelling approaches is yet to be examined.

**Objectives**

The purpose of this preliminary study was to compare kinematic modelling techniques of the thorax and to assess the concurrent validity of the 3D cluster.

**Methods**

One male participant took part in this study. An eighteen camera motion capture system was used to record kinematic data of the thorax and pelvis over 10 walking trials. The thorax and pelvis segments were created in accordance with the International Society of Biomechanics (ISB) guidelines. Individual markers were placed on relevant anatomical landmarks using double sided adhesive tape (DSAT). A 3D cluster consists of 3 markers affixed to a rigid base that are attached to the skin using DSAT. 3D clusters were placed over the spinous process of T1 and T3. Relative movement between the thorax and pelvis segment was analysed.

**Results and discussion**

The preliminary findings revealed minimal differences in the range of motion between the traditional thorax model (ISB) and the 3D cluster attached over T1. Consistency between modelling approaches (ISB versus 3D cluster at T1 only) was also noted in regards to movement patterns and the timing of rotations in the opposite direction across the gait cycle. The previously mentioned observations were evident in all three planes of movement. Since the results represent data from one participant, further participants will be recruited.

**Conclusion and significance**

The application of a 3D cluster on the superior region of the thoracic spine closely relates with range of motion values and movements patterns reported by traditional kinematic modelling techniques of the thorax.

## P28 Asymmetry of the vertebral body and pedicles in the true transverse plane in adolescent idiopathic scoliosis - A CT based study

### Rob C. Brink^1^, Tom P.C. Schlösser^1^, Dino Colo^1^, Koen L. Vincken^2^, Marijn van Stralen^2^, Steve C.N. Hui^3^, Winnie C.W. Chu^3^, Jack C.Y. Cheng^4^, René M. Castelein^1^

#### ^1^Department of Orthopaedic Surgery, University Medical Center Utrecht, Utrecht, The Netherlands; ^2^Image Sciences Institute, University Medical Center Utrecht, Utrecht, The Netherlands; ^3^Department of Imaging & Interventional Radiology, Prince of Wales Hospital, The Chinese University of Hong Kong, Hong Kong, China; ^4^Department of Orthopaedics and Traumatology, Prince of Wales Hospital, The Chinese University of Hong Kong, Hong Kong, China

##### **Correspondence:** Rob C. Brink

“This abstract has not been included here as it has already been published.”

## P29 Prospective clinical study of spine growth modulation using titanium clip-screw device: disc heights at 2 and 3 year follow-up

### Donita I. Bylski-Austrow^1,2^, David L. Glos^1^, Viral V. Jain^1,2^, Joseph E. Reynolds^3^, Peter F. Sturm^1,2^, Eric J. Wall^1,2^

#### ^1^Cincinnati Children's Hospital Medical Center, Cincinnati, OH, USA; ^2^University of Cincinnati, Cincinnati, OH USA; ^3^SpineForm LLC, Cincinnati, OH, USA

##### **Correspondence:** Donita I. Bylski-Austrow

**Introduction**

Scoliosis progression has long been considered to be caused in part by asymmetrical compression of vertebral body physes. Treatment by reversal of those biomechanical conditions remains a goal. In preclinical studies, spine growth was shown to be asymmetrically modifiable using clip-screw implant constructs. Spinal growth modulation using titanium clip/screw devices was then tested in an FDA IDE clinical safety study in children with progressive scoliosis.

**Objectives**

The primary purpose of this study was to determine if disc heights decreased from preoperatively to at 2 years post-operatively in a prospective study of a titanium clip-screw device designed to modulate growth in late juvenile to early adolescent idiopathic scoliosis (AIS).

**Methods**

Six patients with progressive AIS underwent thoracoscopic placement of a titanium clip-screw device (IRB approved). Inclusion criteria were Lenke 1A and 1B single thoracic curves, Cobb angle 25° to 40°, age > 10 years, Risser 0 and open triradiate cartilages. Vertebral and disc heights were measured for every patient and at every instrumented level with visible intervertebral boundaries using radiographic images from a clinical PACS system which were then analyzed using commercial digital imaging software (PhotoShop). A calibration ring included in the pre-op radiographs was used for all linear measurements. Changes in disc height by side were compared using paired 1-tailed t-tests.

**Results and discussion**

Disc heights were measurable in 5 of 6 subjects, with one eliminated due to inability to discern most disc boundaries. Mean curvature (*n* = 5) was 34° (±3°) pre-op and 34° (±16°) at 2 years. Disc heights on concave and convex (treated) curve sides were 4.2 mm (±0.4) and 5.6 mm (±0.8) pre-op, and 4.4 mm (±0.5) and 5.6 mm (±0.8) at 2 years, respectively, for mean increase of 0.2 mm (±0.6) on concave (*p* = 0.25) and mean decrease of 0.1 mm (±0.2) on the convex (*p* = 0.17) side. At 3-year follow-up, the mean disc height decrease from pre-op to 3 years was, for concave 0.2 mm (±0.5) (*p* = 0.26) and for convex 0.1 (±1.3) (*p* = 0.44).

**Conclusion and significance**

The hypothesis that disc heights decreased was not supported. The greatest mean decrease was less than 5%. Limitations include resolution of planar digital images of 3D curves, longitudinal linear measurements based on pre-op calibration scale, and need to eliminate the subject with the most axially rotated, and progressive, curve. To our knowledge, this is the first spine growth modulation clinical study to report longitudinal disc height changes. Correlations between disc heights in the thoracic spine and disc health are not yet well defined. However, these results form a baseline for comparisons between disc heights in controls, AIS, bracing, and experimental models, which will allow for relative assessments of disc height maintenance between the scoliosis disease process and this and related proposed methods of spine growth modulation.

## P30 Comparison of rib hump deformity correction using the rib index in adolescent idiopathic scoliosis patients treated with three generations spinal fusion systems

### Vasilios G. Igoumenou^1^, Panayiotis D. Megaloikonomos^1^, Konstantinos Tsiavos^1^, Georgios N. Panagopoulos^1^, Andreas F. Mavrogenis^1^, Theodoros B. Grivas^2^, Konstantinos Soultanis^1^, Panayiotis J. Papagelopoulos^1^

#### ^1^First Department of Orthopaedics, Athens University Medical School, ATTIKON University Hospital, Athens, Greece; ^2^Department of Trauma and Orthopaedics "Tzaneion" General Hospital of Piraeus, Piraeus, Greece

##### **Correspondence:** Vasilios G. Igoumenou

**Introduction**

Various methods have been introduced for the surgical treatment of adolescent idiopathic scoliosis (AIS), such as the Harrington rods, the multiple hook system, hybrid constructs and full transpedicular screw constructs. However, the rib hump deformity (RHD) cannot be fully corrected even when derotation is applied. This fact raises questions regarding the nature of the scoliogeny.

**Objectives**

The aim of this report is to compare the postoperative RHD correction, after spinal fusion with Harrington rods, hybrid constructs and full pedicle screw systems.

**Methods**

Fifty six patients with AIS treated with posterior spinal fusion without costoplasty are included in this study. 23 patients (19 females and 4 males; mean age, 15.3 years; range, 13-16.5 years) were operated with a full pedicle screw system (group A); 18 patients (16 females and 2 males; mean age, 14.9 years; range, 13.2-17 years) operated with a hybrid construct (group B); and 15 female patients (mean age, 16.2 years; range, 13.7-17.5 years) were operated with the Harrington rod system (group C). The RHD correction was assessed by calculating the preoperative and postoperative Rib Index (RI) and RI Correction (%) as described by Grivas (2002). The SPSS Statistics v19 was used; the values of skewness and kurtosis showed that the sample was normal, therefore parametric statistical methods were applied.

**Results and discussion**

The preoperative mean RI was 1.99 (range, 1.49-2.50), 1.99 (range, 1.65-2.30) and 2.12 (range, 1.89-2.52) for groups A, B and C, respectively, while the postoperative mean RI for groups A, B and C was 1.36 (range, 1.20-1.65), 1.42 (range, 1.25-1.59) and 1.52 (range, 1.40-1.77) respectively. The mean RI correction was 30.6% (range, 13.6-39.5%), 28.2% (range, 19.3-33.8%) and 28% (range 22.5-35%) for groups A, B and C, respectively. RI correction was significant in all groups (*p* < 0.001), therefore all systems provided a significant RHD correction. However, RI correction was not significantly different among the three systems (*p* > 0.05).

**Conclusion and significance**

All three spinal fusion constructs offer significant correction of the thoracic deformity with respect to the RI. It is generally assumed though, that the RHD results almost solely from the rotational deformity of the spine. Provided that the full transpedicular screw construct is biomechanically strong allowing for vertebral derotation and direct transverse plane corrections, it was expected the RI correction and RHD correction in group A to be statistically significantly higher. However, this was not the case in this study. Therefore, it is implied that the RHD does not depend only on spinal column deformity, as was widely considered, and furthermore that it results mainly or additionally from the thoracic deformity (asymmetry of the ribs). As it appears from the results of this study the scoliogeny is open to discussion. These results and previous research on the issue (Grivas 2002), in the posed question "does the thoracic follows the spinal deformity or vice versa?" lead to the vice versa option.

## P31 Monitoring the SSEP of the lateral femoral cutaneous nerve during scoliosis surgery

### Negar Behzadi Fard^1^, Kajsa Duke^1,2^

#### ^1^Department of Mechanical Engineering, University of Alberta, Edmonton, Canada; ^2^Division of Orthopaedic Surgery, University of Alberta, Edmonton, Canada

##### **Correspondence:** Kajsa Duke

**Introduction**

Monitoring the somato sensory evoked potentials (SSEP) and the motor evoked potential has now become common practice during scoliosis surgery to prevent injury. A reduction in amplitude of 50% and latency of 10% is a warning of spinal cord problems. The median nerves and posterior tibial nerves are commonly monitored. Due to the positioning of the patient prone on small cushions for several hours, damage to the lateral femoral cutaneous nerve (LFCN) has been reported in 18%-24% of patients.

**Objectives**

The objective is to monitor the SSEP of the LFCN during scoliosis surgery and determine if a change in the SSEP can be detected in patients with LFCN injury after surgery.

**Methods**

During scoliosis surgery, LFCN SSEP was recorded using a Cadwell Cascade Elite system. Electrodes were placed on the patient’s upper thigh and baseline was taken immediately after anaesthesia. LFCN SSEP data was recorded in 23 patients and a detailed analysis was done in four. Two of them had LFCN issues after surgery and the other two did not have LFCN problem. Cpz-Fz region signals were taken and analyzed for both sides plus Cpz-F3 region signals for right side and Cpz-F4 region signal for left side. During the LFCN SSEP data analysis, some signals are removed as they are obviously noise if recordings are taken while electro-cautery occurred. The remaining signals were grouped and averaged for the beginning, middle and end of the surgery.

**Results and discussion**

The SSEP signals from the LFCN of an awake volunteer were analysed. The onset of the response occurs close to 30 ms and then shows an initial negative deflection just after 40 ms followed by a positive deflection at 55 ms. The peak-to-peak amplitude is around 0.45 microV. A similar signal was expected in the beginning for all scoliosis surgery patients. All of the signals during the surgery were plotted and the noisy data was removed. The data was then grouped and averaged for the beginning, middle and end thirds of the surgery. No difference was observed for the group with LFCN injury and the normal group. Overall the SSEP of the LFCN was small and not reproducible during surgery. This could be due to the effect of anesthetic which is known to lower the amplitude. Also, greater external noise is present in the operating room making the signal to noise ratio large. All four patients had normal neuromonitoring of the other more commonly recorded areas, like the posterior tibial nerves.

**Conclusion and significance**

Monitoring of the LFCN SSEP was reproducible for an awake volunteer. The response begins at the 30 ms mark with an amplitude around 0.45 microV. Data was analysed for four scoliosis surgery patients and the LFCN SSEP was not reproducible. There was a large signal to noise ratio and no difference between the normal group and the group with LFCN injury. At this point in time we do not believe this is a reliable method to monitor LFCN injury during scoliosis surgery. Other methods, like monitoring the patient cushion interface pressure are being considered in parallel work.

## P32 Does intraoperative image guidance decrease pedicle screw-related complications in surgical treatment of adolescent idiopathic scoliosis: A systematic review

### Andrew Chan, Eric C. Parent, Edmond Lou

#### University of Alberta, Edmonton, Canada

##### **Correspondence:** Andrew Chan

**Introduction**

Posterior spinal instrumentation and fusion with pedicle screws is performed to correct spinal deformity in adolescent idiopathic scoliosis (AIS). Precision in inserting pedicle screws is critical to ensure strong attachment of the instrumentation and prevent injury to critical structures. Intraoperative image guidance techniques including CT and navigation have been used to allow visually confirming the accuracy of the screw placements. No systematic review has been done to date in comparing complication rates in surgeries using intraoperative image guidance with free-hand methods.

**Objectives**

To systematically review the complication rates of posterior instrumentation and fusion in AIS, focusing on comparing surgeries using intraoperative imaging to surgeries using free-hand methods.

**Methods**

This systematic review followed PRISMA methodology. A librarian helped design a search strategy involving AIS, posterior spinal fusion and pedicle screws, searching in MEDLINE, EMBASE, CINAHL and Web of Science. Randomized controlled trials (RCTs), cohort studies, and case series with more than 10 patients were included. Abstract duplicates were removed and two reviewers assessed abstracts based on these inclusion criteria: AIS undergoing posterior instrumentation and fusion with pedicle screws. Full-texts were similarly assessed based on detailed inclusion criteria. An extraction form was developed to record the surgical approaches, the usage of imaging and the number and types of complications. Reviewers were trained and assessed calibration articles so that extraction for each full-text can be completed by two individuals. The QUIPS Risk of Bias Assessment was selected for quality appraisal of cohort and observational studies and the Cochrane Collaboration's Risk of Bias Tool was used RCTs. The findings of only high quality studies with adequate reporting of complications from pedicle screws will be summarized in this review.

**Results and discussion**

A total of 3686 references were found, with 2461 articles after removing duplicates. An 81% agreement was obtained between pairs of reviewers of abstracts and after consensus 1180 abstracts were included. At this stage, one reviewer screened, 974 full-texts and 344 were included. Of 225 full-texts screened by two reviewers, 35 had disagreement. Two included articles were used as calibration articles. A cohort study by Ughwanogho in 2012 with high risk of bias for possible attrition and confounding issues, suggested a 26% breach rate with CT-navigation imaging and a 52% breach rate in free-hand surgeries. A case series by Dede in 2014 found 1.25% screw-related complication per patient in free-hand surgeries or a 0.14% complication rate per screw.

**Conclusion and significance**

This systematic review is determining if there is evidence to support the increasing usage of intraoperative imaging in posterior instrumentation and fusion for patients with AIS.

## P33 Surgical versus nonsurgical treatment for lumbar degenerative kyphosis

### Jung Sub Lee, Jong Ki Shin, Tae Sik Goh, Seung Min Son

#### Pusan National University School of Medicine, Busan, South Korea

##### **Correspondence:** Jung Sub Lee

“This abstract has not been included here as it has already been published.”

## P34 Painless deformity of the elderly: retrospective Toei study

### Sho Kobayashi, Daisuke Togawa, Tomohiko Hasegawa, Yu Yamato, Shin Oe, Tomohiro Banno, Yuuki Mihara, Yukihiro Matsuyama

#### Hamamatsu University School of Medicine, Hamamatsu, Japan

##### **Correspondence:** Sho Kobayashi

**Introduction**

Elderly spinal deformity patients most commonly present with pain. We can see some elderly person with spinal deformity who has no back pain. However, there are a little study about these painless deformities.

**Objectives**

The purpose of this study was to clarify the prevalence and feature of painless elderly people with spinal deformity by a community based screening program of musculoskeletal disorder in Toei town (*n* = 441).

**Methods**

A total of 321 participants with age of more than 65 were included in this study. The definition of spinal deformity is a Cobb angle of more than 30 degrees of or sagittal modifier ++ (Pelvic incidence minus lumbar lordosis > 20, Sagittal vertical axis (SVA) > 9.5 cm, Pelvic tilt > 30) according to the Schwab's adult spinal deformity classification. They were divided into 2 groups of painless group or painful group and statically analyzed between 2 groups by unpaired t-tests.

**Results and discussion**

The prevalence of spinal deformity over 65 years old was 30 percent (*n* = 96). The rate of painless elderly spinal deformity was 21.8 percent (*n* = 36). The rate of musculoskeletal ambulation disability (one-leg standing time <15 seconds) for painless deformity was 58%. Radiographical analysis showed lower SVA value, higher sacral slope and low incidence of vertebral deformity and spondylolisthesis in painless group. They also have walking habit and are able to stand on one-leg for longer period. A significant increase in painful group was found between sagittal vertical axis (SVA) and heavy physical labor, gowning, stepping, and fast walking, compared with painless group.

**Conclusion and significance**

Our data from this large case-control study confirms that prevalence is about 20%, painless spinal deformity tend to have stable and balanced spine. Musculoskeletal ambulation Disability was not always associated with pain, therefore single analgesic treatment could not help prevention of symptom progression. In future, preventive intervention was needed to these high risk group.

